# Comparison of village dog and wolf genomes highlights the role of the neural crest in dog domestication

**DOI:** 10.1186/s12915-018-0535-2

**Published:** 2018-06-28

**Authors:** Amanda L. Pendleton, Feichen Shen, Angela M. Taravella, Sarah Emery, Krishna R. Veeramah, Adam R. Boyko, Jeffrey M. Kidd

**Affiliations:** 1 Department of Human Genetics, University of Michigan, Ann Arbor, MI 48109 USA; 2 Department of Ecology and Evolution, Stony Brook University, Stony Brook, NY 11794 USA; 3 Department of Biomedical Sciences, Cornell University, Ithaca, New York, 14853 USA; 4 Department of Computational Medicine and Bioinformatics, University of Michigan, Ann Arbor, MI 48109 USA

**Keywords:** Domestication, Canine, Selection scan, Neural crest, Retinoic acid

## Abstract

**Background:**

Domesticated from gray wolves between 10 and 40 kya in Eurasia, dogs display a vast array of phenotypes that differ from their ancestors, yet mirror other domesticated animal species, a phenomenon known as the domestication syndrome. Here, we use signatures persisting in dog genomes to identify genes and pathways possibly altered by the selective pressures of domestication.

**Results:**

Whole-genome SNP analyses of 43 globally distributed village dogs and 10 wolves differentiated signatures resulting from domestication rather than breed formation. We identified 246 candidate domestication regions containing 10.8 Mb of genome sequence and 429 genes. The regions share haplotypes with ancient dogs, suggesting that the detected signals are not the result of recent selection. Gene enrichments highlight numerous genes linked to neural crest and central nervous system development as well as neurological function. Read depth analysis suggests that copy number variation played a minor role in dog domestication.

**Conclusions:**

Our results identify genes that act early in embryogenesis and can confer phenotypes distinguishing domesticated dogs from wolves, such as tameness, smaller jaws, floppy ears, and diminished craniofacial development as the targets of selection during domestication. These differences reflect the phenotypes of the domestication syndrome, which can be explained by alterations in the migration or activity of neural crest cells during development. We propose that initial selection during early dog domestication was for behavior, a trait influenced by genes which act in the neural crest, which secondarily gave rise to the phenotypes of modern dogs.

**Electronic supplementary material:**

The online version of this article (10.1186/s12915-018-0535-2) contains supplementary material, which is available to authorized users.

## Background

The process of animal domestication by humans was complex and multi-staged, resulting in disparate appearances and behaviors of domesticates relative to their wild ancestors [1–3]. In 1868, Darwin noted that numerous traits are shared among domesticated animals, an observation that has since been classified as the domestication syndrome [4]. This syndrome describes the phenomenon where diverse phenotypes are shared among phylogenetically distinct domesticated species but absent in their wild progenitors. Such traits include increased tameness, shorter muzzles/snouts, smaller teeth, more frequent estrous cycles, floppy ears, reduced brain size, depigmentation of skin or fur, and loss of hair.

During the domestication process, the most desired traits are subject to selection. This selection process may result in detectable genetic signatures such as alterations in allele frequencies [5–11], amino acid substitution patterns [12–14], and linkage disequilibrium patterns [15, 16]. Numerous genome selection scans have been performed within a variety of domesticated animal taxa [5–11, 17], and several genes are highlighted as likely associated with the domestication syndrome. This is not unexpected given that more than a dozen diverse behavioral and complex physical traits fall under the syndrome, making it likely that numerous genes with pleiotropic effects contribute through mechanisms which act early in organismal development [18, 19]. For this reason, the putative role of the neural crest in domestication has gained traction [18, 20, 21]. Alterations in neural crest cells number and function can also influence behavior. For example, the adrenal and pituitary systems, which are derived from neural crest cells, influence aggression and the “fight or flight” behavioral reactions, two responses which are lessened in domesticates [22].

No domestic animal has shared more of its evolutionary history in direct contact with humans than the dog (*Canis lupus familiaris*, also referred to as *Canis familiaris*), living alongside humans for more than ten thousand years since domestication from its ancestor the gray wolf (*Canis lupus*). Despite numerous studies, vigorous debate still persist regarding the location, timing, and number of dog domestication events [23–27]. Several studies [5, 8, 26, 28, 29] using related approaches have attempted to identify genomic regions which are highly differentiated between dogs and wolves, with the goal of identifying candidate targets of selection during domestications (candidate domestication regions, CDRs [5]). In these studies, breed dogs either fully or partially represented dog genetic diversity. Most modern breeds arose ~ 300 years ago [30] and contain only a small portion of the genetic diversity found among the vast majority of extant dogs. Instead, semi-feral village dogs are the most abundant and genetically diverse modern dog populations and have undergone limited targeted selection by humans since initial domestication [24, 31]. These two dog groups represent products of two bottlenecks in the evolution of the domestic dog, the first resulting from the initial domestication of gray wolves, and the second from modern breed formation [32, 33]. Selection scans including breed dog genetic data may therefore confound signatures associated with these two events. Indeed, we recently reported [34] that neither ancient nor modern village dogs could be genetically distinguished from wolves at 18 of 30 previously identified autosomal CDRs [5, 8]. Furthermore, most of these studies employed empirical outlier approaches wherein the extreme tail of differentiated loci is assumed to differ due to the action of selection [35]. Freedman et al. [29] extended these studies through the use of a simulated demographic history to identify loci whose variability is unlikely to result from a neutral population history of bottlenecks and migration. When compared to previous outlier-based studies, most of the regions identified in [29] were novel, and harbored genes in neurological, behavioral, and metabolic pathways.

In this study, we reassess candidate domestication regions in dogs using genome sequence data from a globally diverse collection of village dogs and wolves. First, using methods previously applied to breed dog samples, we show that the use of semi-feral village dogs better captures dog genetic diversity and identifies loci more likely to be truly associated with domestication. Next, we perform a scan for CDRs in village dogs utilizing the XP-CLR statistic, refine our results by requiring shared haplotypes with ancient dogs (> 5000 years old) and present a revised set of pathways altered during dog domestication. Finally, we perform a scan for copy number differences between village dogs and wolves, and identify additional copy number variation at the starch-metabolizing gene *amylase-2b* (*AMY2B*) that is independent of the *AMY2B* tandem expansion previously found in dogs [5, 36–38].

## Results

### Use of village dogs eliminates bias in domestication scans associated with breed formation

#### Comparison using *F*_ST_ outlier approaches

**Fig. 1:**
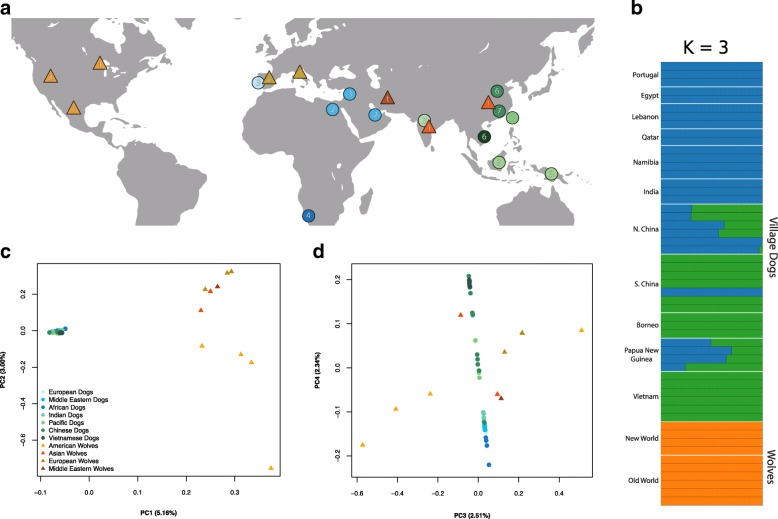
Origin and diversity of sampled village dogs and wolves. **a** The approximate geographic origin of the village dog (circles) and gray wolf (triangles) genome samples included in our analysis. The numbers within each shape indicate the sample count from each population. **b** Admixture plot at *K* = 3 for the filtered village dog (*N* = 43) and gray wolf set (*N* = 10) are shown. Principal component analysis of the filtered sample set at 7,657,272 sites. Results are projected on **c** PC1 and PC2 and **d** PC3 and PC4. Colors in all figures correspond to sample origins and are explained in the PCA legends

Utilizing pooled *F*_ST_ calculations in sliding windows along the genome, two previous studies [5, 8] isolated candidate domestication regions from sample sets consisting of mostly breed dogs and wolves. These loci were classified as statistical outliers based on empirical thresholds (arbitrary *Z* score cutoffs). In order to demonstrate the impact of sample choice (i.e., breed vs village dogs) on the detection of selective signatures associated with early domestication pressures, rather than breed formation, we adapted the methods from these studies and identified outlier loci empirically [5, 8]. First, through ADMIXTURE [39] and identity-by-state (IBS) analyses, we identified a collection of 43 village dog and 10 gray wolf samples (Additional file 1: Table S1) that have less than 5% dog-wolf admixed ancestry and excludes close relatives (Fig. 1a, b; see the “Methods” section). Principal component analysis (PCA) illustrates the genetic separation between village dogs and wolves along PC’s 1 and 2 (Fig. 1c), while positions along PC4 reflect the east-west geographic distribution of the village dog populations (Fig. 1d). To compare directly with previous studies, we calculated average *F*_ST_ values in overlapping 200 kb sliding windows with a step-size of 50 kb across the genome using a pooled approach. As in [5, 8], we performed a *Z* transformation of *F*_ST_ values to normalize the resulting values and identified windows with a ZF_ST_ score greater than 5 (autosomes) or 3 (X chromosome) as candidate domestication regions. Following merging, this outlier procedure identified 31 CDRs encompassing 12.3 Mb of sequence (Additional file 1: Table S2). As in previous studies, a 550 kb region on chromosome 6 (46.80–47.35 Mb) that contains the *pancreatic amylase 2B* (*AMY2B*) and *RNA Binding Region Containing 3* (*RNPC3*) genes had the highest observed average ZF_ST_ score (ZF_ST_ = 7.67).

**Fig. 2:**
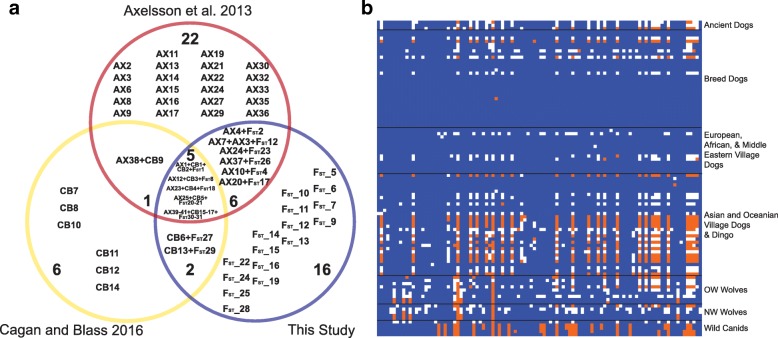
Comparison with previously published candidate domestication regions. **a** Venn diagram depicting counts of intersecting village dog (current study), Axelsson et al. [5] (AX), and Cagan and Blass [8] (CB) candidate domestication regions. Note, some intersecting regions contain multiple loci from a single study; therefore, the counts in this diagram represent the number of genomic regions, not individual loci counts. **b** Genotype matrix for 130 SNPs within chr7: 24,632,211-25,033,464 in AX_14 for 99 canine samples. Sites homozygous for the reference (0/0; blue) and alternate alleles (1/1; orange) are indicated along with heterozygous sites (0/1; white). Each column represents a single SNP, while each row is a sample. Canid groupings are on the right of the matrix

Only 15 of these 31 regions intersect with those reported in [5] and [8] (Fig. 2a). To further explore this discrepancy, we visually assessed whether the dog or wolf haplotype is present at the loci reported in these earlier studies in 46 additional canine samples, including three ancient European dogs ranging in age from 5000 to 7000 years old (see the “Methods” section; [23, 34]). Likely due to the absence of village dogs in their study, some loci identified in Axelsson et al. [5] appear to contain selective sweeps associated with breed formation, as evidenced by the presence of the wild haplotype in ancient and village dogs (example in Fig. 2b). Although all autosomal sweeps identified by [8] intersected with CDRs from our study, seven of their X chromosome windows did not meet the thresholds of significance from our SNP sets (example in Additional file 2: Figure S1). Unlike [8], we performed *F*_ST_ scans and *Z* transformations for windows on autosomes and the X chromosome separately, which may limit false inflation of *F*_ST_ signals on the X that arise due to smaller effective population sizes and correspondingly higher expected levels of genetic drift on the X chromosome. More detailed analysis of the loci highlighted in these two earlier studies [5, 8] will be elaborated in the following section.

#### Refined assessment of previously identified candidate differentiated loci using demographic models and ancient genomes

**Fig. 3:**
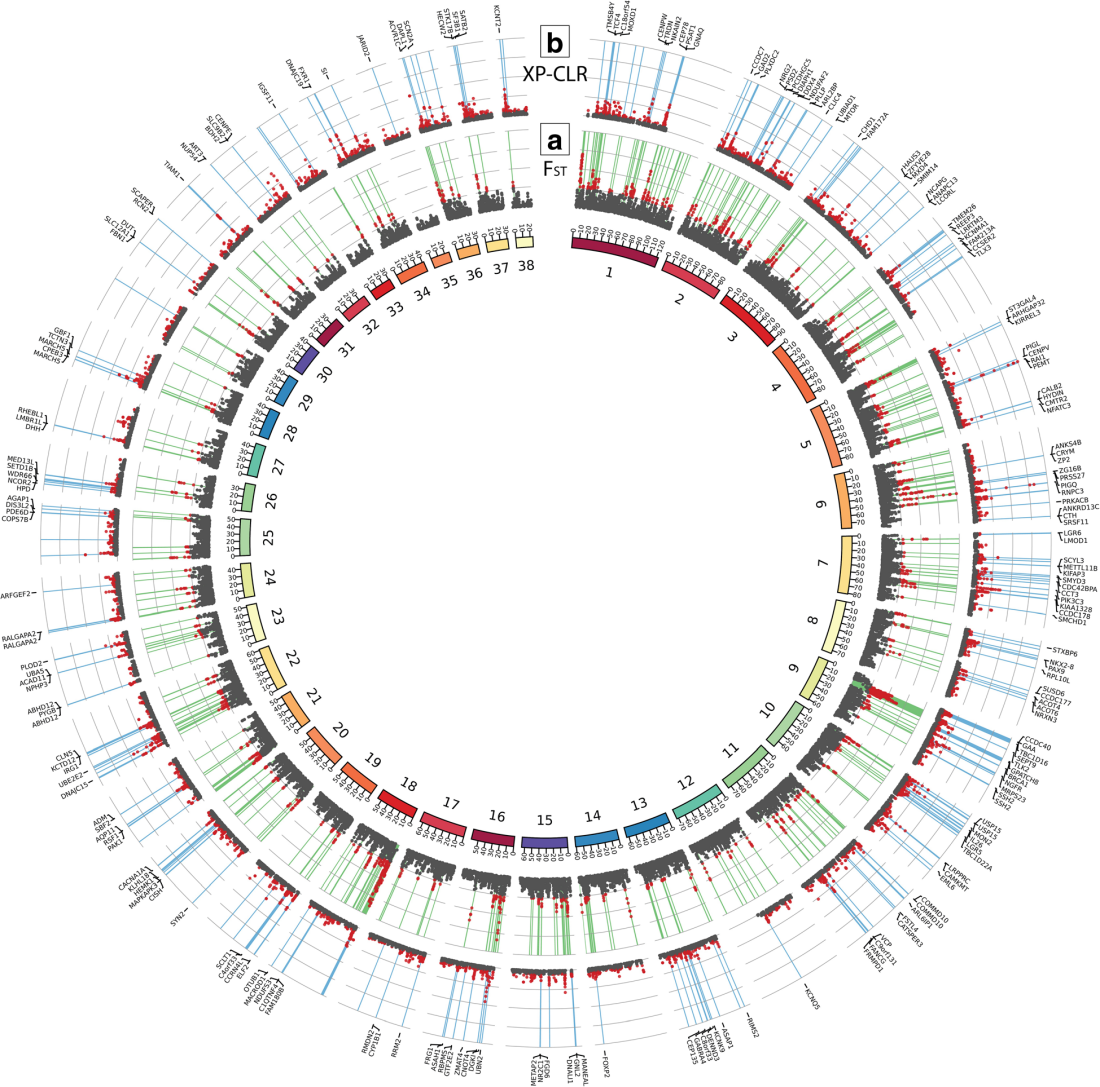
Circos plot of genome-wide selection statistics. Statistics from multiple selection scans are provided across the autosomes (chromosomes identifiers are indicated in the inner circle). (A) Averaged XP-CLR scores in 25 kb windows across the genome. Windows with significant scores (greater than 99th percentile from simulations) are in red, and those that passed filtration are in blue. Genes within significant windows are listed above each region. (B) *F*_ST_ values calculated in 100 kb windows. Values greater than the 99th percentile of simulations are in red. Windows that passed filtration are in green

The above results suggest that the use of village dogs, rather than breed dogs, in selection scans identifies novel candidate domestication regions that are not confounded by breed formation. We developed a statistical filtering strategy to systematically further explore the impact of sample choice on *F*_ST_-based scans. First, rather than setting an empirical threshold at a ZF_ST_ score of 5, we created a neutral null model that captures key aspects of dog and wolf demographic history (Additional file 1: Table S3; Additional file 2: Figure S2; [34, 40]). We identified 443 autosomal sliding windows with *F*_ST_ values that exceed the 99th percentile of the neutral simulations (*F*_ST_ = 0.308; Additional file 2: Figure S3a). Second, reasoning that a true domestication sweep will be largely fixed among extant dogs with no recent wolf admixture, we calculated pooled heterozygosity (*H*_P_) in village dogs within the same window boundaries and retained windows with a *H*_P_ lower than the 0.1th percentile observed in our simulations (Additional file 2: Figure S3b). This heterozygosity filter removed 199 of the 443 windows. Finally, we excluded regions where the putatively selected haplotype is not found in ancient dog samples. To do this, we calculated the difference in dog *H*_P_ (Δ*H*_P_) with and without the inclusion of two ancient dog samples HXH, a 7-ky-old dog from Herxheim, Germany [34] and NGD, a 5-ky-old dog from Newgrange, Ireland [23]; see the “Methods” section). Windows with Δ*H*_P_ greater than the 5th percentile of all windows genome-wide (Δ*H*_P_ = − 0.0036) were removed (Additional file 2: Figures S3c, d and S4). Remaining overlapping windows were merged, resulting in 58 autosomal *F*_ST_ CDRs that encompass 18.65 Mbp of the genome and are within 50 kb of 248 Ensembl gene models (Fig. 3; Additional file 1: Table S4).

We applied the same filtration parameters to the candidate domestication regions identified on the autosomes in Axelsson et al. (*N* = 30; [5]) and Cagan and Blass (*N* = 5; [8]) (Additional file 2: Figure S5a and b). Since window coordinates of these studies may not precisely match our own, we selected the maximum *F*_ST_ value per locus from our village dog and wolf data. We then removed any locus with *F*_ST_, *H*_P_, and Δ*H*_P_ levels not passing our thresholds. Following these three filtration steps, only 14 Axelsson and 4 Cagan and Blass loci remained. In addition, we separately assessed the overlap of our *F*_ST_-based regions with the 349 loci identified by [29] using various statistics and a simulation-based significance threshold which is more comparable to our approach. We found that only 41 of the 349 loci from [29] loci passed our filtrations (Additional file 2: Figure S5c). In total, 25/58 loci identified using *F*_ST_ in village dogs intersected with a putative sweep identified from at least one previous study (for specific overlaps, see Additional file 1: Table S4). The fact that the majority of the previously reported CDRs fail our thresholds when examined in village dogs and ancient dogs suggest that these CDRs reflect selection events that occurred in breeds after dog domestication, rather than true domestication sweeps which should be present in all dogs.

### A scan for the targets of selection during domestication using cross-population haplotype comparisons

To gain a better picture of the targets of selection during dog domestication, we conducted a search for domestication regions in village dogs using XP-CLR, a statistic developed to identify loci under selection based on patterns of correlated multilocus allele frequency differences between two populations [41]. XP-CLR has several advantages over other methods used to identify selection signatures, as it is less biased by demographic history, by uncertainty in recombination rates, and does not maintain strict window boundaries [41]. Instead, the method considers patterns of contiguous SNPs to isolate loci that, based on the size of the affected region, had more rapid correlated changes in allele frequency than expected by genetic drift [41]. Since we are searching for regions under selection in the dog genome, wolves were set as our reference population and XP-CLR was run on both simulated and real SNP datasets with a spacing of 2 kb, and a window size of 50 kb. Average XP-CLR values were calculated within 25 kb sliding windows (10 kb step size) for both datasets, and we retained 889 windows with scores greater than the 99th percentile obtained from simulations (XP-CLR = 19.78; Additional file 2: Figure S6a). Using methods similar to those employed for the *F*_ST_ scans described above, windows with village dog *H*_P_ values less than the 0.1st simulation percentile (*H*_P_ = 0.0598) or where the ancient dog samples carried a different haplotype (Δ*H*_P_ filtration threshold at 5th percentile = − 0.0066) were eliminated (Additional file 2: Figures S6b–d and S3c). This resulted in 598 autosomal windows which we merged into 246 candidate loci, encompassing 10.81 Mb of genomic sequence and within 50 kb of 429 unique genes (Fig. 3b; Additional file 1: Table S5). Of these windows, 178 are located within 50 kb of at least one an Ensembl gene model. No SNPs with high *F*_ST_ within these intervals had predicted deleterious effects on coding sequence. (Additional file 1: Table S6; [42]). The vast majority of the XP-CLR regions (204/246) were not found in previous studies [5, 8, 29], with 4 also found in Axelsson et al. [5] only, 33 in Freedman et al. [29] only and 5 in both Axelsson et al. [5] and Freedman et al. [29]. No loci intersected with the Cagan and Blass [8] findings. Thirty four XP-CLR regions overlap with 21 of the 58 loci we identified using *F*_ST_-based approaches, indicating that XP-CLR often identifies selection signatures within narrower regions.

### Gene content of 246 candidate domestication regions

We sought to identify gene sets and pathways enriched within our candidate domestication regions. Based on 1000 randomized permutations (see the “Methods” section), we found that the XP-CLR regions are not more likely to localize near genes than expected (*p* = 0.07), though the loci are near a greater total number of genes than random permutations (*p* = 0.003; Additional file 2: Figure S7a and b). We observed that our candidate loci contain genes of the similar average length as found in the randomized set (*p* > 0.05; Additional file 2: Figure S7c). The biological functions of numerous genes near the candidate domestication regions are consistent with the neural crest hypothesis, linking this critical embryonic development pathway to the domestication syndrome (Table 1; [18, 20, 21]). Multiple genes are also involved in retinoic acid signaling, neurotransmission, and RNA splicing.

**Table 1:** XP-CLR CDR genes with evidenced or putative roles in nervous system and neural crest pathways

Gene	XP-CLR locus (rank)	System	Phenotypes/effects
*RAI1*	XP 52 (1st)	Xenopus	Mutants display craniofacial defects, improper migration of neural crest cells, decrease in facial cartilage components, axonal defects, and altered forebrain ventricle sizes [119].
*NKAIN2* (*TCBA1*)	XP 9 (4th)	Human	Neurocristopathy-like phenotypes observed in patients with translocation breakpoint in *NKAIN2* such as hair hypopigmentation, craniofacial and limb malformation, misdevelopment of eyes, and macrocephaly [172].
*RNPC3*	XP 57 (8th)	Human	Mutations linked to isolated growth hormone deficiency and pituitary hypoplasia [128].
*NPR2*	XP 127 (14th)	Human, mouse	Mutants exhibit dwarfism and impacted skeletal growth during embryogenesis [173, 174].
*NPHP3*	XP 197 (24th)	Mouse, xenopus	Left/right asymmetry, shortened body axes, and neural folds fail to close in mutants. Interacts with non-canonical *Wnt* pathway [84].
*LIMCH1*	XP 135 (30th)	Human	Significantly altered methylation patterns in Chinese Han pedigrees exhibiting neural tube defects [175]. *LIMCH1* depletion increased cell migration by spatiotemporally regulating non-muscular myosin II activity [176].
*CCDC65*	XP 215 (45th)	Zebrafish	Critical for cilia and dynein function. Knockdowns cause left-right asymmetry and axis curvature embryos [177].
*DAND5* (cerberus-like)	XP 177 (51st)	Mouse	Prevents signaling of the Nodal pathway on the right side of the developing mouse embryo, establishing left/right asymmetry during early somitogenesis [178].
*GBF1*	XP 220 (67th)	Fly	Expressed in embryogenesis, contributes to cell polarity in tubular organs and chemotaxis of neutrophils [179, 180].
*GDPD5*	XP 181 (102nd)	Zebrafish	Regulator of the notch signaling pathway, essential for neural crest pathway, linked to body axis determination [181], is induced by retinoids and drives motor neuron differentiation [182].
HAUS3	XP 38 (111th)	Zebrafish	Essential regulator of embryonic hematopoietic stem/progenitor cell maintenance and cell cycle progression [183].
*PAX9*	XP 80 (114th)	Mouse	Mutants displayed improper craniofacial development, lacked organs deriving from pharyngeal pouches, no teeth [184].
DIAPH1	XP 21 (117th)	Human	Expressed in neural progenitors, linked to microcephaly in humans [185], impacts migration of glioma cells [186].
*TCF4*	XP 3 (127th)	Mouse	Myelinates oligodendrocytes, antagonizes the Wnt signaling pathway, and interacts with *SOX10* (a known neural crest gene [187]) to promote oligodendrocytic maturation gene expression [188].
*TSPAN14*	XP 46 (129th)	Human	Promotes the activity of notch receptors and the expression of *ADAM10* [189], both players in the neural crest signaling pathway [190].
*SATB2*	XP 244 (131st)	Mouse	Mutants exhibit craniofacial abnormalities (e.g., cleft palate, dental misgrowth) and disrupted osteoblast differentiation [99].
*FOXI1*	XP 49 (136th)	Zebrafish	Regulates inner ear and jaw development in embryogenesis, and hypothesized to influence neural crest cell migration and/or separation in the brachial arches [191].
*PRKCAB*	XP 61 (138th)	Mouse	Mutations yield improper development of the neural tube and spina bifida in mice, asymmetric expansion of hedgehog signaling in the neural tube, impact neuronal cell survival [192].
*GNAQ*	XP 16 (141st)	Mouse	Mutants exhibit heart malformations and shortened jaws [193].
Tlx3	XP 48 (146th)	Mouse	Dorsal spinal cord development, specification of glutamatergic neurons [194], and is a target of *Wnt* signaling pathway [59].
*SEMA4A*	XP 70 (150th)	Xenopus	Expressed in neurogenic placodes in the developing neural tube, which along with neural crest cells, migrate to final cell locations [195].
*TIAM1*	XP 225 (154th)	Mouse	With *PAR3*, gene is responsible for determination of front-rear and apical-basal polarity in migratory keratinocyte cells [196].
*PITX1*	XP 124 (157th)	Mouse, anolis	Transcription factor whose binding sites are near key neural crest signaling members (*Wnt*, Hedgehog, *BMP*) [197]. Mutants have improper hind limb development and patterning as well as craniofacial abnormalities [100].
*FKBP8*	XP 175 (167th)	Mouse	Critical for development of the neural tube, establishes dorso-ventral patterning, and prevents apoptosis in embryonic cells in the neural tube [198].
*AMBRA1*	XP 161 (169th)	Mouse	Mutants show disrupted embryonic development, neural tube defects, cell cycle perturbations (unbalanced proliferation and high apoptosis) [199].
*SCUBE1*	XP 115 (202nd)	Mouse	Required for proper development of the central nervous system, neural tube, brain regions, and the cranial vault formation [200].
*CYP1B1*	XP 152 (229th)	Zebrafish	Mutants showed disrupted neural crest migration [201] and is associated with retinoic acid synthesis during the patterning of the developing embryo [50].

Genes within XP-CLR candidate domestication regions (with rank) that have experimental or clinical evidence that illustrate roles in early embryonic pathways, especially in the developing central nervous system and components of the neural crest and its signaling pathways

#### Candidate genes influencing retinoic acid signaling

**Fig. 4:**
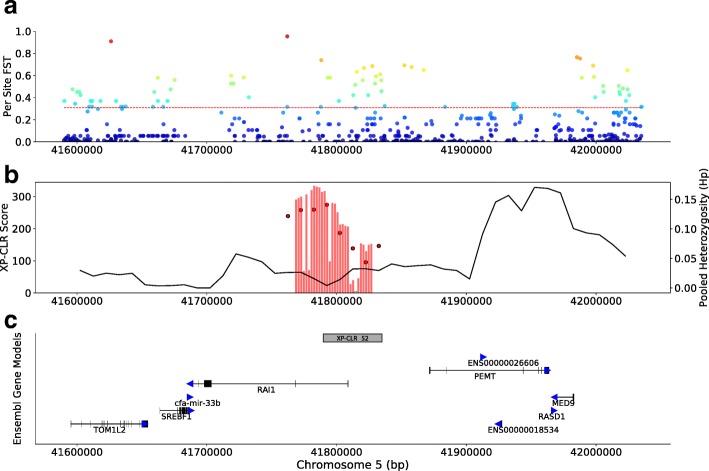
Selection scan statistics at the *RAI1* Locus. Selection scan statistics surrounding the *retinoic acid-induced 1* (*RAI1*) locus (chr5: ~ 41.6-41.2 Mb). **a** Per site *F*_ST_ scores for all SNPs are indicated along with the *F*_ST_ significance threshold determined by the 99th percentile of simulations (red dashed line). **b** Bars represent raw XP-CLR grid scores. Circles indicate the mean XP-CLR score calculated from averaging grid scores within 25 kb windows and are positioned within the center point window. Red bars and circles indicate that the score is significant (above the 99th percentile significance threshold determined through simulations). The black line indicates the average pooled heterozygosity (*H*_P_) values for the same window boundaries. **c** The significant XP-CLR locus (gray box) is presented relative to Ensembl gene models (black). Direction of each gene is indicated with blue arrows

Retinoic acid (RA) is a signaling molecule that has numerous critical roles in development at the embryonic level, continuing into adult stages with roles such as maintaining stem cell proliferation, tissue regeneration, and regulation of circadian rhythm [43, 44]. The highest scoring XP-CLR locus centers upon *RAI1* (*retinoic acid-induced 1*; XP 52; Fig. 4), a gene that has not been identified in previous domestication scans. *RAI1* has numerous developmental functions in the RA pathway, and mutations in this gene are responsible for Smith-Magenis and Potocki-Lupski syndromes in humans [45, 46]. Other genes with related functions include *NR2C1* (XP 143), essential for the development of early retina cells through regulation of early transcription factors that govern retinal progenitor cells such as RA receptors [47] and calreticulin, a protein involved in inhibition of both androgen and RA transcriptional activities [47, 48]. *Ncor2* (XP 209) increases cell sensitivity to RA when knocked out in mice [49], and *CYP1B1* (XP 152) is a pathway component that can direct embryonic patterning by RA [50].

#### Candidate genes regulating brain development and behavior

Twelve XP-CLR candidate genes related to neurotransmitter function include the serotonin transporter *SLC6A4* (XP 101) and dopamine signaling members *GNAQ* (XP 16) and *ADCY6* (XP 215). Genes associated with glutamate, the excitatory neurotransmitter, include *DGKI* (ranked 6th by XP-CLR; XP 145), which regulates presynaptic release in glutamate receptors [51], and *GRIK3* (XP 141), a glutamate receptor [52]. Other genes include *UNC13B*, which is essential for competence of glutamatergic synaptic vesicles [53], and *CACNA1A* (XP 176) influences glutamatergic synaptic transmission [54]. In contrast to glutamate, GABA is the nervous system’s inhibitory neurotransmitter and has been linked to the response to and memory of fear [55, 56]. Genes in our XP-CLR loci relating to GABA include one of the two mammalian GABA biosynthetic enzymes *GAD2* (or *GAD65*; ranked 20th), the GABA receptor *GABRA4*, auxiliary subunit of GABA-B receptors *KCTD12* ([57]), and the GABA inhibitor osteocalcin (or *BGLAP*; [58]). Lastly, *TLX3* (XP 48) is a key switch between glutamatergic and GABAergic cell fates [59].

#### Candidate genes related to RNA splicing

**Fig. 5:**
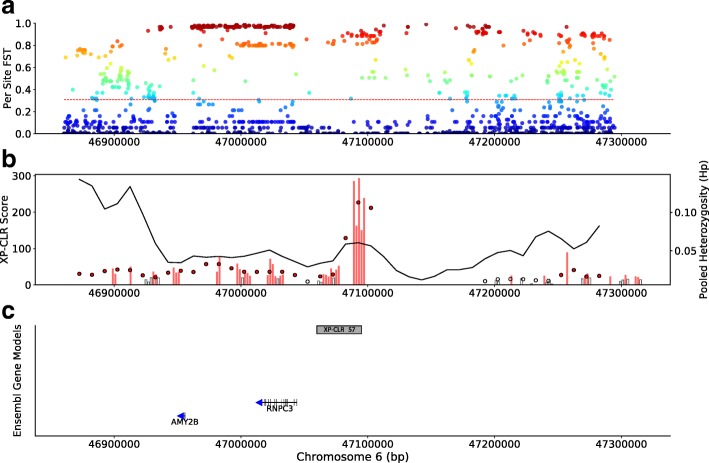
Selection scan statistics at the *RNPC3* locus. Selection scan statistics surrounding the RNA-binding region (*RNP1*, *RRM*) containing 3 (*RNPC3*) locus (chr5: ~ 46.9–47.3 Mb). **a**–**c** as in Fig. 4

We also observe numerous candidate genes involved in splicing of transcripts by both the major and minor splicing pathways. The eighth highest XP-CLR region (XP 57) harbors the gene *RNPC3*, the 65 KDa subunit of the U12 minor spliceosome, which is located ~ 55 kb downstream of pancreatic amylase *AMY2B* (Fig. 5). Another core subunit, *SF3B1*, belongs to both the minor and major (U2) spliceosome. Additional XP-CLR genes related to splicing and/or spliceosome function include *FRG1* [60], *DDX23* (alias *PRP28*; [61]), *CELF1* [62], *NSRP1* (alias *NSrp70*; [63, 64]), and *SRSF11* (alias *P54*; [65]).

### Survey of copy number variation between dogs and wolves

Copy number variants have also been associated with population-specific selection and domestication in a number of species [5, 66, 67]. Since regions showing extensive copy number variation may not be uniquely localized in the genome reference and may have a deficit of SNPs passing our coverage thresholds, we directly estimated copy number along the reference assembly and searched for regions of extreme copy number differences (see the “Methods” section). Using *V*_ST_, a statistic analogous to *F*_ST_ [66], we identified 67 regions of extreme copy number difference between village dogs and wolves which are within 50 kb of 89 unique genes (Additional file 1: Table S7). There was no overlap of these copy number outliers with regions identified through *F*_ST_ or XP-CLR. Relative to randomly permuted intervals, the 67 *V*_ST_ outliers are more likely to be near genes (*p* < 0.01; Additional file 2: Figure S8a) but do not encompass more total genes than expected (p > 0.05; Additional file 2: Figure S8b).

The top locus identified through *V*_ST_ analysis encompasses the *AMY2B* gene, which at increased copy number confers greater starch metabolism efficiency due to higher pancreatic amylase enzyme levels [5, 37]. Quantitative PCR results have suggested an ancient origin for the *AMY2B* copy number expansion, as 7-ky-old Romanian dogs exhibit elevated *AMY2B* copy number [38]. However, read-depth analysis shows that the *AMY2B* tandem expansion is absent in 5–7-ky-old ancient European dogs [34]. We identified two large duplications, one of 1.9 Mb and the other of 2.0 Mb, that encompass *AMY2B* (Additional file 2: Figure S9). We quantified copy number at *AMY2B* itself and regions which discriminate the two segmental duplications in 90 dogs using digital droplet PCR (ddPCR). Copy number estimated through read depth strongly correlated with estimates from ddPCR (Additional file 2: Figure S10) confirming the presence of standing copy number variation of *AMY2B* in dogs (range of 2*n*_*AMY2B*_ = 2–18) and distinguishing the two large-scale duplications (Additional file 2: Figure S11). The extreme *AMY2B* copy number expansion appears to be independent of the large-scale duplications, as ddPCR results show that some dogs without the large duplications still have very high *AMY2B* copy number. Read-depth patterns at the duplication breakpoints indicated that NGD, the ancient Irish dog, harbored the 2.0 Mb duplication resulting in increased *AMY2B* copy number.

### Gene ontology enrichment analysis

We performed enrichment tests using the parent-child model [68] in the topGO R package [69] with the intersecting 429 unique genes as the test set. To control for biasing factors such as gene size, function, and colocalization, we calculated permutation-based *p* values (*p*_perm_) for each GO term by comparing the observed parent-child significance score for each GO term with the distribution obtained by applying the parent-child test to gene sets identified by 1000 randomly permuted genome intervals (see the “Methods” section). We identified 636 enriched GO terms (*p*_perm_ < 0.05) including 327 GO terms represented by more than one gene and more than one XP-CLR locus (Additional file 1: Table S8). The set supported by multiple loci includes several categories related to the process noted above including the regulation of retinoic acid receptors (*p*_perm_ = 0.028), retinol metabolism (*p*_perm_ = 0.014), the secretion (*p*_perm_ = 0.01), transport (*p*_perm_ = 0.01), and signaling of GABA (*p*_perm_ = 0.03), dopamine receptor signaling (*p*_perm_ = 0.04), and cell maturation (*p*_perm_ = 0.012). Similar enrichment results were also observed using EMBL-EBI ontology annotations (see the “Methods” section; Additional file 1: Table S9). Seventy-one enriched (*p*_perm_ < 0.05) categories were identified using the same methods for the 89 genes intersecting the *V*_ST_ (copy number) candidate loci (Additional file 1: Table S10). However, these enrichments were largely driven by a handful of genes with broad biological functions. No enrichments for either XP-CLR or copy number results remain statistically significant if one corrects for the 19,408 tests representing all of the possible GO terms in our gene set, although there are limitations to the application of multiple testing corrections to correlated GO terms.

## Discussion

Genetic and archaeological data indicate that the dog was first domesticated from Eurasian gray wolves well over 10 kya [23, 27, 34, 40]. Evidence suggests that the domestication process was complex and may have spanned thousands of years [3, 23]. Through multiple analyses, we have identified regions that are strongly differentiated between modern village dogs and wolves and which may represent targets of selection during domestication. Our approach differs from previous studies in several ways including the use of village dogs rather than breed dogs, using neutral simulations to set statistical cut-offs, and filtering candidate loci based on ancient dog DNA data. Most (83%) of the 246 candidate domestication regions we identified are novel to our study, which we largely attribute to reduced signals associated with post-domestication breed formation. We argue that swept haplotypes identified in modern village dogs and also present in Neolithic dogs more likely represent signals of ancient selection events. Although the 43 village dogs sampled here do not represent the full spectrum of genetic diversity of modern dogs, these samples largely reflect the diversity found in an extensive panel of canids sampled by SNP array and represent populations estimated to have split over 15 kya (European vs Asian) [34]. We expect true targets of selection associated with domestication to be found across all dogs. Signals restricted to breed dogs, although unlikely to reflect selective pressures during domestication, identify genes and pathways important for understanding the genetic basis of modern dog biology and disease. Deeper sampling of village dog diversity may reveal that the CDRs we identified are unique to the studied samples, perhaps as a potential result of geographically restricted selection. As more village dogs are sequenced, it is likely that these candidate domestication regions will be refined and narrowed.

While the use of neutral simulations accounts for genetic diversity in both wild and domestic sampled populations, and better controls false positive rates than arbitrary empirical thresholds [29, 70], several limitations are still apparent in our approach. The demographic model we used does not capture all aspects of dog history, does not include the X chromosome, and does not fit all aspects of the observed data equally well. This likely represents unaccounted for features of the data, such as unmodeled population structure, as well as technical issues such as reduced ascertainment of low frequency alleles due to sequencing depth. Although previous studies have identified detectable jackal admixture ranging from 1 to 2% in the ancestral dog population, we did not include the jackal in our demographic model. Since this gene flow occurred in the ancestral lineage of both modern dogs and wolves (> 20 kya) [32, 34, 40] the jackal ancestry is expected to be similarly represented in all of our samples. This assumption may not hold if the ancestral population had a high degree of population structure, but suitable data to model such complexities is not available.

Although the inclusion of ancient samples allows for the removal of candidate domestication regions that are unique to modern dogs, this approach is limited by the narrow temporal (5–7 kya) and geographic (restricted to Europe) sampling offered by the available data. Even though most selected alleles likely preexisted in the ancestral wolf population, our approach identifies regions where modern village dogs share the same haplotype. However, even when selection acts on preexisting mutation, a single haplotype often reaches fixation [71], consistent with the variation patterns we identify across village dog populations. As the amount of ancient dogs with genome data increase, it will become possible to apply sophisticated tests that make direct use of ancient genomes to discover sites of selection [72, 73].

Our gene annotations were obtained directly through established BLAST2GO pipelines [74]. Similar results, although with fewer gene-function links, were obtained when using the Ensembl Release 92 of the EMBL-EBI GO gene annotations (Additional file 1: Table S10). After correcting for a total of 19,408 possible tests, none of our enrichments would be significant, even if the raw parent-child *p* values were used. However, several factors complicate these gene set enrichment tests. First, the nature of the GO ontology relationships introduces non-independence among related GO terms and genes, a problem partially ameliorated by the parent-child model [68]. Second, the underlying statistical tests assume that every gene is equally likely to be a member of the test set under the null hypothesis, an assumption that may be reasonable for studies of gene expression. Our permutation strategy attempts to control for the non-random correlation between gene size, colocalization, and gene function. However, since no GO term survives a global multiple testing correction, these enrichments must be viewed as tentative.

### The role of the neural crest in dog domestication

Our XP-CLR candidate domestication regions include 52 genes that were also identified in analyses of other domesticated or self-domesticated animals [9, 11, 17, 75–79], including four genes (*RNPC3*, *CUEDC1*, *GBA2*, *NPR2*) in our top 20 XP-CLR loci. No gene was found in more than three species, consistent with the hypothesis that no single domestication gene exists [19]. Although the overlap of specific genes across species is modest, there are many enriched gene pathways and ontologies shared in domesticates including neurological and nervous system development, behavior, reproduction, metabolism, and pigmentation [10, 11, 17, 73, 75, 80]. We attribute these patterns to the domestication syndrome, a phenomenon where diverse traits, manifested in vastly different anatomical zones, appear seemingly disconnected, yet are maintained across domesticates. Two possible modes of action could generate the domestication syndrome phenotypes while still displaying the genome-wide distribution of sweeps. The first would require independent selection events for distinct traits at numerous loci. Alternatively, selection could have acted on considerably fewer genes that are members of early-acting developmental pathways with broad phenotypic effects.

For these reasons, the role of the neural crest in animal domestication has gained support from researchers over recent years [18, 20, 21] (Table 1). In 2014, Wilkins et al. [18] established that the vast array of phenotypes displayed in the animal domestication syndrome mirror those exhibited in mild human neurocristopathies, whose pathology stems from aberrant differentiation, division, survival, and altered migration of neural crest cells (NCCs). These cells are multipotent, transient, embryonic stem cells that are initially located at the crest (or dorsal border) of the neural tube. The initiation and regulation of neural crest development is a multi-stage process requiring the actions of many early-expressed genes including the fibroblast growth factor (*Fgf*), bone morphogenic protein (*Bmp*), wingless (*Wnt*), and *Zic* gene families [81]. Several of the genes identified in our XP-CLR analysis are involved in this transition including members of the Fgf (*Fgf1*) family as well as a transcription factor (*TCF4*; [82]), inhibitors (*RRM2*; *NPHP3*; [83, 84]), and regulators (*LGR5;* [85]) of the Wnt signaling pathways.

Following induction, NCCs migrate along defined pathways to various sites in the developing embryo. Assignment of identity and the determination of migration routes rely on positional information provided by external signaling cues [86, 87]. *KCTD12*, *CLIC4*, *PAK1*, *NCOR2*, *DOCK2*, and *EXOC7* are all examples of such genes found in our candidate loci that are linked to the determination of symmetry, polarity, and/or axis specification [88–92]. Together, our results suggest that early selection may have acted on genes essential to the initiation of the neural crest and the definition of migration routes for NCCs.

#### NCC-derived tissues linked to domestication syndrome phenotypes

Once in their final destinations, NCC further differentiates as the precursors to many tissues in the developing embryo. Most of the head, for example, arises from NCCs including craniofacial bones, cartilage, and teeth [93, 94]. Ancient dog remains indicate that body size, snout lengths, and cranial proportions of dogs considerably decreased compared to the wolf ancestral state following early domestication [95]. Further, these remains indicate jaw size reduction also occurred, as evidenced by tooth crowding [95]. Such alterations are consistent with the domestication syndrome and implicate aberrant NCC migration since decreases in the number of NCCs in facial primordia are directly correlated with reductions in mid-face and jaw sizes [18, 96]. Genes associated with both craniofacial and tooth development in vertebrates are found in our candidate loci including *SCUBE1* (XP 115), which is essential in craniofacial development of mice, and *SATB2* (XP 244), which has roles in patterning of the developing branchial arches, palate fusion, and regulation of *HOXa2* in the developing neural crest [97–99]. Lastly, when knocked out in mice, *Bicoid*-related homeodomain factor *PITX1* (XP 124) not only affected hindlimb growth, but also displayed craniofacial abnormalities such as cleft palate and branchial arch defects [100], and influences vertebrate tooth development [101].

Insufficient cartilage, a NCC-derived tissue [94] that consists of chondrocytes and collagen, in the outer ear of humans results in a drooping ear phenotype linked to numerous NC-associated neurocristopathies (e.g., Treacher Collins and Mowat-Wilson) [102]. Analogously, compared to the pricked ears of wolves, dogs predominantly have “floppy” ears [103], a hallmark feature of domesticates [18]. Ablation of *SERPINH1* (XP 181), a collagen-binding protein found in our list of CDRs, is embryonically lethal in ablated in mice [104] and appears to be required for chrondrocyte maturation [105]. Alterations of activity by genes such as *SERPINH1* and those regulating NCC migration may have reduced the numbers of NCCs in dog ears, contributing to the floppy phenotype [18].

#### Genes associated with neurological signaling, circadian rhythms, and behavior

Tameness or reduced fear toward humans was likely the earliest trait selected for by humans during domestication [3, 106, 107]. Recapitulating such selection, numerous physiological and morphological characteristics, including domestication syndrome phenotypes (i.e., floppy ears, altered craniofacial proportions, and unseasonal timing for mating), appeared within 20 generations when researchers selected only for tameness in a silver fox breeding population [1, 108]. As the progenitors for the adrenal medulla, which produces hormones associated with the “fight-or-flight” response, hypofunction of NCCs can lead to changes in the tameness of animals [18]. The link between tameness and the NC suggests that changes in neural crest development could have arisen first, either through direct selection by humans for desired behaviors or via the “self-domestication” [109, 110] of wolves that were more docile around humans. Genes contributing to neurological function and behavioral responses were observed in our XP-CLR candidate loci, suggesting these genes may influence chemical and morphological differences associated with tameness. Numerous candidate loci contain genes influencing neurological function and behavioral responses including genes in the dopamine, serotonin, glutamate, and GABA neurotransmission pathways, as well as genes contributing to the connectivity and development of synapses and dendrites.

In addition to changes in behavior, alterations in sleep patterns would also likely have occurred early in the domestication process due to the shift from the ancestral nocturnal state of wolves, to that of the diurnal lifestyle also exhibited by humans. Evidenced by this, levels of circadian rhythm determinants (e.g., melatonin and serotonin) were significantly altered in domesticated silver foxes selected for tameness compared to wild foxes [111–113]. We hypothesize that early selection on genes influencing behavior have additional functions in the establishment of circadian rhythms, and that both can be explained by impaired NC function. The Smith-Magenis syndrome is caused by disrupted function of *RAI1* [114], the gene with the highest XP-CLR score in our study. Humans with Smith-Magenis syndrome display increased aggression and altered circadian rhythms, as well as craniofacial and skeletal deformations, developmental delays, and intellectual disabilities [115]. Similarly, Williams-Beuren syndrome, another neurodevelopmental disorder, affects sleep patterns as well as contributes to hypersociability in humans [116]. A recent study in canines linked behavioral changes in breed dogs to structural variants near *WBSCR17*, a Williams-Beuren syndrome gene [117]. Both syndromes display multiple features associated with improper NCC development, resembling phenotypes of neurocristopathies [115, 118]. For example, disruption of the transcription factors *RAI1* and *WSTF* in xenopus (also disrupted in Williams-Beuren syndrome) negatively impacts proper NCC migration, recapitulating the human craniofacial defects associated with the syndromes [119, 120]. *RAI1* also regulates circadian rhythms [121–124], a pathway within which other XP-CLR candidate loci genes also exhibit possible (*RNPC3*; [125, 126]) and experimentally verified (*FBLX3*; [127]) roles. Altogether, the top scoring locus, as well as others, indicate overlap of gene functions in influencing behavior and circadian rhythms, and were likely early genetic components of the domestication syndrome.

#### Misregulation of gene expression may contribute to domestication syndrome phenotypes

Similar to other domestication scans [6, 9, 19], we did not find SNPs deleteriously altering protein sequence in our predicted sweeps, indicating that gene loss did not have a significant role in dog domestication. Instead, we hypothesize that alterations in gene regulatory pathways or the regulation of transcriptional activity could contribute to broad domestication syndrome phenotypes. Our gene list includes two components of the minor spliceosome; *RNPC3* and *Sf3b1*. *RNPC3*, which affects early development and is linked to dwarfism (isolated growth hormone deficiency; [128]), is also under selection in cats and humans [17, 77]. Absence of *Sf3b1* disrupts proper NCC specification, survival, and migration [129]. A further example of the role of splicing in NC development is that mutations in *U4atac*, a U12 snRNA subunit gene missing in the current dog annotation, causes Taybi-Lindner syndrome (TALS) in humans. Phenotypes of this syndrome resemble those of the domestication syndrome including craniofacial, brain, and skeletal abnormalities [130]. Thus, proper splicing, particularly for transcripts processed by the minor spliceosome, is required for proper NC function and development.

#### Copy number variation was likely not a major driver during dog domestication

Our scan for differentiated copy number states identified few regions that differentiate village dogs and wolves. A previous study found that dogs and wolves have a similar proportion of CNV loci [131]. This suggests that copy number expansion or contraction may not have made as significant contributions to the phenotypic changes associated with domestication. The quantification of wolf copy number using a dog genome reference limits the accuracy of the estimates and prevents detection of wolf-specific insertions. Therefore, reassessment of population-specific copy number changes would be improved by the use of a wolf genome reference [132]. Of note, the top hit from the copy number selection scan corresponded to the *AMY2B*, a gene linked to increased efficiency of starch digestion in dogs [5, 36, 37]. Previous studies have concluded that the increase in *AMY2B* copy number occurred post-domestication, since the timing of domestication (> 10 kya) predates the introduction of starch-rich diets in both humans and dogs [32, 34, 36]. However, this study utilizes previously implemented copy number estimation techniques [34, 36] to identify two independent large-scale duplications (1.9 and 2.0 Mb) that are at least the age of the oldest sampled dog genome (7 ky old). Significant selection signatures from XP-CLR are distal to *AMY2B*, instead centered on *RNPC3* (discussed above) which also lies within the boundaries of both large duplications. Since these large duplications are not fixed in dogs, yet the *RNPC3* selected haplotypes are, we speculate that the initial target of selection may have been on *RNPC3* which could have global effects on expression and phenotype (body size).

## Conclusions

By comparing village dogs and wolves, we identified 246 candidate domestication regions in the dog genome. Analysis of gene function in these regions suggests that perturbation of crucial neural crest signaling pathways could result in the broad phenotypes associated with the domestication syndrome. Additionally, these findings suggest links between transcriptional regulation and splicing to alterations in cell differentiation, migration, and neural crest development. Altogether, we conclude that while primary selection during domestication likely targeted tameness, genes that contribute to determination of this behavioral change are also involved in critical, far-reaching pathways that conferred drastic phenotypic changes in dogs relative to their wild counterparts.

## Methods

### Sample processing and population structure analysis

The primary selection scans in this paper are based on 43 village dog and 10 gray wolf samples selected from a larger sample set as described below. Additional analysis of candidate genomic regions is based on genotype data from two ancient European samples. For visualization purposes, Fig. 1 also includes genotype data from a larger collection of breed dogs and wild canid out groups. Canid genomes (Additional file 1: Table S1) were processed using the pipeline outlined in [34] to produce a data set of single nucleotide polymorphisms (SNPs) using GATK [133]. From this larger sample set, 37 breed dogs, 45 village dogs, and 12 wolves were selected from the samples described in [34], and ADMIXTURE [39] was utilized to estimate the levels of wolf-dog admixture within this subset. This sample set includes three New Guinea Singing Dogs sequenced as described in [134]. To account for LD, the data was thinned with PLINK v1.07 (--indep-pairwise 50 10 0.1; [135]), where SNPs with an *R*^2^ value over 0.1 were removed in 50 kb windows, sliding 10 sites at a time. The remaining 1,030,234 SNPs were used in five independent ADMIXTURE runs using different seeds, for up to five ancestral populations (*K* = 1–5). *K* = 3 had the lowest average cross validation error (0.0373) from the five runs and was therefore the best fit for the data (Additional file 2: Figure S12). To eliminate noise in subsequent analyses, we removed all village dogs with greater than 5% wolf ancestry and wolves with greater than 5% dog ancestry. Fifty-four samples remained following this filtration.

Following elimination of admixed samples, we called SNPs in 43 village dogs and 11 gray wolves (Additional file 1: Table S1) using GATK (v. 3.4-46; [133]). Using the GATK VQSR procedure, we identified a high quality variant set such that 99% of positions on the Illumina canine HD array were retained. VQSR filtration was performed separately for the autosomes + chrX pseudoautosomal region (PAR) and the non-PAR region. SNPs within 5 bp of an indel identified by GATK were also removed. We further excluded sites with missing genotype calls in any sample, triallelic sites, and X-nonPAR positions where any male sample was called as heterozygous. The final SNP set contained 7,657,272 sites.

Using these SNPs, we removed samples that exhibited over 30% relatedness following identity by state (IBS) analysis with PLINK v1.90 (--min 0.05; [135]). Only one sample (*mxb*) was removed from the sample set, a sample known to be related to another Mexican wolf in the dataset. Principal component analyses were completed on the remaining 53 samples (43 dogs and 10 wolves) using smartpca, a component of Eigensoft package version 3.0 [136] after randomly thinning the total SNP set to 500,000 sites using PLINK v.1.90 [135]. Once PCA confirmed clear genetic distinctions between these dogs and wolves, this final sample set was used for subsequent analyses. For visualization of the final sample set used in selection scans, a further ADMIXTURE plot was generated for this filtered set of 53 samples (Fig. 1b). The SNP set was further filtered for the selection scans to remove rare alleles (minor allele frequencies < 3 out of possible 106 alleles or 0.028). Finally, village dog and wolf allele frequencies were calculated separately using VCFtools [137].

### Demographic model and simulations

Simulations of dog and wolf demographic history were performed using msprime v.0.4.0 [138]. For each autosome, 75 independent simulations were performed using independent random seeds and a pedigree-based genetic map [139]. A mutation rate of 4 × 10^−9^ per site per generation with a generation time of 3 years was assumed. The 53 samples were modeled as coming from 10 lineages with population histories adapted from [34, 40] (Additional file 1: Table S3; Additional file 2: Figure S2). The simulation is designed to capture key aspects impacting dog and wolf diversity, rather than a definitive depiction of their demography. Resulting simulated SNP sets were filtered for minor allele frequency and randomly thinned to have the same number of SNPs per chromosome as the real SNP datasets used in *F*_ST_, XP-CLR, and *H*_P_ calculations.

### *F*_ST_ selection scans

Dog and wolf allele counts generated above were used to calculate the fixation index (*F*_ST_) using the Hudson estimator derived in [140] with the following formula: *F*_ST_ = (p_1_ − p_2_) − (p_1_(1 − p_1_)/n_1_−1) − (p_2_(1 − p_2_)/n_2_ − 1))/(p_1_(1 − p_2_) + p_2_(1 − p_1_)) where p_x_ is the allele frequency in population *x*, and n_x_ is the number of individuals in population *x*, with village dogs and wolves treated as separate populations. With this equation, the X chromosome could be included in *F*_ST_ calculations. A custom script [141] calculated the per site *F*_ST_ across the genome for both the real and 75 simulated SNP sets. Due to differences in effective population size and corresponding expected levels of genetic drift, analyses were performed separately for the chromosome X non-pseudoautosomal region (PAR). Ratio of averages for the resulting *F*_ST_ values were calculated in 200 kb sliding windows with 50 kb step sizes, and we required each window to contain at least 10 SNPs. Additionally, we calculated per site *F*_ST_ for each SNP that did not have missing data in any sample.

*F*_ST_ loci filtration was completed differently for the outlier and non-outlier approach. For the outlier *F*_ST_ approach, the windows were *Z*-transformed and only windows with *Z* scores ≥ 5 standard deviations were deemed significant for autosomal and X-PAR loci, and ≥ 3 for the X-NonPAR. Significance thresholds for the non-outlier approach were determined as the 99th percentile from *F*_ST_ score distributions from the simulated genomes. Overlapping windows passing these thresholds were merged.

### Pooled heterozygosity (*H*_P_) and Δ*H*_P_ calculations

Per window, dog allele frequencies were used to calculate pooled heterozygosity (*H*_P_) using the following formula from [6]: 2Σ*n*_MAJ_Σ*n*_MIN_/(Σ*n*_MAJ_ + Σ*n*_MIN_)^2^, where Σ*n*_MAJ_ is the sum of major and Σ*n*_MIN_ minor dog alleles, respectively, for all sites in the window. Significance threshold for window filtration was set as the 0.1th percentile of the *H*_P_ distribution from the simulated genomes. The change in *H*_P_ (or Δ*H*_P_) was calculated as the difference in Δ*H*_P_ with and without the inclusion of the two ancient dog samples (HXH and NGD). Importantly, genotypes in the ancient samples were determined for the sites variable among the modern samples using an approach that accounts for post-mortem ancient DNA damage [34]. The 5-ky-old German dog (CTC) was not included in this analysis due to known wolf admixture [34]. Windows with Δ*H*_P_ greater than the 5th percentile observed genome-wide were removed.

### XP-CLR selection scans

Cross-population comparative likelihood ratio (XP-CLR; [41]) scores were calculated using pooled dog and wolf allele frequencies at sites described above. This analysis requires separate genotype files for each population, and a single SNP file with positions of each SNP and their genetic distance (in Morgans), which were determined through linear extrapolation from the pedigree-based recombination map from [139]. Wolves were set as the reference population, and XP-CLR was run on both the real and simulated SNP sets with a grid size of 2 kb and a window size of 50 kb. Windows that did not return a value (failed) or did not have at least five grids were removed. Average XP-CLR scores from passing grids were calculated in 25 kb windows (step size = 10 kb). Filtration of real windows with averages less than the 99th percentile of averaged simulation scores was performed. Remaining adjacent windows were merged if they were within 50 kb distance (i.e., one sliding window apart).

### Visualization of candidate domestication regions

Forty-six additional canines (e.g., dog breeds, jackals, coyotes; Additional file 1: Table S1) were genotyped at candidate loci identified in this study, as well as those from [5, 8, 29] using autosomal SNPs previously called in [34]. SNPs within CDRs of interest were extracted from the SNP dataset using the PLINK make-bed tool with no missing data filter. Per sample, each SNP was classified as 0/0, 0/1, or 1/1 at all loci (1 representing the non-reference allele), and this genotype data was stored in Eigenstrat genotype files, which were generated per window using convertf (Eigensoft package; [136]). A custom script [141] then converted the Eigenstrat genotype files into matrices for visualization using matrix2png [142].

### Gene enrichment and variant annotation

Coordinates and annotations of dog gene models were obtained from Ensembl ([143, 144], respectively), and a non-redundant annotation set was determined. The sequence of each Ensembl protein was BLASTed against the NCBI non-redundant database (blastp -outfmt 5 -evalue 1e-3 -word_size 3 -show_gis -max_hsps_per_subject 20 -num_threads 5 -max_target_seqs 20) and all blastp outputs were processed through BLAST2GO [74] with the following parameters: minimum annotation cut-off of 55, GO weight equal to 5, BLASTp cut-off equal to 1e^−6^, HSP-hit cut-off of 0, and a hit filter equal to 55. Of the 19,017 autosomal genes in our non-redundant gene set, 16,927 received BLAST2GO annotations representing a total of 19,958 GO terms. To account effects from differential annotations, we also obtained GO annotations from EMBL-EBI (Ensembl Release 92) for the 19,017 gene models above. Predicted effects of SNP variants were obtained by the processing of the total variant VCF file of all canine samples by variant effect predictor (VEP; [42]).

Positions of predicted domestication regions (XP-CLR or *V*_ST_) were intersected using BEDtools [145] (within a window of 50 kb) with the coordinates of the annotated Ensembl dog gene set to isolate genes within the putatively swept regions, and we defined these as the observed gene set. We performed 1000 randomized shuffles of the loci of interest and, again, identified gene models intersecting within 50 kb, and defined these as the permuted gene sets. Gene enrichment analyses were separately performed on the observed and permuted gene sets using the parent-child model [68] in the topGO R package [69]. Permutation-based *p* values (*p*_perm_) were produced for all GO terms by comparing the observed parent-child test score with the results of the 1000 permutations using the formula *p*_perm_ = (*X*_perm_ + 1)/(*N*+1), where *X*_perm_ is the number of instances where a permutation obtained a parent-child *p* value less than or equal to the observed *p* value, and *N* is the number of permutations (*N* = 1000). One was added to both the numerator and denominator in this equation to avoid adjusted *p* values of 1.0. GO terms with *p*_perm_ values less than 0.05 were further filtered to produce our final enriched GO set. First, terms that were not represented by more than one locus (XP-CLR or *V*_ST_) were removed, as these could have arisen due to clustering of genes belonging to a given gene ontology. Finally, terms were removed if they were represented by only one gene. This occurs when one gene may be spanned by more than one XP-CLR or *V*_ST_ locus. Remaining GO terms are considered the enriched set. This approach was performed separately for BLAST2GO and EMBL-EBI go annotation sets.

### Copy number estimation using QuicK-mer and fastCN

We implemented two copy number estimation pipelines to assess copy number in village dogs and wolves using the depth of sequencing reads. The first, fastCN, is a modified version of existing pipelines that considers multi-mapping reads to calculate copy number within 3 kb windows (Additional file 3: Note 1; [5, 23, 24, 32, 34, 36–38, 66, 145–171]). By considering multi-mapping reads, copy number profiles will be shared among related gene paralogs, making it difficult to identify specific sequences that are potentially variable. The second pipeline we employed, QuicK-mer, a map-free approach based on k-mer counting which can accurately assess copy number in a paralog-sensitive manner (Additional file 3: Note 2; Additional file 4). Both pipelines analyze sequencing read-depth within predefined windows, apply GC-correction and other normalizations, and are able to convert read depth to a copy-number estimate for each window (Additional file 3: Note 3.1). The signal-to-noise ratio (SNR), defined as the mean depth in autosomal control windows divided by the standard deviation, was calculated for each sample (Additional file 3: Note 3.2). The copy number states called by both the QuicK-mer and fastCN pipelines were validated through comparison with aCGH data from [170] (Additional file 3: Note 3.3; Additional file 5). Regions with copy number variation between samples in the aCGH or WGS data were selected for correlation analysis.

### *V*_ST_ selection scans

Treating village dogs and wolves as separate populations, *V*_ST_ values [66] were calculated for genomic windows with evidence of copy number variation. *V*_ST_ values were *Z*-transformed and we identified outlier regions as windows exhibiting at least a 1.5 copy number range across all samples, and ZV_ST_ scores greater than 5 on the autosomes and the X-PAR, or greater than 3 in the X-nonPAR. Prior to analysis, estimated copy numbers for male samples on the non-PAR region of the X were doubled. Outlier regions spanning more than one window were then classified as copy number outlier regions (Additional file 1: Table S7). A similar analysis was performed for the unplaced chromosomal contigs in the CanFam3.1 assembly (Additional file 1: Table S11). See Additional file 3: Note 3.4 for additional methods and details.

### Amylase structural variant analysis

We estimated copy number using short-read sequencing data from each canine listed in Additional file 1: Table S1. Copy number estimates for the *AMY2B* gene using fastCN were based on a single window located at chrUn_AAEX03020568: 4873-8379. See Supplementary Methods: Note 3.5.1 (Additional file 3) for further methods and results. Digital droplet PCR (ddPCR) primers were designed targeting overlapping 1.9 and 2.0 Mb duplications, the *AMY2B* gene and a copy number control region (chr18: 27,529,623-27,535,395) found to have a copy number of two in all sampled canines by QuicK-mer and fastCN. Copy number for each target was determined from ddPCR results from a single replication for 30 village dogs, 3 New Guinea singing dogs, and 5 breed dogs (Additional file 1: Table S12), and averaged from two replicates for 48 breed dogs (Additional file 1: Table S13). For more details on primer design, methods, and results for the characterization of the *AMY2B* locus, see Additional file 3: Note 3.5.

## Additional files

Additional file 1: **Table S1.** Description and accession numbers for canine genomes processed in this study. Indications for whether or not a sample was used in the ADMIXTURE analysis, selection scans (*F*_ST_, XP-CLR, or *V*_ST_) are provided. **Table S2.** Coordinates and annotations of outlier *F*_ST_ loci identified through the empirical approach, including intersecting Axelsson, Cagan and Blass, and Freedman loci [5, 8, 29]. **Table S3.** Parameters incorporated into demographic model for neutral evolution in village dog and wolf populations. **Table S4.** Coordinates and annotations of outlier *F*_ST_ loci identified through the simulation informed (non-empirical) approach, including genes as well as intersecting Axelsson, Cagan and Blass, and Freedman loci [5, 8, 29]. **Table S5.** Coordinates and annotations of outlier XP-CLR candidate domestication regions identified through simulation approach, including intersecting genes as well as intersecting Axelsson, Cagan and Blass, and Freedman loci [5, 8, 29]. **Table S6.** Predicted SNP effects (per variant effect predictor [42]) for sites in XP-CLR candidate domestication regions. **Table S7.** Coordinates of *V*_ST_ copy number outliers on autosomes and chromosome X. **Table S8.** Gene enrichment results for XP-CLR candidate domestication regions following *p* value adjustment (BLAST2GO). **Table S9.** Gene enrichment for XP-CLR candidate domestication regions following *p* value adjustment (EMBL-EBI). **Table S10.** Gene enrichment results for *V*_ST_ copy number outliers following *p* value adjustment. **Table S11.** Coordinates of *V*_ST_ copy number outliers on chromosome unknown (chrUn). **Table S12.** ddPCR results from 30 village, 3 New Guinea singing, and 5 breed dog samples of *AMY2B* segmental duplications. **Table S13.** ddPCR results from 48 breed dog samples of *AMY2B* segmental duplications. (XLSX 328 kb)

Additional file 2: **Figure S1. ***Z*-transformed *F*_ST_ scores for Cagan and Blass locus. **Figure S2.** Demographic model for village dog and wolf populations used in neutral simulations. **Figure S3.** Filtration pipeline implemented for *F*_ST_ and XP-CLR windows. **Figure S4.** Distribution of selection scan statistics for real and simulated *F*_ST_ windows. **Figure S5.** Filtration pipeline implemented for Axelsson, Cagan and Blass, and Freedman CDRs. **Figure S6.** Distribution of selection scan statistics for real and simulated XP-CLR windows. **Figure S7.** Gene intersect statistics from randomized permutations of XP-CLR gene positions. **Figure S8.** Gene intersect statistics from randomized permutations of *V*_ST_ gene positions. **Figure S9.** Read-depth profiles at the AMY2B locus highlights large-scale structural variant. **Figure S10.** Correlations between the ddPCR and read-depth estimated copy number for AMY2B and associated segmental duplications. **Figure S11.** ddPCR results for the AMY2B gene, 1.9 Mb duplication, and the 2.0 Mb duplication. **Figure S12.** Admixture plot for K 2-5 for the full assayed canines for sample filtration. This includes breed dogs, village dogs, as well as gray wolves. (DOCX 3397 kb)

Additional file 3: Notes 1–3 providing supplementary methods and results of copy number analysis. (DOCX 1850 kb)

Additional file 4: Supplemental QuicK-mer validation figures. (PDF 1024 kb)

Additional file 5 Plots displaying aCGH probe intensity correlations with in silico copy number estimates. (PDF 1916 kb)

## Abbreviations

aCGH: Array comparative genomic hybridization
CDR: Candidate domestication region
chrUn: Chromosome unknown
ddPCR: Droplet digital polymerase chain reaction
GO: Gene ontology
*H*_P_: Pooled heterozygosity
NC: Neural crest
NCC: Neural crest cell
qPCR: Quantitative polymerase chain reaction
SNP: Single-nucleotide polymorphism
XP-CLR: Cross-population composite likelihood ratio
