# Supplementary material for: Comparison of village dog and wolf genomes highlights the role of the neural crest in dog domestication
Source: BMC Biol. 2018 Jun 28;16:64. doi: 10.1186/s12915-018-0535-2 (PMC6022502; doi:10.1186/s12915-018-0535-2)
Supplement: Supplementary file 2 — Figure S1. Z-transformed FST scores for Cagan and Blass locus. Figure S2. Demographic model for village dog and wolf populations used in neutral simulations. Figure S3. Filtration pipeline implemented for FST and XP-CLR windows. Figure S4. Distribution of selection scan statistics for real and simulated FST windows. Figure S5. Filtration pipeline implemented for Axelsson, Cagan and Blass, and Freedman CDRs. Figure S6. Distribution of selection scan statistics for real and simulated XP-CLR windows. Figure S7. Gene intersect statistics from randomized permutations of XP-CLR gene positions. Figure S8. Gene intersect statistics from randomized permutations of VST gene positions. Figure S9. Read-depth profiles at the AMY2B locus highlights large-scale structural variant. Figure S10. Correlations between the ddPCR and read-depth estimated copy number for AMY2B and associated segmental duplications. Figure S11. ddPCR results for the AMY2B gene, 1.9 Mb duplication, and the 2.0 Mb duplication. Figure S12. Admixture plot for K 2-5 for the full assayed canines for sample filtration. This includes breed dogs, village dogs, as well as gray wolves. (DOCX 3397 kb) [file 12915_2018_535_MOESM2_ESM.docx]

Supplementary Information for

Comparison of village dog and wolf genomes highlights the pivotal role of the neural crest in dog domestication

Amanda L. Pendleton^1^, Feichen Shen^1^, Angela M. Taravella^1^, Sarah Emery^1^, Krishna R. Veeramah^2^, Adam R. Boyko^3^, Jeffrey M. Kidd^1,4*^

^1^Department of Human Genetics, University of Michigan, Ann Arbor, MI, 48109 USA.

^2^Department of Ecology and Evolution, Stony Brook University, Stony Brook, NY 11794, USA.

^3^Department of Biomedical Sciences, Cornell University, Ithaca, New York, 14853 USA.

^4^Department of Computational Medicine and Bioinformatics, University of Michigan, Ann Arbor, MI 48109 USA.

* Correspondence should be addressed to J.M.K. at jmkidd@umich.edu

**This file includes:**

Figures S1 – S12


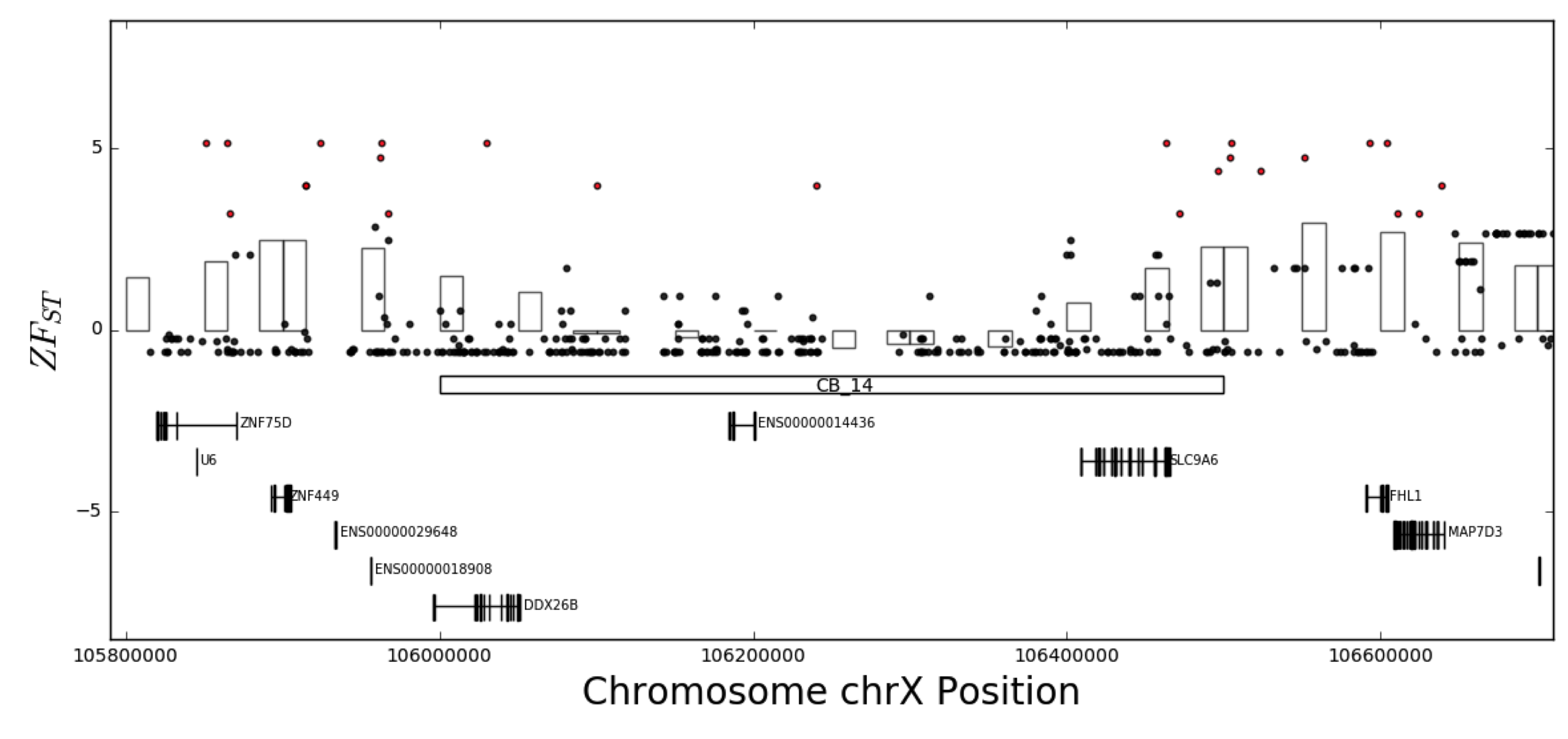


**Figure S1:** Z-transformed F_ST_ scores (ZF_ST_) per site (dots) and window average (bars) surrounding the Cagan and Blass locus #14 (CB_14) on Chromosome X. Red dots indicate significant ZF_ST_ scores. Chromosomal positions are along the X axis. Ensembl gene models are provided below the plot.

**Figure S2:** Demographic history of village dog and wolf populations. Red arrows indicate directionality of gene flow between dog and wolf populations. Estimated split times can be inferred from the timeline beneath the tree. Nodes (T_N_) correspond to events detailed in Additional File 1: Table S3.

**_
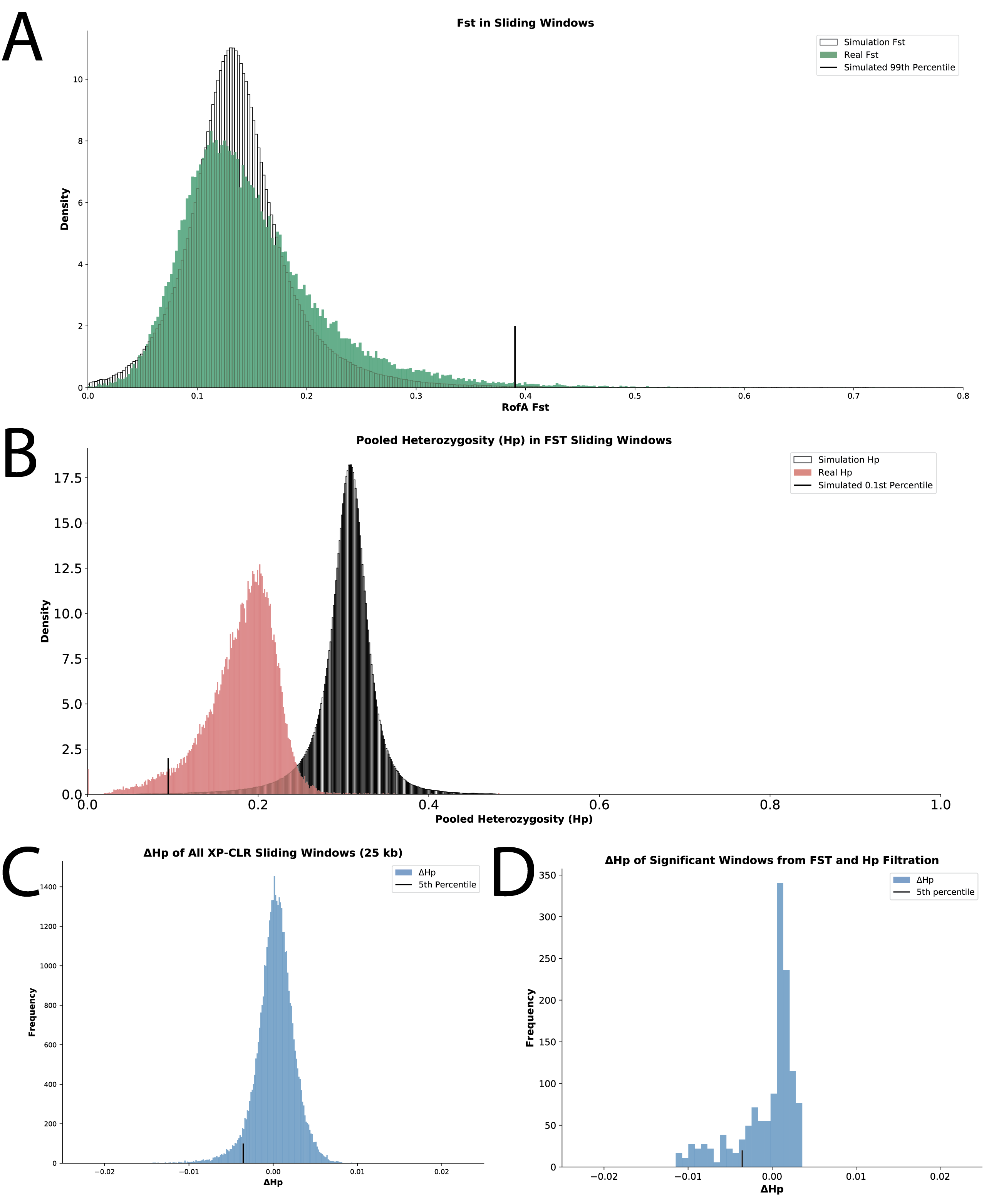
_**

**Figure S3:** (A) Distribution of real (white) versus simulated (green) averaged F_ST_ values in 200 kb sliding windows with step sizes of 50 kb. The 99^th^ percentile is indicated as a black line. (B) Distribution of real (white) versus simulated (red) H_P_ values in 200 kb sliding windows with step sizes of 50 kb. The 0.1^th^ percentile is indicated as a black line. Distribution of per window differences in H_P_ with and without the inclusion of ancient samples (ΔH_P_) is given for (C) all genomic windows and (D) windows that passed F_ST_ and H_P_ simulation-based filtrations.

**Figure S4**: Filtration pipeline used for F_ST_ and XP-CLR windows. Average scores from windows of 200kb (F_ST_) and 25kb (XP-CLR) were (A) required to be greater than the 99^th^ percentile of simulated windows of the same size for the respective selection scan statistic. (B) Windows were further filtered out if the average pooled heterozygosity (H_P_) values were greater than the 0.1th percentile of simulated windows for the same statistic. (C) Next, windows where the difference in H_P_ values (calculated as Hp with and without the inclusion of ancient dogs) was greater than the 5th percentile of genome-wide windows were removed. (D) Finally, windows passing the above steps were merged if they were within one window distance.

**Figure S5:** Results of filtration pipeline (first implemented on this study’s F_ST_ and XP-CLR loci) on the candidate loci of previous studies (see Figure S3). Maximum F_ST_ scores from within the boundaries defined by Axelsson et al. 2013, Cagan and Blass 2016, and Freedman et al. 2016 were (A) required to be greater than the 99^th^ percentile of F_ST_ values from simulated windows of the same size. (B) Windows were further filtered out if the average pooled heterozygosity (H_P_) values were greater than the 0.1th percentile of simulated windows. (C) Next, windows where the difference in H_P_ values (calculated as Hp with and without the inclusion of ancient dogs) was greater than the 5th percentile of genome-wide windows were removed. (D) Finally, windows passing the above steps were merged if they were within one window distance. Below, the number of passing windows that intersect with simulation-based F_ST_ and XP-CLR windows generated from this study on village dogs is provided per source.

**
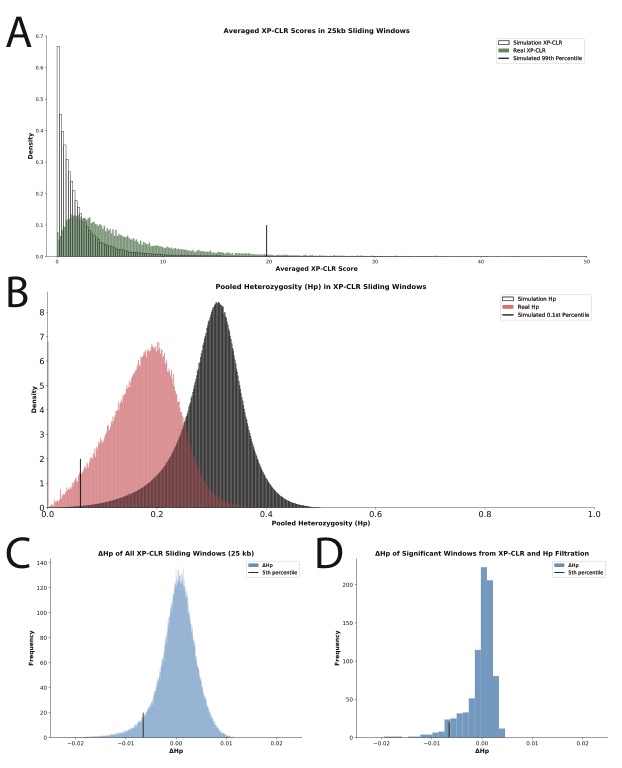
**

**Figure S6:** (A) Distribution of real (white) versus simulated (green) averaged XP-CLR values in 25 kb sliding windows with step sizes of 10 kb. The 99^th^ percentile is indicated as a black line. (B) Distribution of real (white) versus simulated (red) H_P_ values in 25 kb sliding windows with step sizes of 10 kb. The 0.1^th^ percentile is indicated as a black line. Distribution of per window differences in H_P_ with and without the inclusion of ancient samples (ΔH_P_) is given for (C) all genomic windows and (D) windows that passed XP-CLR and H_P_ simulation-based filtrations.

**Figure S7:** Following 1,000 randomized shuffling (permutations) of gene positions within XP-CLR loci, distributions of (A) the number of permuted windows that intersects within 50kb of at least one gene, (B) the total number of genes within 50kb of permuted windows, and (C) the average size of genes intersecting within 50kb of permuted windows are plotted above. The number observed in the real data (*i.e.* unshuffled) is indicated with a black bar (‘Obs.’).

**Figure S8:** Following 1000 randomized shuffling (permutations) of gene positions within V_ST_ candidate domestication regions (VCDRs), distributions of (A) the number of permuted windows that intersects within 50kb of at least one gene, (B) the total number of genes within 50kb of permuted windows, and (C) the average size of genes intersecting within 50kb of permuted windows are plotted above. The number observed in the real data (*i.e.* unshuffled) is indicated with a black bar (‘Obs.’).


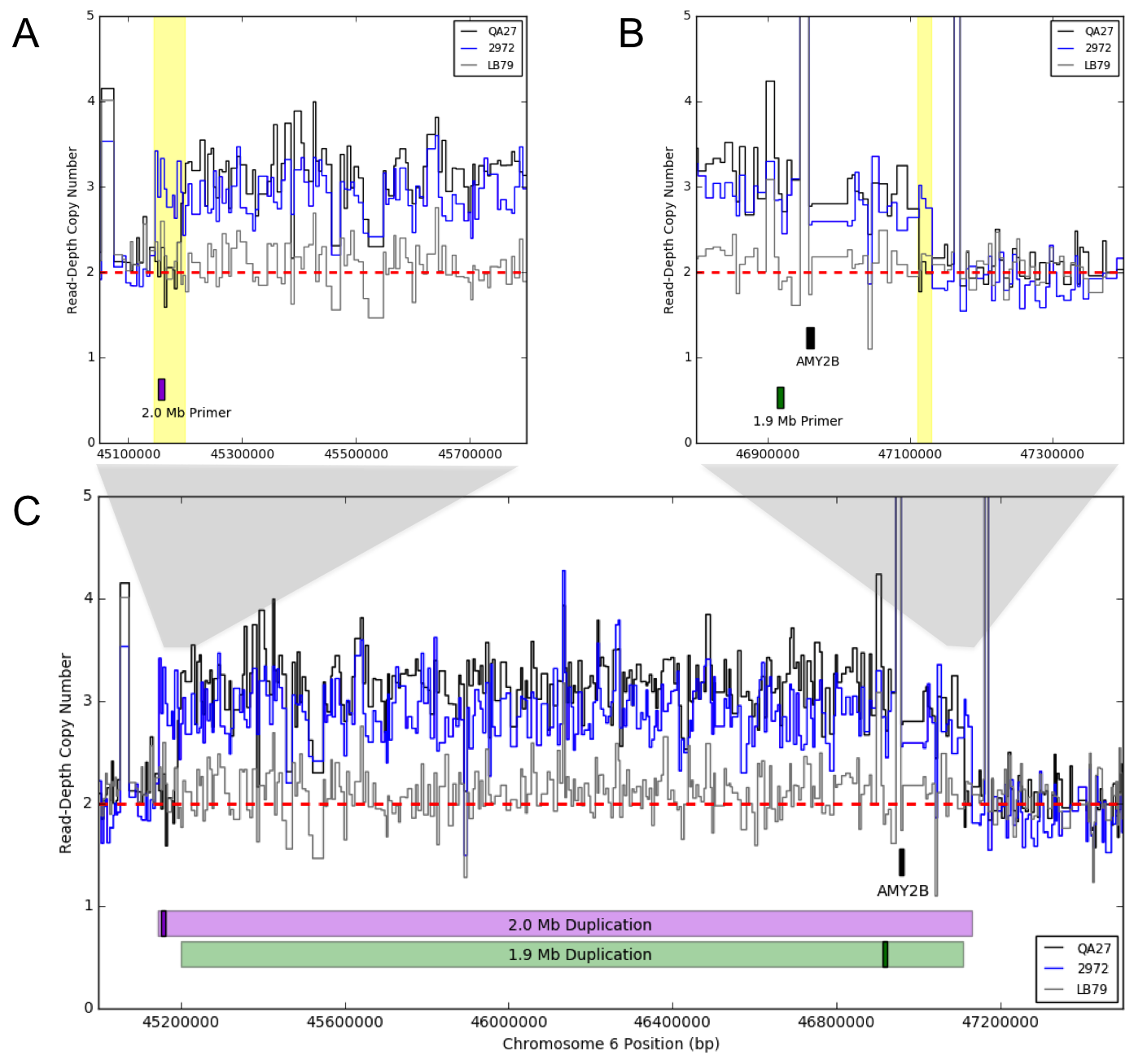


**Figure S9:** Read-depth based CN estimations for the (A) full chromosome 6 region of interest, as well as the (B) proximal and (C) distal margins of large-scale duplications surrounding the *AMY2B* locus. Estimates for three dogs with differential duplication genotypes are displayed in this figure including those with either the 1.9Mb (QA27; black) or 2.0Mb (2972; blue) duplication, compared to a dog without a large-scale duplication (LB79; gray). Relative positions of the 1.9Mb (green bar) and 2.0Mb (purple bar) duplications are indicated along with their respective primers (darker boxes within duplications). Extensions of the 2.0Mb duplication relative to the 1.9Mb duplication are highlighted in yellow.


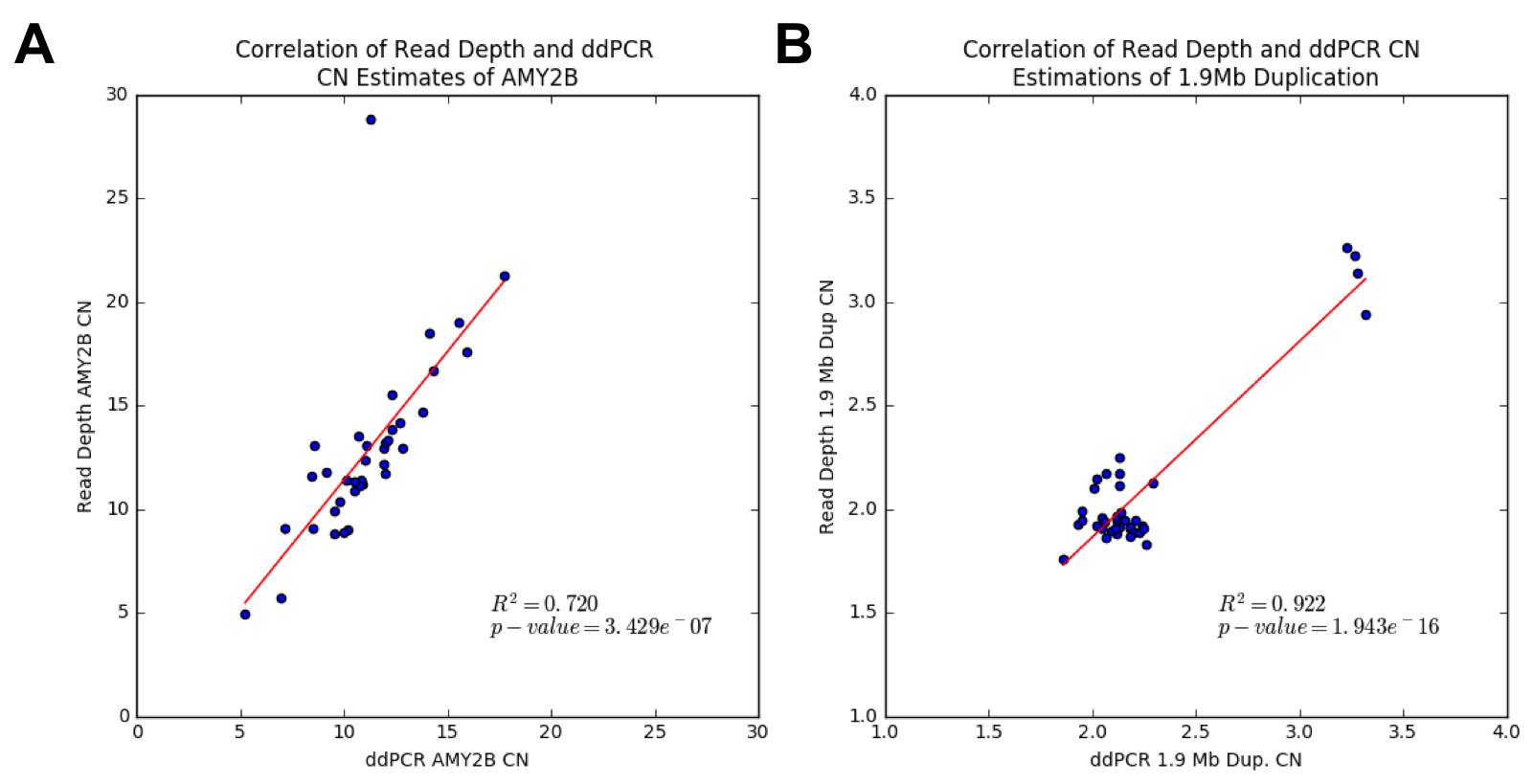


**Figure S10:** Correlation of ddPCR and read-based copy number estimates are provided for (A) the *AMY2B* gene and (B) its encompassing 1.9Mb duplication. The r-square and p-values for the respective correlations are provided on the plots.

**
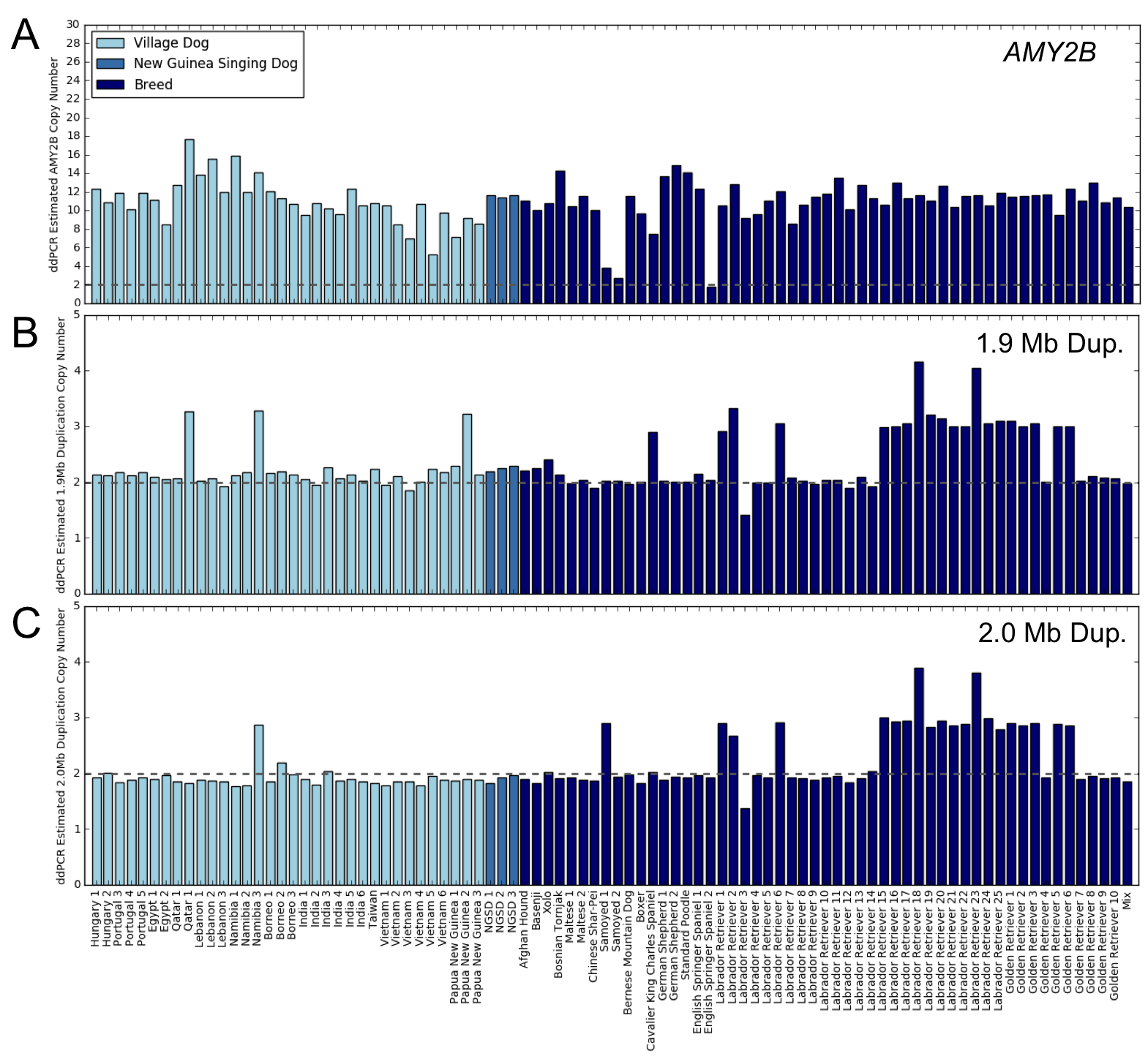
**

**Figure S11:** ddPCR copy number estimates are provided for (A) AMY2B gene, (B) the 1.9 Mb and (C) 2.0 Mb duplications. Village dogs are in light blue, New Guinea Singing Dogs (NGSD) are in medium blue, while breed dogs are in dark blue.

**Figure S12:** Admixture plot for K 2-5 for the full assayed canines for sample filtration. This includes breed dogs, village dogs, as well as gray wolves.
