# Supplementary material for: Comparison of village dog and wolf genomes highlights the role of the neural crest in dog domestication
Source: BMC Biol. 2018 Jun 28;16:64. doi: 10.1186/s12915-018-0535-2 (PMC6022502; doi:10.1186/s12915-018-0535-2)
Supplement: Supplementary file 3 — Notes 1–3 providing supplementary methods and results of copy number analysis. (DOCX 1850 kb) [file 12915_2018_535_MOESM3_ESM.docx]

# Supplementary Note 1 - Copy number estimation with fastCN

### 1.1 Introduction

Multiple approaches that utilize read depth to identify regions of copy number variation have been developed. One successful set of approaches utilize the mrFAST and mrsFAST aligners, tools which efficiently return all matching locations for short sequencing reads within a specified edit distance. These tools have been used to analyze CNV patterns in multiple studies of humans and non-human primates [1-4]. However, this estimation required separate steps including mapping, BAM file sorting based on location, and read pileup followed by GC corrections, requiring the storage and manipulation of several large files. Since the total time for disk I/O and the use of multiple intermediate files is a serious bottleneck for large scale analyses, we developed fastCN to efficiently estimate genome copy number from short read data. This program utilizes the data output from the short read mapper mrsFAST [5], and reports per-bp read depth in an efficient compressed binary format. The fastCN software package is available on the Kidd Lab GitHub [6][.](https://github.com/KiddLab/fastCN)

### 1.2 Implementation and optimization

The fastCN core pipeline consists of two major applications. The first, [GC_control_gen](https://github.com/KiddLab/fastCN/blob/master/GC_control_gen.cc), generates a control region file for the next stage of the pipeline based on (1) the reference genome and user supplied files indicating (2) regions of the genome assumed to not be copy number variable and (3) regions of the genome which have been masked prior to read mapping. To avoid excessive depth pile ups due to repetitive regions, we utilize a version of the genome reference where all elements defined by RepeatMasker, elements defined by tandem repeat finder (TRF), and 50-mers with at least 20 genome matches within an edit distance of two are masked to ‘N’ prior to short read mapping. To avoid the shadow effect of mapping against a masked genome, the coordinates of the masked segments are extended by the length of the utilized reads. For compatibility with previous work, we utilize a read length of 36 bp, and divide longer Illumina reads into disjoint 36 bp long sequences.

The control region file is encoded as a 32-bit float per base pair in a pure binary representation, and the value for each float corresponds to the local GC content at that base pair. The window length for GC content calculation is defined by the user (typically 400 bp), and GC content values are assigned to the centers of each sliding window. Signed values are given for each base-pair with expected values between -1.0 and 1, where a negative bit value indicates a base pair that should not be used as a control for normalization. Masked genomic regions are identified by a value of negative infinity and are omitted from processing. This encoding scheme allows rapid access during the subsequent normalization stage. For each reference assembly, the above step should be executed once.

The second application, [SAM_GC_correction](https://github.com/KiddLab/fastCN/blob/master/SAM_GC_correction.cc), processes the data output from the mrsFAST mapper (an unsorted SAM file format). As such, a memory space proportional to the size of the haploid reference genome is required for this random access. Once the mapping input is processed, GC normalization ensues with the aid of the binary file from the previous step. GC normalization utilizes a multiplicative correction factor determined by lowess fitting, as utilized in QuicK-mer (see Supplementary Note 2). The end result is corrected depth preserved with half floating point precision, which contains sufficient dynamic range and precision while significantly saving disk space. Depths at regions masked out in the reference are assigned a fixed depth value of -1.0. The resulting binary normalized depth files are subsequently compressed using gzip. Mean or median depth values in predetermined windows can then be efficiently calculated from these files, and converted to estimates of genome copy number.

### 1.3 Performance

The fastCN pipeline achieves excellent performance. The core pipeline consumes negligible additional time compared to mrsFAST mapping. The control region files can be constructed under 3 minutes for a typical 3Gb mammalian genome. Experimental validation of results based on array CGH and ddPCR are described in Supplementary Note 3.3 (below).

### 1.4 Utility applications

A utility application is included for the user to convert between half float and single precision float point. Please refer to readme file from the fastCN software package on GitHub for additional instructions.

# Supplementary Note 2 - Development of QuicK-mer, a paralog-sensitive copy number estimation pipeline

## 2.1 Introduction

Detecting copy number variation (CNV) from high-throughput sequencing data is a prevalent and important problem in the research of genome evolution, population genetics, and disease. Several methodologies have been developed that make use of distinct features of the data including read depth, read-pair mappings, split-reads, sequence assembly, and combinations of multiple signals [7]. However, current methods are not without limitations. For example, read depth approaches rely upon mapping to a reference genome assembly and are typically unable to isolate variation among duplicated sequences present in multiple locations in the reference. Specialized approaches have been developed to address this limitation, including tabulating depth at paralog-specific nucleotide positions using customized tools [3, 4, 8]. Although effective, these approaches require re-analysis of existing data using specialized mapping programs or extensive downstream processing, both of which can add considerable time to the analysis. Additionally, previous approaches require trimming and/or partitioning of sequence reads into subsequences of a specified length and therefore do not make full use of the available sequence data. Here, we present QuicK-mer, a rapid and paralog-sensitive CNV estimation pipeline that efficiently produces copy number estimates from FASTQ or BAM input files in less than 6 hours. Our approach is mapping-free and relies upon efficient tabulation of read depth at predefined sets of informative k-mers.

## 2.2 Implementation

To achieve efficient and paralog-specific CNV estimation, we focused on counting specific k-mer sequences rather than aligning reads to a reference, an approach that has also been proposed for analysis of RNA-Seq data [9-11]. The QuicK-mer pipeline is designed to utilize the existing Jellyfish k-mer counting application [5]. Accepting both FASTQ and BAM files as input, QuicK-mer is designed for sequences generated by the Illumina platform.

To speed up CNV estimation, QuicK-mer requires two major pre-processing steps for each genome assembly. These two steps are essential to generate binary files for efficient access within the core CNV estimation pipeline. These two binary files are described further in the next section and are used to estimate the CNV in each sample. For detailed operation, refer to the QuicK-mer operation manual in the software package [6, 12].

### 2.2.1 Preprocessing steps

#### 2.2.1.1 Defining a catalog of unique K-mers

Defining a catalog of unique k-mers requires seven individual steps. In practice, we utilize a size of k=30 for consistency with previous studies [3, 4]. An example of the command lines for each of the following steps can be found in the QuicK-mer User Manual v1.0 Section 9 [12].

1. List all unique 30-mers: All 30-mers in the reference genome are enumerated with Jellyfish by setting k-mer size equal to 30 and using the reference genome FASTA sequence as the input. A k-mer and its reverse complement are considered equal (Jellyfish option –C). The 30-mers with a count of 1 are exported into text format.

2. Determine unique 30-mer locations: Unique 30-mers are mapped to the genome reference using mrsFAST [5] with an edit distance setting of 0. This step serves to map the location of each unique 30-mer and is used in the following steps for region overlapping and exclusion.

3. Enumerate highly repetitive 15-mers: The same procedure for Step 1 is repeated for the reference assembly except now k is set equal to 15 and all 15-mers with counts ≥ 1,000 are exported.

4. Determine repetitive 15-mer locations and filter 30-mers: Step 2 is repeated with the 15-mers determined in Step 3. Here, each k-mer will have multiple genome locations. Finally, locations of the 15 and 30-mers are merged together, and all 30-mers (from Step 2) that overlap with the high frequency 15-mer track are removed from subsequent analyses.

5. Remove highly similar 30-mers: The 30-mers that pass Step 4 are mapped onto the reference genome using mrsFAST with an edit distance of 2. All 30-mers with ≥ 100 mapped positions are removed. Note that mrsFAST only considers substitutions.

6. Considering indels, remove highly similar 30-mers: The k-mers that pass Step 5 are mapped again using mrFAST with an edit distance of 2. All 30-mers with ≥ 100 mapped positions are removed. The mrsFAST search is performed prior to mrFAST due to the speed advantage of mrsFAST only considering mismatches. Steps 5 and 6 serve to reduce the chances of matching k-mers with sequencing errors into unintended locations.

7. Combine final k-mer catalog: The final list of highly unique 30-mers is sorted based on chromosome location and outputted in BED format. This output file will then be used by QuicK-mer and for the generation of required auxiliary files.


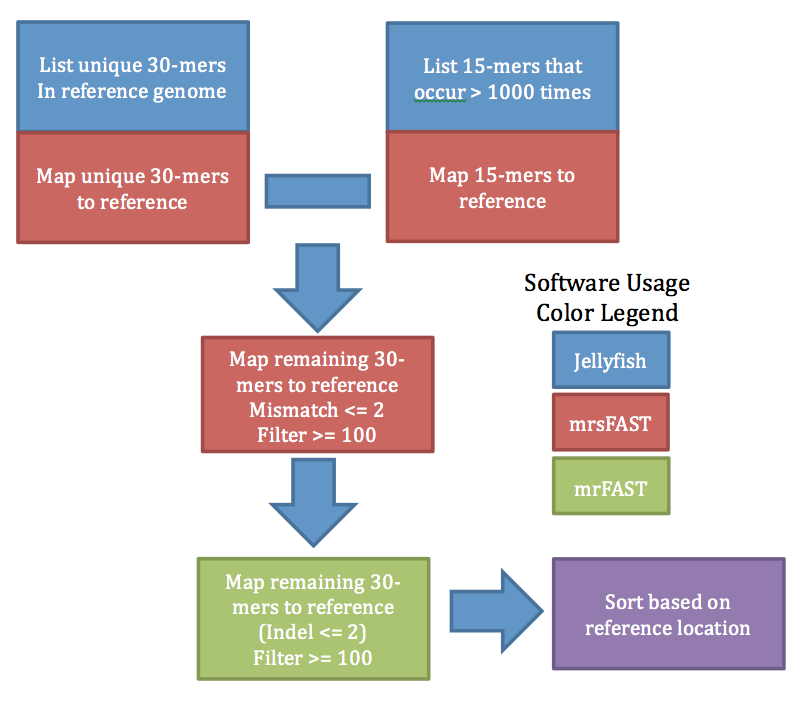


**Figure S2.2.1** Diagram depicting QuicK-mer workflow for 30-mer generation from a genome reference assembly.

####

#### 2.2.1.2 Defining genome control regions

The resulting file is encoded in a binary file for convenient access during the GC correction and copy number normalization step.

####

#### 2.2.1.3 Generating local GC content

To counter the amplification bias during library preparation and flow cell bridge amplification, a moving window based local GC content is calculated for a reference genome assembly. For each K-mer, this window is taken by extending from the central base pair by half of the window length. In our study, the GC window is set to a value of 400bp, which is typical for a WGS library. In the same manner as the previous step, the values are stored in binary file for rapid access.

### 2.2.2 Core pipelines

#### 2.2.2.1 Depth estimation and GC correction

The QuicK-mer core program is written in C++ and Object Pascal and wrapped with Python for control flow. The control flow consists of calling Jellyfish-2 [13] for building the 30-mer hash library followed by the k-mer query step. At the beginning of the query step, two axillary binary files are preloaded and memory space for count values is allocated. QuicK-mer then interrogates the Jellyfish hash library with the sorted 30-mer list, storing each raw count value in memory. The core program verifies each 30-mer’s status as a normalization control and, if indicated, the 400 bp GC-content value is fetched from the associated binary file and incorporated into the GC bias curve. Once the process is finished, the core program builds the GC curve based on the average counts obtained from each GC percentage bin and uses the lowess smoothing algorithm to generate a correction curve. The targeted average depth is calculated using a weighted average based on GC content of 25~75%. A 0.3x minimum and 3x maximum correction factor is also enforced to reduce over-correcting extreme GC regions due to a lack of representative k-mers. The GC bias curve is output in a text format and, along with correction curve, is represented in a PNG image (example in Figure S2.2.2.1). Lastly, the correction factor is applied to each k-mer count value based on its GC content and the resulting GC-corrected k-mer counts are outputted in binary format.


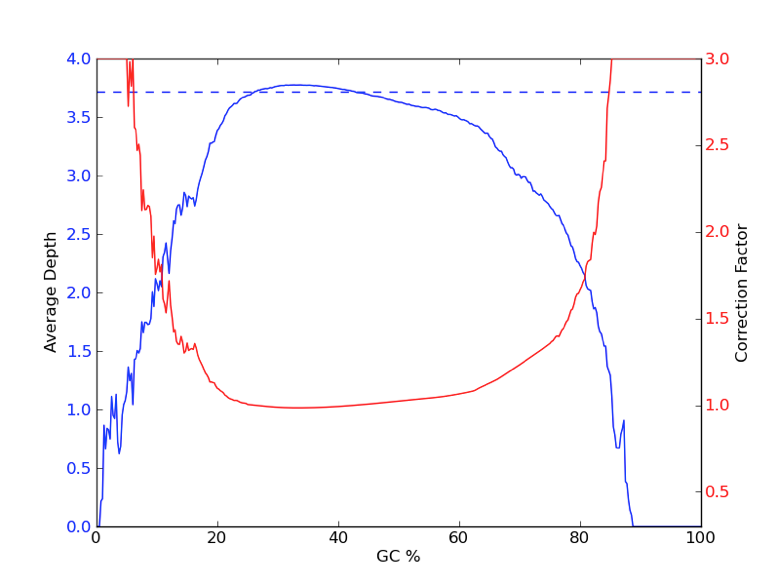


**Figure S2.2.2.1** GC Correction and Bias: The majority of sequencing coverage bias is related to local GC content. The blue curve indicates the average depth for the 30-mers with the same GC content in 400bp surrounding the center of each k-mer location, rounded in steps of 0.25%. The red curve is the lowess smoothed correction factor, targeted for the average depth indicated by the dashed line. A 3x max correction value is enforced. The GC curve represents QuicK-mer run from WGS experiment SRX734522.

Due to different GC biases within sequencing libraries and across flow cell lanes, the user is encouraged to apply QuicK-mer GC normalization separately for each sequencing lane. Resulting GC-corrected k-mer counts can then be merged together for each sample using the CorDepthCombine command.

#### 2.2.2.2 Normalization and CNV estimation

Another program in the QuicK-mer package (kmer2window) converts counts to copy number estimates. The normalization program loads the same binary control region file (see Supplementary Note 2.2.1.2) then, using the corrected depth for the control 30-mers, calculates a scaling factor based on an assumed copy number of two for these regions. Normalization is performed on windows of equal number of k-mers (default = 500 k-mers per window, but is adjustable by the user). The median k-mer count for each window is used for the normalization, and only windows where all k-mers are in the defined control intervals are used in subsequent steps. The resulting normalization is then applied to all windows.

## 2.3 Results

### 2.3.1 Performance

To assess the efficiency of QuicK-mer, we randomly sampled subsets of reads from the human genome sequence for sample HG02799, which was sequenced to a depth of 17x. The selected fractions were individually analyzed using 35GB memory and 4 cores during library construction and 2 cores during querying on an empty compute node with 4 Xeon E7 4850 2GHz processors and 1TB of total memory. Wall clock-time statistics indicate a constant time cost for the querying step once the average sequencing depth exceeds 1x. The nature of counting predefined k-mers also means the memory usage is unlikely to be affected by the sequencing depth. The library building time is linearly correlated with the input read counts.


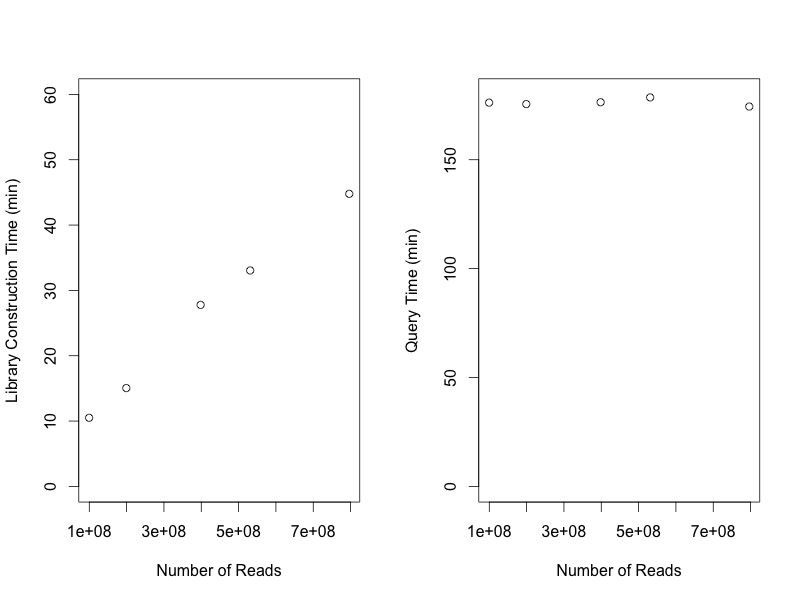


**Figure S2.3.1.1** Wall time statistics from random read sampling of a HG02799 Illumina library.

### 2.3.2 Validation with 1000 genome data

For comparison, we reanalyzed data from the 1000 Genome Project and other sources using QuicK-mer and compared the estimated copy number profiles with supplementary data from the [3] study. The dataset was downloaded from 1000 Genome Project Pilot, Phase 1, and Phase 3 studies [14-16]. Sequencing files were individually run through the QuicK-mer pipeline and GC corrections were performed for each sequencing lane. Corrected data is combined and normalized into copy number estimates. Table S3.3.2.1 contains the details of samples used to assess the accuracy of QuicK-mer in known CNV regions.

**Table S2.3.2.1** Samples used for QuicK-mer validation. The mean depth is calculated based on the median depth obtained from windows of 500 30-mers that fully overlap a defined control region. For sample NA19240, the SRA accession identifiers are provided.

| **Sample Name** | **Data Source** | **Mean 30-mer Depth in Control** |
| --- | --- | --- |
| NA12156 | 1000 Genome Phase 3 | 4.40 |
| NA12878 | 1000 Genome Phase 1, Pilot 1/2 | 7.23 |
| NA18507 | [17] | 23.05 |
| NA18508 | [17] | 7.30 |
| NA18517 | 1000 Genome Phase 3 | 3.82 |
| NA18555 | 1000 Genome Phase 3 | 4.09 |
| NA18956 | 1000 Genome Phase 3 | 3.63 |
| NA19129 | 1000 Genome Phase 1, Pilot 1/2 | 0.87 |
| NA19240 | SRX574476, SRX582073  [18] | 13.26 |

We evaluated the genome regions depicted in S52 and S60 – S71 of [3]. QuicK-mer accurately estimated copy number for many highly paralogous gene families, such as the *UGT2* gene family (Figure S3.3.2.1), for each sampled human genome. Other regions in which QuicK-mer CNV estimations are consistent with the original study, further demonstrating the accuracy of QuicK-mer in detecting copy number of unique paralogs, can be found in Additional File 4.


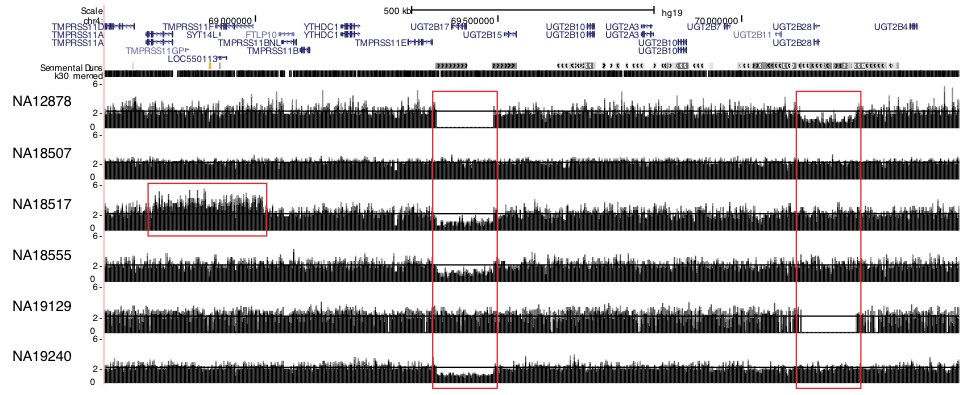


**Figure S2.3.2.1** Diverse and paralog-specific CNV detected by QuicK-mer at the UGT2 family locus for numerous gene models (top track) at chr4q13.2. Red boxes indicate regions of detected CNV. UGT2B17 is hemizygously deleted in NA19240, NA18555 and NA18517. TMPRSS11F and SYT14L are duplicated in NA18517, resulting in a copy number of 3. This figure corresponds to the region shown in Figure S65 in [3]. The k30_merged track indicates the locations with unique 30-mers.

We further validate the performance using aCGH on a dog reference assembly in Supplementary Note 3.3.

## 2.4 Summary

QuicK-mer is a map-free approach that scales well, requires minimal additional processing, and represents a unified pipeline for efficiently estimating paralog-specific copy number from Illumina WGS data. This map-free approach removes the computational burden required to reprocess data using specialized mapping tools such as mrsFAST. The QuicK-mer pipeline can also be easily extended. For example, instead of solely examining k-mers unique to paralogs, CNV of broad sequence classes, such as mammalian sex chromosome ampliconic sequences [19], can be assessed using a catalog of k-mers present in all copies of particular amplicon classes [20]. Additionally, the k-mer counting approach is easily scalable, and extensions to systems that use a map-reduce framework (such as Hadoop) may permit more efficient analyses of tens of thousands of whole genomes.

## 2.5 Availability

The Quick-mer pipeline is open source and available on GitHub at[12]. Further documentation and a user manual are available in the software package. Pre-computed unique 30-mers for human (hg19), mouse (mm10), chimpanzee (panTro4), and dog (CanFam3.1) genomes can be downloaded from [21].

# Supplementary Note 3 - Estimation of copy number and copy number selection scans

## 3.1 Copy number estimation using Illumina sequencing data

Copy number was estimated using Illumina whole genome sequencing data with both the fastCN and QuicK-mer methods described above (Supplementary Notes 2 and 3). Input sequencing data for both approaches was derived from BAM files with duplicated reads removed. Non-CNV autosomal control regions for depth normalization were predefined for the CanFam3.1 reference by excluding regions previously reported to be duplicated or copy number variable [22-25]. Copy number estimates were created in windows of 3,000 unmasked bp (fastCN) or 3,000 unique k-mers (QuicK-mer) for the autosomes and chromosome X. Unplaced contigs were merged into one ‘chrUn’ for copy number estimation according to details described in Supplementary Note 3.4.5.

### 3.1.1 QuicK-mer CN estimation

The canine reference assembly was divided into consecutive windows that each have 3,000 k-mers (Supplementary Note 3.2.1.1). Since k-mer locations are not uniform or consecutive, the actual genomic span (or length) of each window varies depending on the local sequence complexity. Window definition is constructed using an utility application in the QuicK-mer pipeline (Supplementary Note 3). Copy number estimates were calculated using the kmer2window program, which requires the 3,000 k-mer windows (referenced above), as well as the normalized binary files for each sample (available for download at [21].

### 3.1.2 fastCN CN estimation

Similar to QuicK-mer CNV estimation, we divided the canine genome into consecutive 3kb windows, with the exclusion of masked regions defined in the fastCN pipeline. The depth for each window is first assigned the mean normalized depth of all intersecting unmasked base pairs. This value is then scaled to copy number estimate per window by dividing the average depth in all control windows assumed to be copy number of two.

## 3.2 Comparison of noise across samples

The signal-to-noise ratio (SNR), defined as the mean depth in autosomal control windows divided by the standard deviation, was calculated for the 53 dogs and wolves that were processed through the SNP-based F_ST_ pipeline. Because the wolf samples were typically sequenced to a higher depth than the village dog samples, wolves display larger SNR than dogs (Figure S3.2.1a and b). However, many village dogs with lower average sequence depth (~4-10x) exhibit comparable SNRs with wolves that have higher depths. The correlation of noise in control regions between both pipelines indicates a consistent noise originating from the sequencing data (Figure S3.2.1c). Results from a boxer breed dog (box), which is used in subsequent QuicK-mer and fastCN validations (Supplementary Note 3.3), is also included in these plots. The SNR of this sample indicates that the boxer sequencing data is unusually noisy, an observation accounted for in later analysis.

A B
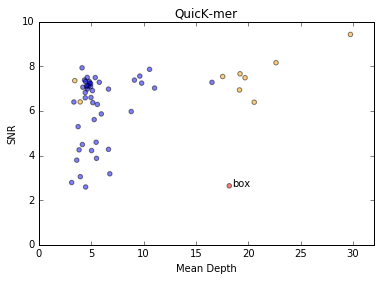

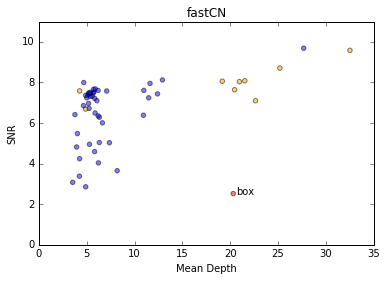


C


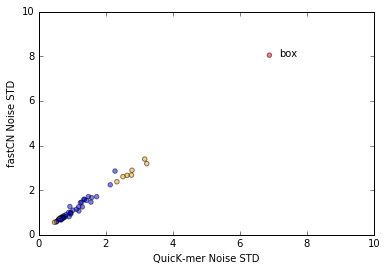


**Figure S3.2.1** Signal to noise ratio (SNR) values based on (A) QuicK-mer and (B) fastCN (upper right) analyses are plotted against genome sequence depth for all samples used in the study. The SNR values were obtained from 3kb control region windows. (C) Correlation of noise standard deviations (STD) between QuicK-mer and fastCN. Village dogs are in blue and wolves in orange, while the aCGH reference sample (box) is red.

## 3.3 Comparison with CGH array data

Comparative genomic hybridization array (aCGH) data from a previous study [26] was downloaded from Gene Expression Omnibus under the accession number GSE58195 (Figure S3.3.1). This study utilized a NimbleGen aCGH chip that contained 598,733 probes with average spacing of oligonucleotide probes at 157 bp, and tested the comparative binding of DNA to estimate CNV between dogs and wolves at sites incorporated into the aCGH design [26].

**Table S3.3.1** Sample information for aCGH data deposited under GEO accession number GSE58195 from [26]. The sample identifiers used in this study, sample descriptions, GEO accession for the aCGH data, SRA data accession for whole genome sequence, and sample sex is provided.

| **Sample ID** | **Sample Description** | **aCGH Data Accession ID** | **SRA Data Accession ID(s)** | **Sex** |
| --- | --- | --- | --- | --- |
| chw | Chinese Wolf | GSM1402955 | SRX1137190, SRX1137189, SRX1137188 | Female |
| glw | Great Lakes Wolf | GSM1402952 | SRX655630, SRX655629 | Male |
| inw | Indian Wolf | GSM1402956 | SRX655632, SRX655631 | Male |
| irw | Iranian Wolf | GSM1402953 | SRX655634, SRX655633 | Female |
| mxa | Mexican Wolf | GSM1402954 | SRX655637, SRX655636 | Female |
| ptw | Portuguese Wolf | GSM1402949 | SRX655640, SRX655639 | Female |
| ysa | Yellowstone Wolf | GSM1402951 | SRX655648,  SRX655647,  SRX655646 | Female |
| box | Boxer | GSM1402940 | SRX655611, SRX655610 | Female |

In [26], DNA from a Boxer breed dog (box) is used in the aCGH control channel. However, the sequencing data from the same sample shows poor quality due to extremely uneven coverage (Figure S3.1.1). To circumvent the impact of this noisy sample in our aCGH validation, we employed a simple log difference transformation (Equation 3.3.1). Assuming the hybridization for the boxer sample performs equivalently across experiments, this approach effectively cancels out the boxer as the aCGH reference sample and instead directly compares copy number between samples 1 and 2. To make validation based on sequencing depth comparable to relative estimates from array CGH, we employed a *in silico* transformation on the copy number estimates using Equation 3.3.2, where the numerator and denominator are the normalized copy number state in each 3kb window for samples 1 and 2, respectively.

**Equation 3.3.1** ${log}_{2} \frac{Probe Intensity of Sample 1}{Probe Intensity of box}-{log}_{2}\frac{Probe Intensity of Sample 2}{Probe Intensity of box}$

**Equation 3.3.2** ${log}_{2} \frac{Copy Number of Sample 1}{Copy Number of Sample 2}$

To compare the result between Equation 3.3.1 and 3.3.2, we lifted over the aCGH probe location to CanFam3.1 reference coordinates. Next, the values from Equation 3.3.1 for all the probes that intersect with a 3kb fastCN or QuicK-mer window were averaged and then assigned to the window. Figure S3.3.1 illustrates the probe count distribution for 3kb windows, a similar distribution was found for 3,000 k-mer windows, and we observed that QuicK-mer and fastCN had similar distributions. In total, 12,584 and 17,989 3kb windows intersect with at least one aCGH probe for fastCN and QuicK-mer, respectively. We further filtered these windows to only include those containing at least three aCGH probes, thus reducing the window counts to 11,876 and 17,774 for fastCN and QuicK-mer, respectively.


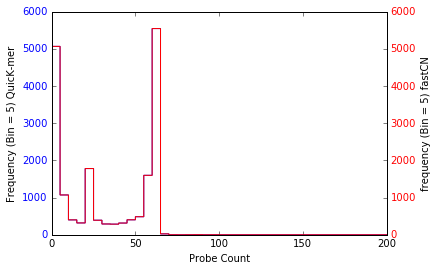


**Figure S3.3.1** Distribution of probe in fastCN and QuicK-mer window

From the previous two steps, each QuicK-mer and fastCN window was assigned two log-ratio values, one from the from the mean of log-ratios from aCGH probes overlapping the window (Equation 3.3.1) and another from the *in silico* copy number estimates (Equation 3.3.2). We filtered windows that contained less than three probes and whose *in silico* vs aCGH log ratio values fell within a circle around the origin on the plot, defined to be x^2^ + y^2^ < R^2^ (Figure S3.3.2). We set the radius R equal to 0.4 for both fastCN and QuicK-mer, which corresponds to 1.4x change in probe intensity or copy number. This filtration step is necessary because linear regression will be skewed toward a cluster of noise which has no meaningful correlation near the plot origin, since the majority of probes in the aCGH are in regions that are not variable between the two samples being compared. The remainder of the data points are used for linear regression. Plots for each pairwise comparison can be found in Additional File 5.
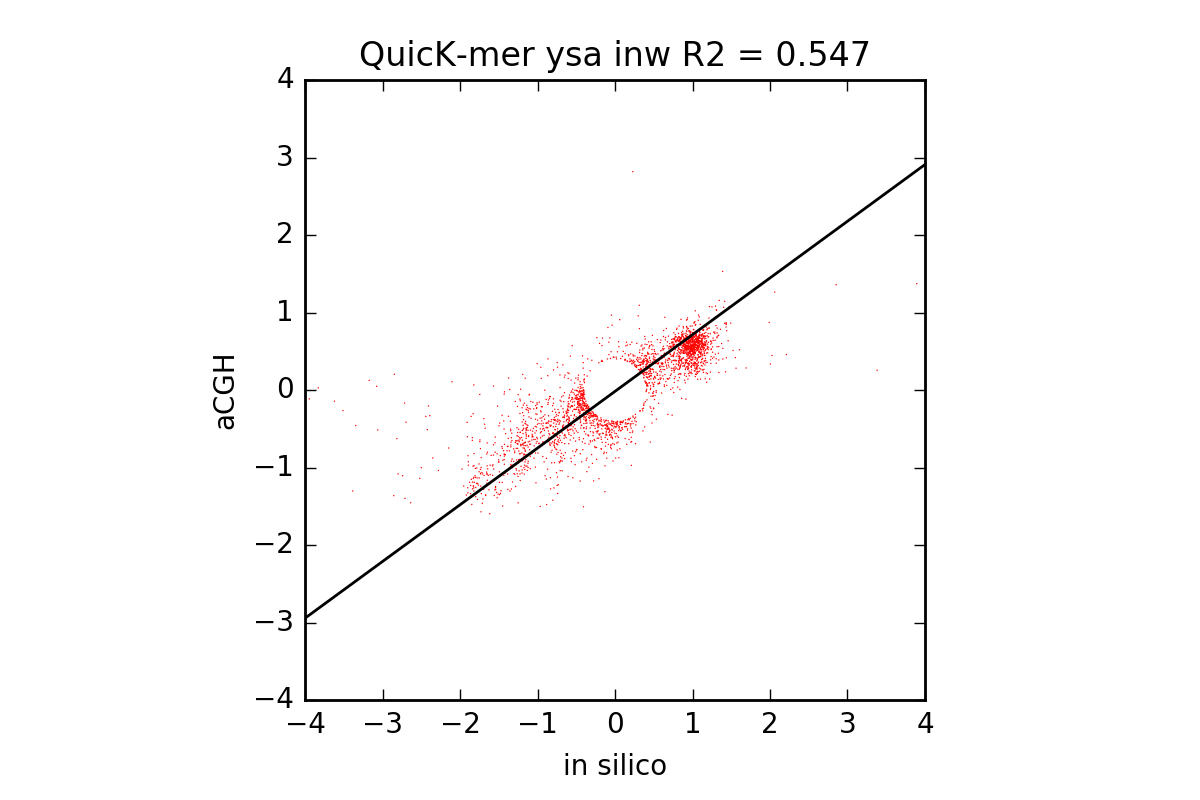

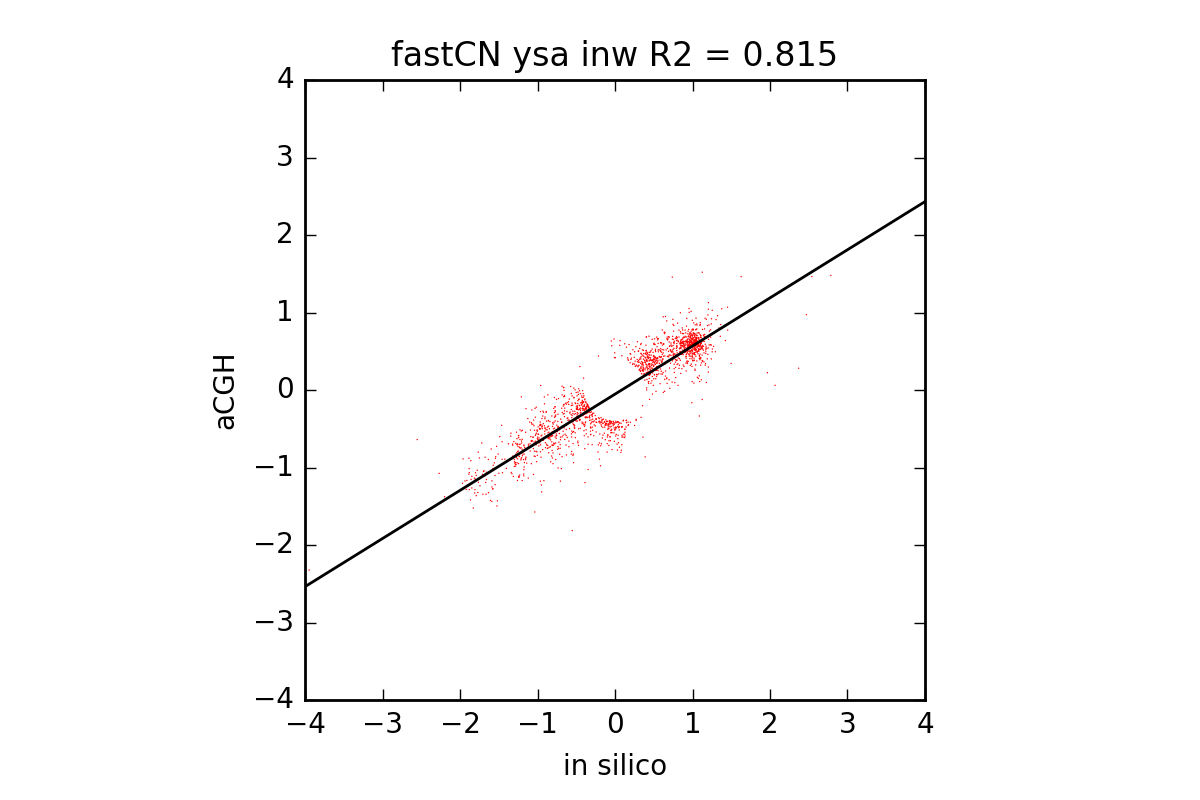


**Figure S3.3.2** Example of scatter plot displaying correlation between our *in silico* copy number estimations and the actual aGCH CN for a Yellowstone wolf (ysa) and an Indian wolf (inw). R^2^ equals 0.55 and 0.81, respectively for QuicK-mer (left) and fastCN (right).

We calculated pairwise correlation coefficient among all seven available wolf samples in Table S3.3.1 and the resulting R^2^ values for 21 comparisons are illustrated in a heatmap in Figure S3.3.3. The average R^2^ value is 0.71 for fastCN and 0.55 for QuicK-mer. Based on the correlation coefficients, we observed that fastCN typically scores higher than QuicK-mer. This could reflect the probe binding chemistry acting in a paralog-insensitive fashion (permitting a certain number of mismatches to hybridize) or that paralog uniqueness was not considered during the probe design. It is also evident that sample pairs with higher coefficient values from QuicK-mer usually have a higher value in fastCN as well, indicating that data from certain samples harbor less noise. In summary, greater than 50% of variance can be explained by this correlation and we employed both methods in the following V_ST_ analysis.


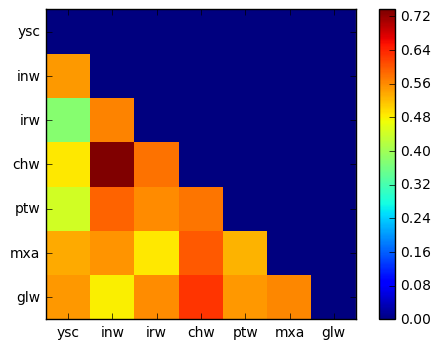

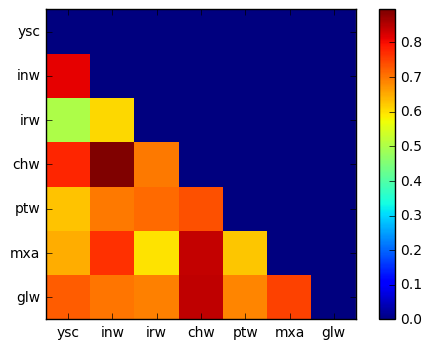


**Figure S3.3.3** Heatmap showing pairwise correlation coefficients (R^2^) between the log ratio of aCGH probe intensities and that of the *in silico* methods from QuicK-mer (left) and fastCN (right)

## 3.4 Detection of CN sweeps through V_ST_ analysis

### 3.4.1 Filtration of genomic regions based on CN estimates

To determine genomic regions with differentiated copy number states between dogs and wolves, we first selected a subset of 3kb windows from the canine genome that showed evidence of copy number variation among the studied samples (Figure S3.4.1.1). We selected windows with a copy number range greater than 1.5 (copy number estimates on the non-PAR region of the X chromosome in males were doubled). This filtration step is necessary to limit the subsequent analysis to variable regions, rather than to noisy estimates derived from a large number of invariable windows. Window selection for fastCN (92,037) and QuicK-mer (37,626) were determined independently.


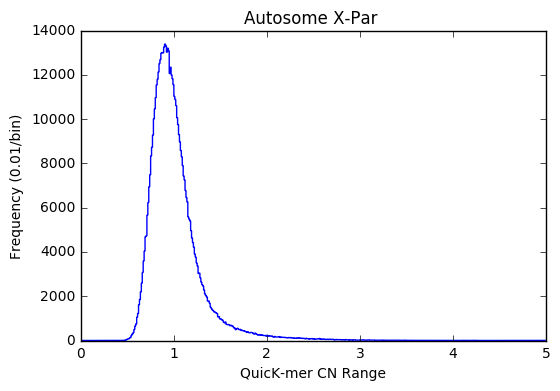

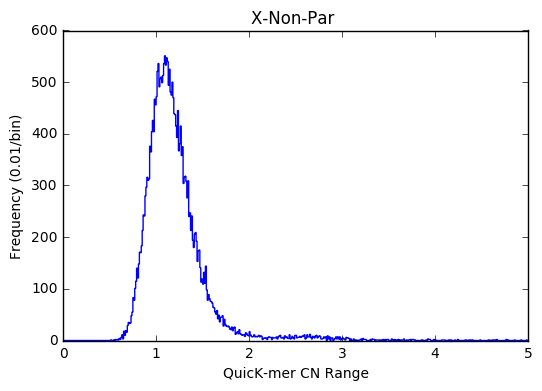


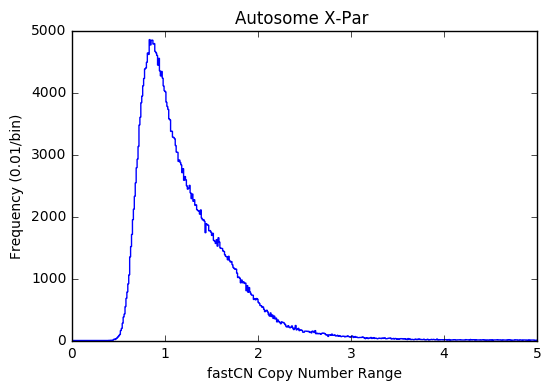

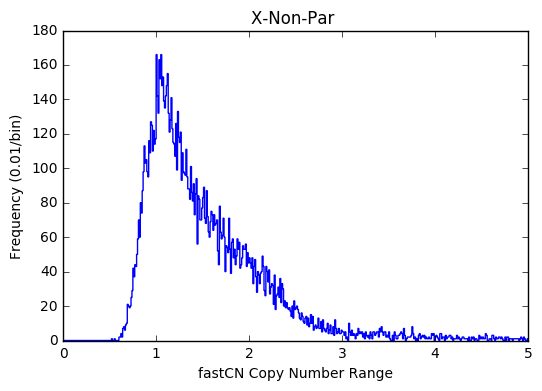


**Figure S3.4.1.1** Distribution of copy number estimates of autosomes plus chrX-PAR (left) and chrX-NonPAR (right) for QuicK-mer (top) and fastCN (bottom) pipelines, respectively, across all samples.

### 3.4.2 Calculation of V_ST_ values

A V_ST_ value for each of the selected 3 kb windows (CN range > 1.5) from Supplementary Note 3.4.1 is then calculated with the following equation (Equation 3.4.2.1) according to [27], where V_T_ and V_S_ denote variance of copy number across the total or sub-population. Calculations were performed separately for QuicK-mer and fastCN estimates.

**Equation 3.4.2.1** $V_{ST}=\frac{V_{T}-V_{S}}{V_{T}}$

The V_ST_ value is similar to a F_ST_ value where a higher V_ST_ value indicates a greater divergence in copy number between wolves and village dogs. However, the value alone will not indicate which population has increased or decreased copy number. We therefore calculated the average copy number in each window for wolves and village dogs separately. The V_ST_ value distribution for each pipeline is illustrated in Figure S3.4.2.1. We observed a narrower distribution for QuicK-mer V_ST_ values, likely because duplicated regions are prone to additional copy number variation and QuicK-mer only interrogates unique regions in the genome assembly.


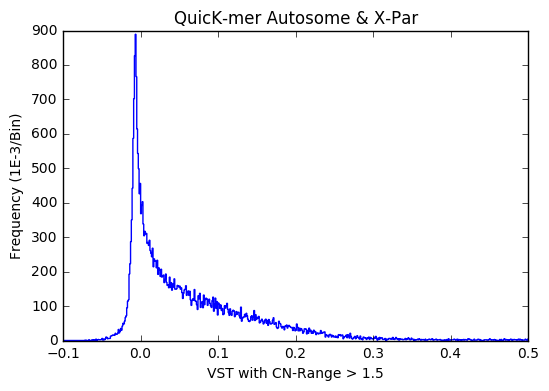

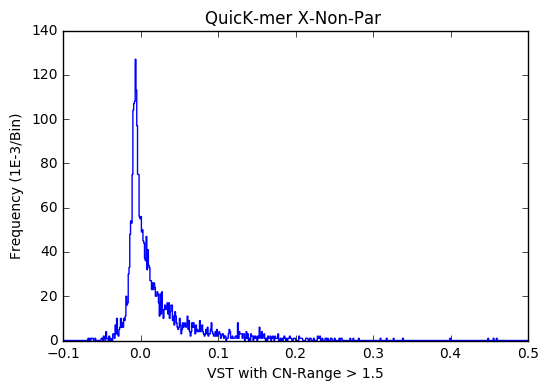


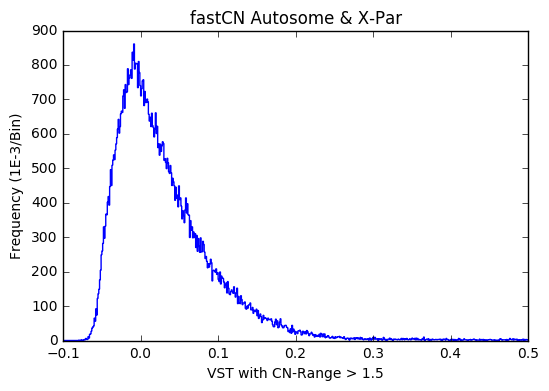

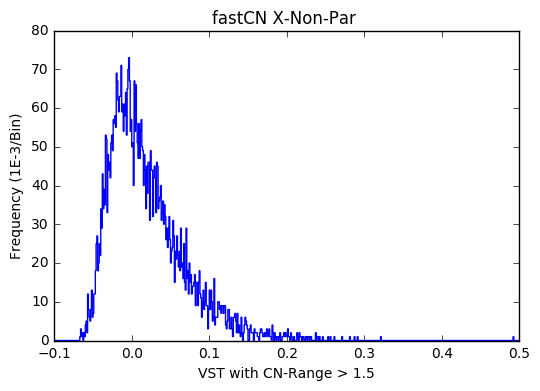


**Figure S3.4.2.1** V_ST_ distribution of subsetted windows with copy number range greater than 1.5 for both QuicK-mer (top) and fastCN (bottom), in autosomes + chrX-PAR (left), and chrX-NonPAR (right).

### 3.4.3 Z-score normalization of V_ST_ distribution

The V_ST_ distributions from the windows with CN > 1.5 across all samples were Z-transformed to generate ZV_ST_ scores per window. This transformation was separately completed for the autosomes + chrX-PAR, and the X-nonPAR. Similar to the F_ST_ filtrations, autosomal and chrX-PAR windows with greater than 5 standard deviations (or ZV_ST_ > 5) were selected as significant V_ST_ outliers, while significant chrX-NonPAR windows included all those that achieved ZV_ST_ > 3. The number of windows following each filtration step are detailed in Table S3.4.3.1.

**Table S3.4.3.1** The number of windows at each filtration stage including total windows analyzed in the V_ST_ pipeline, the amount of windows that had >1.5 CN across samples, and the final windows with significant ZV_ST_ scores (greater than 5 for autosomes and chrX-PAR, and >3 for chrX-NonPAR).

| **Source** | **Whole Genome Windows** | **> 1.5 CN Range** | **Significant ZV_ST_ Score** |
| --- | --- | --- | --- |
| QuicK-mer Autosomes + chrX-PAR | 614,143 | 34,682 | 182 |
| QuicK-mer chrX-NonPAR | 28,060 | 2,944 | 11 |
| fastCN Autosomes + chrX-PAR | 366,945 | 86,276 | 513 |
| fastCN chrX-NonPAR | 13,786 | 5,761 | 6 |

### 3.4.4 Generation of candidate domestication regions from V_ST_ results

Windows that met significance thresholds set in Supplementary Note 3.4.3 were selected from the fastCN and QuicK-mer analyses, and within a given pipeline’s dataset, adjacent significant windows were merged into larger windows. From the the original 519 windows with significant ZV_ST_ scores from the fastCN pipeline, 120 windows were generated from merging with other adjacent, significant windows. Similarly, of the 120 windows remained following merging of the 193 significant QuicK-mer windows.

To designate candidate domestication regions (CDRs) from the V_ST_ data, we intersected the significant windows determined from fastCN and QuicK-mer with one another using BEDtools [28]. For all intersections, the minimum start coordinate and the maximum end coordinate of the intersecting window(s) were selected to define a VCDR. Any window unique to fastCN or QuicK-mer was automatically classified as a VCDR. For all resulting VCDRs, the maximum Z-score was extracted from the fastCN and QuicK-mer dataset to evaluate the level of significance of the region from each set or determine if the region was even evaluated in the opposite analysis set. Due to the stringency of requiring unique k-mer sequence in the QuicK-mer pipeline, it is foreseeable that a window analyzed by fastCN would not be present in the final QuicK-mer dataset.

Upon intersection, we identified 202 regions of copy number deviation between dogs and wolves through our V_ST_ pipeline. Of these final windows, 121 regions were found to be significant only by fastCN while 46 windows were identified only by QuicK-mer. Thirty-five windows had significant ZV_ST_ scores from both fastCN and QuicK-mer. Again, QuicK-mer is a much more conservative and restrictive copy number estimator based on its reliance for sufficient unique k-mers in a region. For this reason, we observe considerably fewer windows with QuicK-mer support. However, the 35 windows with support from both pipelines are noteworthy, having large copy number differences between village dogs and wolves.

Due to low genome coverage of some dog samples, confident detection of small CNVs using read depth is difficult [3]. Therefore, the 202 outlier windows from above were further filtered to require at least two adjacent copy number windows from either fastCN or QuicK-mer, or combined.

### 3.4.5 Chromosome unknown analysis

In addition to the autosomes and X chromosome, we also calculated the level of copy number differentiation of unplaced contigs in the CanFam3.1 reference assembly. F_ST_ was not calculated on these contigs because the redundancy of these sequences reduces quality mappability, thus affecting accurate SNP calling. However, copy number changes through V_ST_ analysis could still be assessed with the fastCN and QuicK-mer pipelines. Copy number estimation with mrsFAST (and therefore also fastCN) is limited to a certain number of input chromosomes, so to facilitate this analysis, we merged all 3,228 unplaced contigs (chrUn’s) into a single, continuous chromosome with 200 ‘N’ bases inserted between each contig. Contig-specific 3kb windows were generated for both fastCN and QuicK-mer pipelines requiring that the last window of each contig does not extend into the next or contain ‘N’ bases. Next, the coordinates of each 3kb window were lifted over to the original unplaced contig with its corresponding location in order to assign copy number to a single unplaced contig following processing with both fastCN and QuicK-mer. Finally, the combined chromosome unknown was incorporated into the genome reference during the copy number estimation steps.


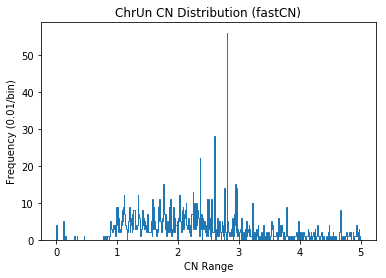

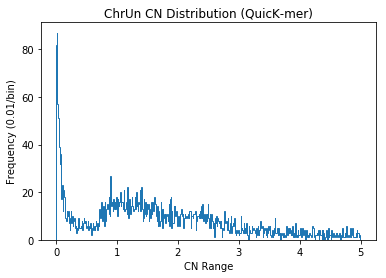


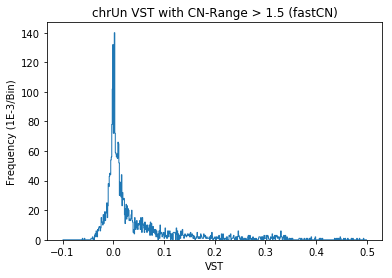

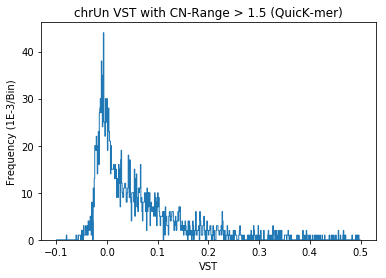


**Figures S3.4.5.1**. The copy number range distribution for chromosome unknown based on fastCN (top left) and QuicK-mer (top right) distincts themselves from that of autosomes or chromosome X. The histogram for VST were shown after filtering windows that have less than 1.5 copy number range among all samples.

V_ST_ selection scans were completed on the merged unknown chromosome using methods previously implemented for autosomal V_ST_ scans. (Figure S3.4.5.1) Windows with copy number ranges greater than 1.5 were selected from each pipeline, which included 3,370 windows from fastCN and 2,346 windows from QuicK-mer. Following Z-transformation of these subsetted windows, only windows with Z-scores greater than 5 were selected as candidate V_ST_ sweeps._._Initially, we identified 21 fastCN and 10 QuicK-mer windows with Z-scores greater than five. After merging adjacent significant windows, the reduced to 8 fastCN and 9 QuicK-mer windows (Table S3.4.5.1), however no overlapping region was called by both fastCN and QuicK-mer. Upon further filtration, five fastCN and one QuicK-mer windows remained that consisted of at least two adjacent significant windows, yielding 6 additional candidate VCDRs. The largest of these is found on chrUn_AAEX03020568 which contains the pancreatic alpha amylase-2b (*AMY2B*) gene, a known copy number variable gene [29-32]. Most unmerged windows achieving the V_ST_ threshold discovered by QuicK-mer contain micro-satellites interrupted by unique sequence queried by QuicK-mer. However, the role of this variation in domestication is unclear.

**Table S3.4.5.1** Regions on chromosome unknown revealed by both fastCN and QuicK-mer. Segments greater than one window in size would meet the criteria to be VCDRs.

| **fastCN** | | | | | | | |
| --- | --- | --- | --- | --- | --- | --- | --- |
| Chromosome | Start | End | No. 3kb window | Max VST | Mean CN in dogs | Mean CN in wolves | Mean CN range |
| chrUn_AAEX03020568 | 433 | 38543 | 7 | 0.63487 | 10.7707 | 1.86921 | 17.5122 |
| chrUn_AAEX03024353 | 36 | 8845 | 2 | 0.634171 | 11.5489 | 1.91201 | 18.3228 |
| chrUn_AAEX03024600 | 7409 | 7889 | 1 | 0.623222 | 4.85336 | 12.0129 | 14.6209 |
| chrUn_AAEX03025786 | 95 | 4932 | 1 | 0.639081 | 8.4489 | 1.96336 | 12.1835 |
| chrUn_JH373575 | 14842 | 33896 | 4 | 0.66059 | 5.52413 | 16.3035 | 21.2698 |
| chrUn_JH373917 | 683 | 22389 | 3 | 0.633881 | 5.3227 | 13.4491 | 15.8141 |
| chrUn_JH374030 | 36 | 15233 | 2 | 0.648995 | 5.38054 | 13.9423 | 16.6026 |
| chrUn_JH374046 | 4411 | 11006 | 1 | 0.618721 | 4.34593 | 10.8845 | 13.2208 |
| **QuicK-mer** | | | | | | | |
| chrUn_AAEX03021660 | 560 | 25346 | 1 | 0.626874 | 0.169814 | 1.4167 | 2.463 |
| chrUn_AAEX03022211 | 1381 | 20389 | 1 | 0.689001 | 0.127372 | 1.391 | 2.236 |
| chrUn_AAEX03022212 | 25 | 20386 | 1 | 0.618471 | 2.18914 | 9.3152 | 13.311 |
| chrUn_AAEX03024092 | 132 | 7895 | 1 | 0.656465 | 0.127163 | 1.6749 | 2.692 |
| chrUn_AAEX03026048 | 678 | 3035 | 1 | 0.648537 | 0.0331163 | 1.2794 | 1.933 |
| chrUn_JH373233 | 1996379 | 2045139 | 2 | 0.619935 | 1.79078 | 0.42245 | 2.446 |
| chrUn_JH373337 | 582 | 77223 | 1 | 0.634374 | 0.419674 | 1.3641 | 2.006 |
| chrUn_JH373343 | 15 | 87451 | 1 | 0.608101 | 0.358233 | 1.1457 | 1.725 |
| chrUn_JH373779 | 15 | 21473 | 1 | 0.585038 | 0.378628 | 1.3411 | 1.974 |

### 3.5 Characterization of copy number at *AMY2B* locus

#### 3.5.1 Read-depth data supports the presence of additional structural variation at the *AMY2B* locus

We estimated copy number using short-read sequencing data from each canine listed in Additional File 1: Table S1 with the methodology implemented in [33] and further expanded below. First, in order to minimize the effects of multi-mapping of short reads by mapping software, we masked the CanFam3.1 reference genome using both RepeatMasker and Tandem Repeat Finder [5]. Additionally, a 50 bp sliding window approach with a step-size of 5 bp was utilized to identify 50-mers overrepresented in the genome. Any 50-mers with 20 or more hits (edit distance = 2) were further masked in the genome sequence. Finally, in order to minimize the shadow effect resulting from gaps introduced by read mapping, each masked region was further masked with a 36 bp padding on each side. Altogether, 48% of the genome remained for interrogation after these intensive masking steps.

Next, Illumina reads were then split into non-overlapping 36 bp segments and subsequently mapped (edit distance = 2) to our custom masked reference dog genome using fastCN. In order to obtain copy number estimates, we first GC-corrected the mapped genomic regions and converted resulting read depth values to copy number. To do so, control regions were identified *a priori* as those with no evidence for copy number variation [22-24]. Next, GC content within 400 bp of each position and a loess fit of read depth were used to determine the GC-correction curve. Finally, copy number estimates were calculated from average depth in non-overlapping windows that contained 3 kb of unmasked sequence. Due to the incomplete nature of the *AMY2B* locus on chromosome 6 caused by the expanded tandem duplications, copy number estimates for the *AMY2B* gene were determined from one window located at the 5’ end of the complete *AMY2B* gene model located at chrUn_AAEX03020568:4873-8379 [33].

Botigue et al. first published the estimated copy number of *AMY2B* for the canines listed in Additional File 1: Table S1 and has been reprinted in Figure S3.5.1.1 [33]. Again, this full set of 100 canines includes the 53 dogs and wolves that were analysed in the F_ST_ and V_ST_ pipelines. Importantly, the lack of expansion of *AMY2B* in the fox, jackal, coyotes, and most wolves is in agreement with previous reports [29, 31] that diploid *AMY2B* copy number is the ancestral state. Excluding the Spanish wolf, only dogs show variation in estimated *AMY2B* copy number.


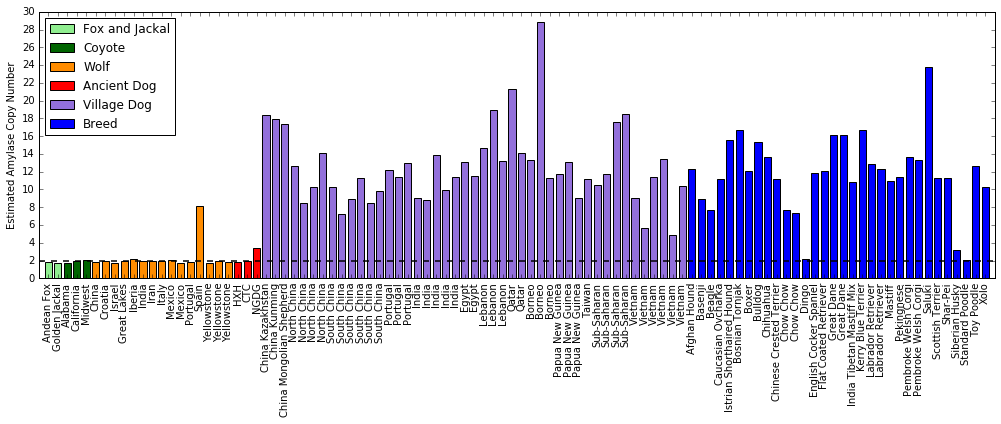


**Figure S3.5.1.1** Estimated copy number of *AMY2B* based on short-read sequence support. CN for outgroups (green), wolves (orange), ancient (fossil) dogs (red), village dogs (purple), and breeds (blue) are provided with CN=2 indicated with a dashed line. Figure reprinted with permission from Botigue et al. 2017.

In addition to the tandem duplication of *AMY2B* at this locus, a significantly larger (~2Mb) duplication was previously observed in dogs using read-depth sequencing data . We have observed that the copy number in our 3kb windows between chr6:45193930-47107035 showed a marked increase in some dogs relative to others in our sample set (Figure S3.5.1.2), predominantly in breed dogs that have a historically European origins (e.g. retrievers, English bulldog, Toy Poodle). With the inclusion of the ancient Newgrange dog from Ireland [34], we were able to associate an approximate age to this duplication since it must be at least as old as this ancient dog (~5,000 years old). The absence of the duplication in wolves and other canines indicates that the while the duplication is present in domesticated dogs, it may not have been acquired during the process of domestication since the two ancient German dogs (Herxheim and Cherry Tree Cave) do not have this large duplication [33, 34]. The distribution of the duplication amongst village dogs does support the its old age, as it found in geographically distinct regions such as Qatar, Papua New Guinea, and Namibia (Figure S3.5.1.2), but it should be noted that these regions do have measurable and historical admixture with European dogs [33, 35]. Such admixture could have facilitated the spread of this duplication.


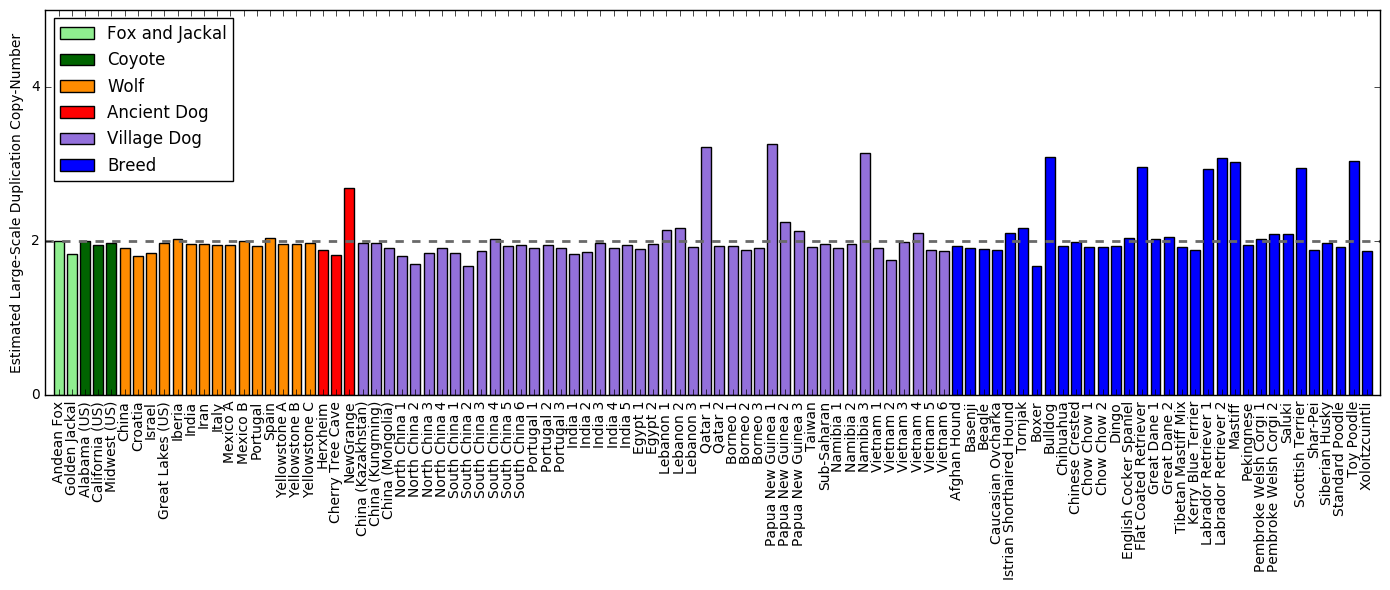


**Figure S3.5.1.2** Average estimated copy number of a large-scale duplication (chr6:45193930-47107035) based on read depth of short-read sequences in 3kb windows. Copy number for outgroups (green), wolves (orange), ancient dogs (red), village dogs (purple), and breeds (blue) are provided with CN=2 indicated with a dashed line.

Upon closer examination of read-depth patterns at both ends of the ~2Mb duplication, we report in this study the presence of two unique duplications, one slightly smaller than the other (Figure S3.5.1.3). For this reason, we will refer to these two large duplications as the 1.9Mb and 2.0Mb duplications because of their approximate lengths. Read-depth patterns show a proximal extension of ~55kb (Figure S3.5.1.4) and a distal extension of ~20kb (Figure S3.5.1.5).


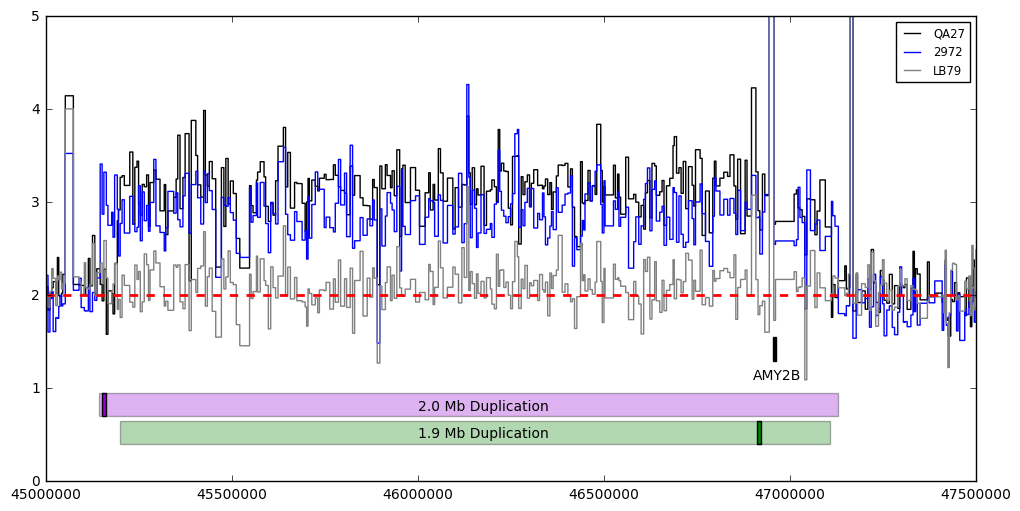


**Figure S3.5.1.3** Read-depth profiles of along chromosome 6 (chr6:45.0-47.5Mb) for three dogs: QA27 (black; Qataran village dog), 2972 (blue; labrador retriever), and LB79 (gray; Lebanese village dog). The diploid copy number (N=2) is indicated by a red, dashed line. The approximate location of the larger (2.0 Mb) duplication is indicated by a purple rectangle, with the position of its ddPCR primer in dark purple. Similarly, the smaller duplication (1.9 Mb) position is shown as a green rectangle with its ddPCR primer in dark green. The relative position of *AMY2B* is indicated in black.


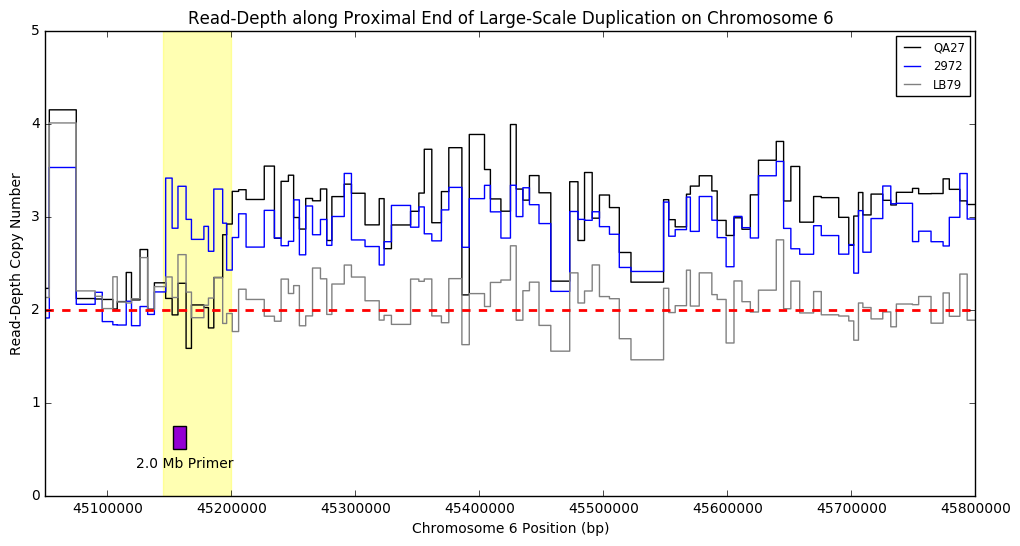


**Figure S3.5.1.4** Read-depth profiles within the proximal end of the large-scale duplication (chr6:45.1-45.8Mb) for three dogs: QA27 (black; Qataran village dog), 2972 (blue; labrador retriever), and LB79 (gray; Lebanese village dog). The diploid copy number (N=2) is indicated by a red, dashed line. Highlighted in yellow is the extended sequence expanded in dogs harboring the 2Mb duplication (blue) versus the smaller duplication (black).


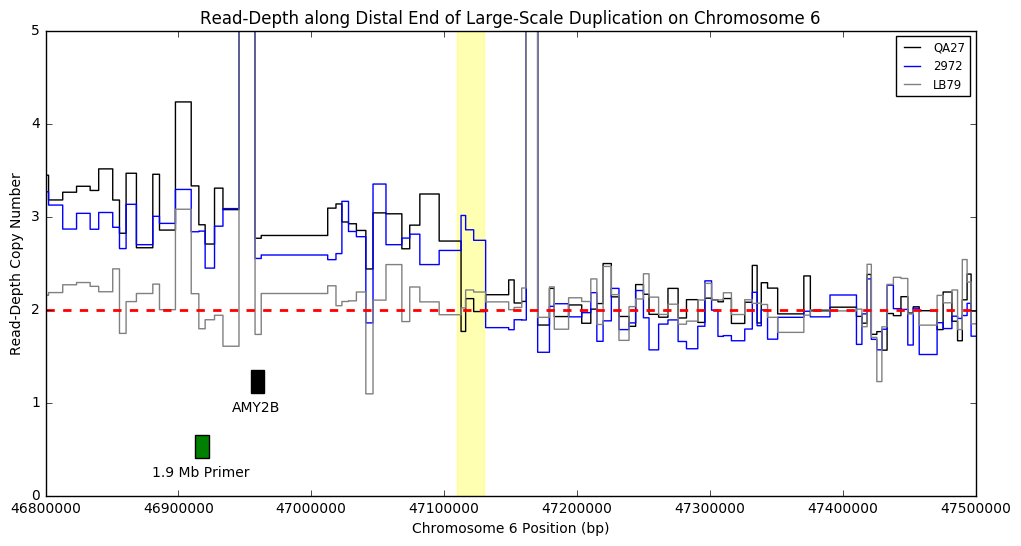


**Figure S3.5.1.5** Read-depth profiles within the distal end of the large-scale duplication (chr6:46.8-47.5Mb) for three dogs: QA27 (black; Qataran village dog), 2972 (blue; labrador retriever), and LB79 (gray; Lebanese village dog). The diploid copy number (N=2) is indicated by a red, dashed line. The black arrow indicates the relative position of the AMY2B gene within the large-scale duplication. Highlighted in yellow is the extended sequence expanded in dogs harboring the 2Mb duplication (blue) versus the smaller duplication (black).

#### 3.5.2 Digital droplet PCR data supports three distinct duplications at the *AMY2B* locus

In order to estimate the copy number of the large-scale duplications, and to determine whether or not the amylase duplication is independent of the larger duplications, we utilized the highly sensitive Digital Droplet PCR technology (ddPCR) (BioRad, Hercules, CA). Determination of the placement of primers for ddPCR was based on a close assessment of the read-depth patterns. Primers suitable for ddPCR were designed for regions that were unique to the proximal end of the 2.0 Mbp duplication, within the 1.9 Mbp duplication and shared with the 2.0 Mbp duplication, and within the *AMY2B* gene sequence (see Figure S3.5.1.3). The primers/probe were synthesized for *AMY2B* based on [30]. In addition, a control region (chr18: 27,529,623-27,535,395) was selected from the genome that obtained consistent copy number estimations equal to two based off of QuicK-mer and fastCN data from all dogs used in our study with sequence data (Additional File 1: Table S1). All primers were designed with Primer3Plus software [36] using settings recommended for ddPCR by BioRad and synthesized at manufacturer’s recommended primer/probe ratio with a 6-FAM (Fluorescein) label on the target probes, a HEX label on the reference probes, and an Iowa Black Quencher on both probes (Integrated DNA Technologies, Coralville, IA). The final forward, internal, and reverse primers are listed in Table S3.5.2.1.

First, we digested 250 or 75 ng of gDNA at 37 °C overnight in a 50-μl or 30-μl reaction of 1xNEB4 with 2.5 U or 0.75 U HindIII. The following morning, an additional 1 U of enzyme was added and the digest continued for another hour. After incubation, the reaction was diluted with 50 μl of H_2_O or 15 μl of H_2_O and 8 μl (40ng) was used for ddPCR.

For ddPCR workflow, a 20-μl mixture containing 0.9 μM primer, 0.25 μM probe, 8 μl (80 ng) of digested DNA, and 1× Bio-Rad ddPCR Supermix for Probes (no dUTP) (BioRad, Hercules, CA) was emulsified, droplets were transferred to 96-well reaction plate, and plate was heat-sealed with foil using manufacturer’s recommended reagents and conditions. We amplified the samples with the following PCR cycling protocol: 10 min at 95 °C, 40 cycles of 30 s at 94 °C and a 1-min extension at 60 °C, followed by 10 min at 98 °C and a hold at 8 °C. After PCR, droplets were read on the droplet reader and data was analyzed using manufacturer’s recommended reagents. “Positive” droplets were identified by fluorescence intensity and thresholds were determined manually for each experiment. We set thresholds based on the ddPCR workflow previously described by [8]. Thresholds were set above the cluster of droplets that were negative for target and reference fluorophores using QuantaCell software. Droplets above this minimum amplitude threshold were counted as positive.

**Table S3.5.2.1** Nucleotide sequences of the ddPCR primers targeting the control region, 1.9Mb duplication, 2.0Mb duplication, and AMY2B gene. The forward (F), internal (int), and reverse (R) primers are provided for each target.

| **Primer** | **Primer Sequence** |
| --- | --- |
| Control_F | CCACCATCGGGTAAACTCAT |
| Control_int | TCCCCATCCTCCCCAGACAGCAGCA |
| Control_R | GTAGGTGTGTTCAGGCAAGA |
| 1.9Mb_Dup_F | TTTAGGCTTAGGCTGGAGCA |
| 1.9Mb_Dup_int | CTCCACCAGCTGTCTCCAGAGGCAAGC |
| 1.9Mb_Dup_R | ACTCAAACTTTTCAAATGGGTGT |
| 2Mb_Dup_F | AGATAAATGCTGTGGGGTGA |
| 2Mb_Dup_int | AAGGCCCAGAGGAGAAGGGTAGCCCA |
| 2Mb_Dup_R | GTTCCCTCTTATTTCCTTTCCC |
| AMY2B_F | CAAACCTGGACGGACATCTa |
| AMY2B_int | TCCATCTGTTTGAGTGGCGCTGGGCT |
| AMY2B_R | CGTTCGCATTCAAGAGCAAt |

Due to limitations of sample availability and quality of DNA, we were only able to analyze copy number by ddPCR for 42 of the canines in our genome sequence set (Additional File 1: Table S1), of which 36 were included in our F_ST_ and V_ST_ analyses (Table S3.5.2.2 and Additional File 1: Table S11). In addition to these 38 dogs, DNA from 48 breed dogs was analyzed (sample information in Table S3.5.2.3 and Additional File 1: Table S12). Though the ddPCR primers and methodology was identical for these two sample sets, replication of the ddPCR runs was only completed for the breed dog sample set, and therefore the average copy number estimates from both runs are provided in Table S3.5.2.3. Both replications of the breed dog set showed little to no changes in copy number estimates of each target and for this reason copy number estimates were performed using only in a single replicate for the village dog samples, where DNA is limited (Table S3.5.2.2).

**Table S3.5.2.2** Sex, sample unique identifier, and *AMY2B*, 1.9 Mb duplication and 2.0 Mb duplication ddPCR copy number estimates are provided for 38 village and breed dogs. Sample IDs with (*) indicate dogs that were excluded from F_ST_/V_ST_ analyses due to high levels of wolf admixture.

| **Sample ID** | **Sex** | **Description** | **Dog Type** | **Average *AMY2B* CN** | **Average 1.9 Mb CN** | **Average 2.0 Mb CN** |
| --- | --- | --- | --- | --- | --- | --- |
| EG44 | Male | Egyptian Village Dog | Village Dog | 11.1 | 2.09 | 1.9 |
| EG49 | Male | Egyptian Village Dog | Village Dog | 8.46 | 2.05 | 1.97 |
| 1735 | Female | Afghan Hound | Breed | 11 | 2.21 | 1.89 |
| Basenji | Male | Basenji | Breed | 9.98 | 2.25 | 1.83 |
| NA8 | Female | Subsaharan African Village Dog | Village Dog | 15.9 | 2.12 | 1.77 |
| NA63 | Male | Subsaharan African Village Dog | Village Dog | 12 | 2.18 | 1.78 |
| NA89 | Male | Subsaharan African Village Dog | Village Dog | 14.1 | 3.28 | 2.87 |
| IN18 | Male | Indonesian Village Dog | Village Dog | 12.1 | 2.16 | 1.86 |
| IN23 | Female | Indonesian Village Dog | Village Dog | 11.3 | 2.2 | 2.19 |
| IN29 | Male | Indonesian Village Dog | Village Dog | 10.7 | 2.13 | 1.98 |
| 2972 | Male | Labrador Retriever | Breed | 12.8 | 3.32 | 2.68 |
| PT49 | Male | Portuguese Village Dog | Village Dog | 11.9 | 2.18 | 1.84 |
| PT61 | Female | Portuguese Village Dog | Village Dog | 10.09 | 2.12 | 1.88 |
| PT71 | Male | Portuguese Village Dog | Village Dog | 11.9 | 2.18 | 1.92 |
| TW04 | Female | Taiwanese Village Dog | Village Dog | 10.76 | 2.24 | 1.83 |
| QA5 | Male | Qataran Village Dog | Village Dog | 12.7 | 2.06 | 1.85 |
| QA27 | Female | Qataran Village Dog | Village Dog | 17.7 | 3.27 | 1.83 |
| LB74 | Male | Lebanese Village Dog | Village Dog | 13.8 | 2.02 | 1.88 |
| LB79 | Male | Lebanese Village Dog | Village Dog | 15.5 | 2.07 | 1.87 |
| LB85 | Female | Lebanese Village Dog | Village Dog | 12 | 1.93 | 1.86 |
| ID4669 | Male | Xolo | Breed | 10.8 | 2.41 | 2.02 |
| HR85 | Female | European Village Dog | Village Dog | 12.3 | 2.13 | 1.93 |
| HR93 | Female | European Village Dog | Village Dog | 10.87 | 2.12 | 2.01 |
| BA19 | Male | Bosnian Tornjak | Breed | 14.3 | 2.13 | 1.91 |
| PG84 | Male | Papua New Guinean Village Dog | Village Dog | 7.15 | 2.29 | 1.87 |
| PG115 | Female | Papua New Guinean Village Dog | Village Dog | 9.17 | 3.23 | 1.89 |
| PG122 | Female | Papua New Guinean Village Dog | Village Dog | 8.55 | 2.13 | 1.88 |
| VN4 | Male | Vietnamese Village Dog | Village Dog | 10.5 | 1.95 | 1.79 |
| VN21 | Female | Vietnamese Village Dog | Village Dog | 8.53 | 2.11 | 1.86 |
| VN37 | Female | Vietnamese Village Dog | Village Dog | 6.94 | 1.86 | 1.86 |
| VN42 | Female | Vietnamese Village Dog | Village Dog | 10.69 | 2.01 | 1.78 |
| VN59 | Male | Vietnamese Village Dog | Village Dog | 5.23 | 2.23 | 1.96 |
| VN76 | Male | Vietnamese Village Dog | Village Dog | 9.76 | 2.18 | 1.88 |
| NGSD1 | Male | New Guinea Singing Dog | New Guinea Singing Dog | 11.66 | 2.19 | 1.83 |
| NGSD2 | Male | New Guinea Singing Dog | New Guinea Singing Dog | 11.4 | 2.25 | 1.93 |
| NGSD3 | Male | New Guinea Singing Dog | New Guinea Singing Dog | 11.67 | 2.29 | 1.97 |
| ID60* | Male | Indian Village Dog | Village Dog | 9.51 | 2.05 | 1.9 |
| ID91* | Female | Indian Village Dog | Village Dog | 10.8 | 1.95 | 1.8 |
| ID125 | Male | Indian Village Dog | Village Dog | 10.2 | 2.26 | 2.04 |
| ID137 | Male | Indian Village Dog | Village Dog | 9.56 | 2.07 | 1.87 |
| ID165 | Male | Indian Village Dog | Village Dog | 12.3 | 2.14 | 1.9 |
| ID168 | Male | Indian Village Dog | Village Dog | 10.5 | 2.02 | 1.85 |

**Table S3.5.2.3** Sex, sample identifier, and average *AMY2B*, 1.9 Mb duplication and 2.0 Mb duplication ddPCR copy number estimates are provided for 48 dogs from various breeds.

| **Sample ID** | **Sex** | **Breed** | **Average *AMY2B* CN** | **Average 1.9 Mb CN** | **Average 2.0 Mb CN** |
| --- | --- | --- | --- | --- | --- |
| PFZ4D12 | Male | Labrador Retriever | 9.155 | 1.41 | 1.37 |
| PFZ1H03 | Female | Labrador Retriever | 9.605 | 1.99 | 1.965 |
| 1957 | Female | Labrador Retriever | 11.03 | 1.99 | 1.925 |
| 2827 | Female | Labrador Retriever | 12.1 | 3.06 | 2.91 |
| 3659 | Female | Labrador Retriever | 8.61 | 2.085 | 1.92 |
| 3727 | Female | Labrador Retriever | 10.64 | 2.03 | 1.915 |
| 4004 | Female | Labrador Retriever | 11.475 | 1.97 | 1.885 |
| 4033 | Male | Labrador Retriever | 11.8 | 2.045 | 1.92 |
| PFZ32C08 | Male | Labrador Retriever | 13.5 | 2.04 | 1.955 |
| 5302 | Male | Labrador Retriever | 10.075 | 1.9 | 1.84 |
| 5309 | Male | Labrador Retriever | 12.715 | 2.1 | 1.915 |
| 5546 | Male | Labrador Retriever | 11.31 | 1.92 | 2.035 |
| 2165 | Female | Labrador Retriever | 10.62 | 2.985 | 3 |
| PFZ1F03 | Male | Labrador Retriever | 12.95 | 2.995 | 2.925 |
| PFZ14B12 | Female | Labrador Retriever | 11.28 | 3.06 | 2.94 |
| 1297 | Male | Labrador Retriever | 11.64 | 4.165 | 3.895 |
| 2127 | Female | Labrador Retriever | 11.06 | 3.21 | 2.835 |
| 3432 | Male | Labrador Retriever | 12.625 | 3.14 | 2.95 |
| PFZ1G03 | Male | Labrador Retriever | 10.355 | 3.005 | 2.86 |
| 3508 | Female | Labrador Retriever | 11.575 | 2.995 | 2.89 |
| 3630 | Female | Labrador Retriever | 11.6 | 4.055 | 3.81 |
| PFZ22D06 | Male | Labrador Retriever | 10.555 | 3.06 | 2.99 |
| 4296 | Female | Labrador Retriever | 11.86 | 3.095 | 2.79 |
| PFZ23D02 | Male | Golden Retriever | 11.435 | 3.105 | 2.905 |
| PFZ29H03 | Female | Golden Retriever | 11.585 | 2.995 | 2.86 |
| PFZ39A01 | Female | Golden Retriever | 11.61 | 3.055 | 2.895 |
| PFZ29E04 | Female | Golden Retriever | 11.685 | 2.015 | 1.925 |
| PFZ44H09 | Male | Golden Retriever | 9.53 | 3.005 | 2.89 |
| PFZ37E10 | Male | Golden Retriever | 12.35 | 2.995 | 2.86 |
| PFZ34D05 | Male | Maltese | 11.55 | 2.045 | 1.885 |
| PFZ31G04 | Female | Mix | 10.365 | 1.975 | 1.855 |
| PFZ9D05 | Male | Samoyed | 3.855 | 2.02 | 2.895 |
| PFZ34B02 | Male | Bernese Mountain Dog | 11.535 | 1.97 | 1.975 |
| PFZ33F04 | Male | Boxer | 9.67 | 2.015 | 1.82 |
| PFZ32C05 | Male | Cavalier King Charles Spaniel | 7.465 | 2.9 | 2.03 |
| PFZ36D10 | Female | English Springer Spaniel | 1.795 | 2.035 | 1.92 |
| PFZ37G10 | Female | German Shepherd | 14.85 | 2.015 | 1.94 |
| PFZ4B07 | Male | Golden Retriever | 11.05 | 2.02 | 1.89 |
| PFZ32H07 | Female | Golden Retriever | 13 | 2.11 | 1.95 |
| PFZ44B10 | Male | Golden Retriever | 10.88 | 2.085 | 1.91 |
| PFZ29H04 | Male | Golden Retriever | 11.39 | 2.07 | 1.93 |
| PFZ34A09 | Male | Maltese | 10.455 | 1.98 | 1.925 |
| PFZ9E05 | Male | Samoyed | 2.745 | 2.02 | 1.945 |
| PFZ44H04 | Female | Standard Poodle | 14.1 | 2.015 | 1.93 |
| PFZ5H05 | Male | German Shepherd | 13.7 | 2.03 | 1.885 |
| PFZ8E12 | Male | Chinese Shar-Pei | 10 | 1.895 | 1.87 |
| 5793 | Male | Labrador Retriever | 10.515 | 2.92 | 2.895 |
| PFZ44B09 | Male | English Springer Spaniel | 12.32 | 2.145 | 1.97 |

Altogether, the ddPCR results confirm the standing variation of *AMY2B* copy number across dogs, but also highlight the presence of two, unique large-scale duplications that encompass the *AMY2B* locus (Figure S3.5.2.1). Overall, the median diploid *AMY2B* copy number analyzed in this diverse sample set was 2*n_AMY2B_* = 11, a value comparable to that found in previous studies [29, 32]. Three breed dogs exhibited very low 2*n_AMY2B_* copy number with 2, 3, and 4 copies in an English springer spaniel and both samoyeds, respectively. Since primers targeting the 1.9Mb duplication would also amplify in dogs with the 2.0Mb duplication, dogs where only the 1.9Mb primer pair showed increased CN but no increase for the 2.0Mb primer would be considered as containing the 1.9Mb duplication. Under this design, two village dogs (QA27 and PG1159) and a Cavalier King Charles spaniel (PFZ32C05) have 2*n*_1.9Mb_ = 3 while 2*n*_2.0Mb_ = 2. Twenty dogs (1 village and 19 breed dogs) have evidence of the 2.0Mb duplication. Excluding the singular village dog (NA89), all dogs with the larger duplication are either Labrador or golden retrievers. Interestingly, there is evidence of 2*n*_2.0Mb_ = 4 for Labradors 18 and 23 (Figure S3.5.2.1) at this region. Only one sample, Labrador Retriever 3 (PFZ4D12), appears to display haploidy for the large scale duplication, as both primer sets returned 2*n_1.9Mb_* = 1. Though, this sample also has 2*n_AMY2B_* = 9. Since we lack genome sequence information for this sample, we cannot test the validity of the haploid call at this locus beyond our ddPCR results.


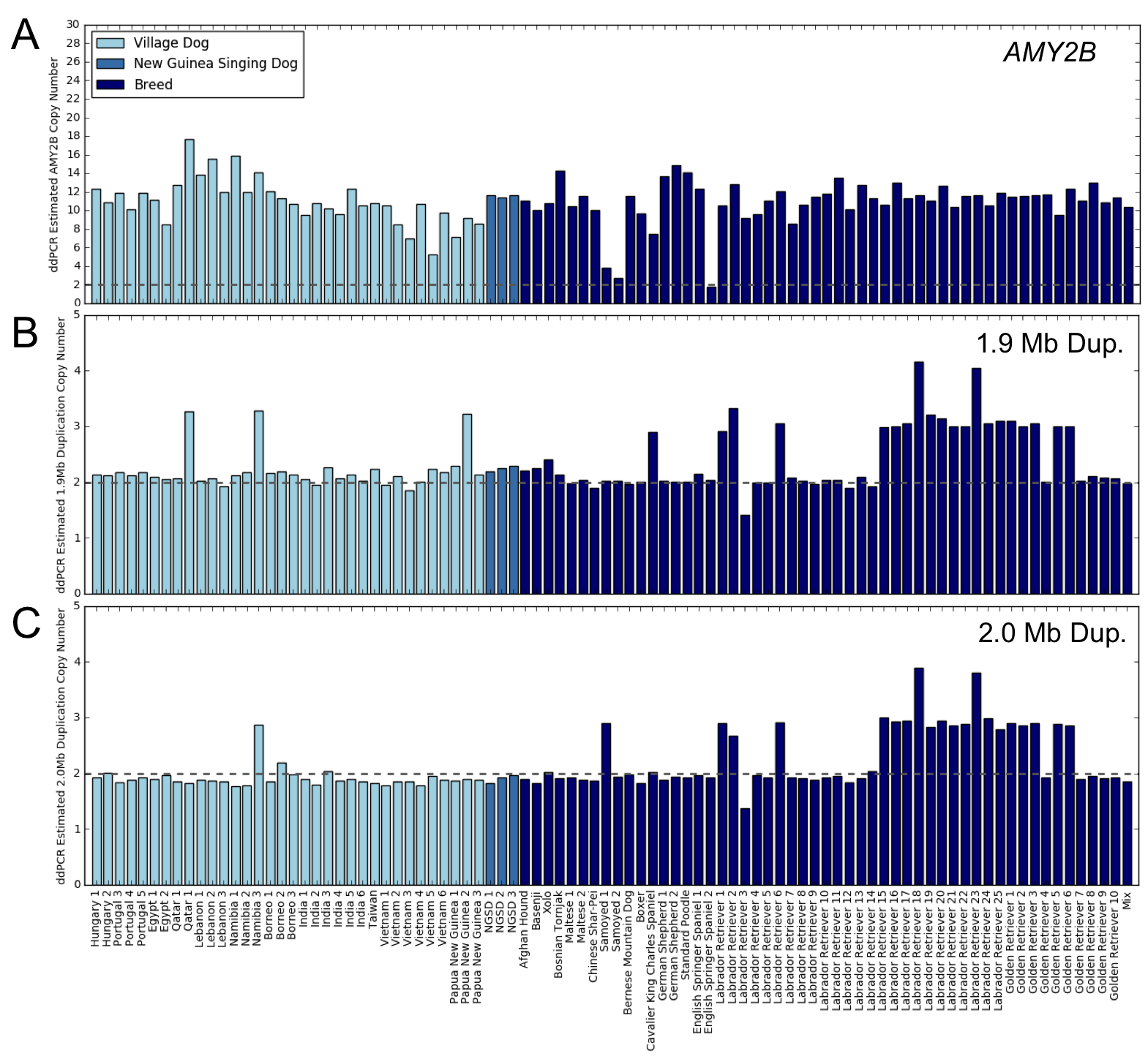


**Figure S3.5.2.1** ddPCR copy number results of the 90 sampled village and breed dogs at the (A) *AMY2B* gene, (B) 1.9 Mb duplication, and the (C) 2.0 Mb duplication. Village dogs are in light blue, New Guinea singing dogs in medium blue, and breed dogs in dark blue. Copy number of two is indicated with a dashed line on each plot.

To determine the accuracy of our read-depth estimations of CN in this region, we assessed the correlation between read-depth and ddPCR estimations of both *AMY2B* and the 1.9Mb duplication. For the 36 samples analyzed by both read-depth and ddPCR approaches,we observed an R^2^ = 0.72 (p-value = 3.429e^-07^) between the read-depth and ddPCR CN estimations of 2*n_AMY2B_* (Figure S3.5.2.2A). Our read-depth estimations of the large-scale duplication strongly corresponded with the ddPCR CN estimation of the 1.9Mb duplication (R^2^ = 0.92; p-value = 1.943e^-16^; Figure S3.5.2.2B).


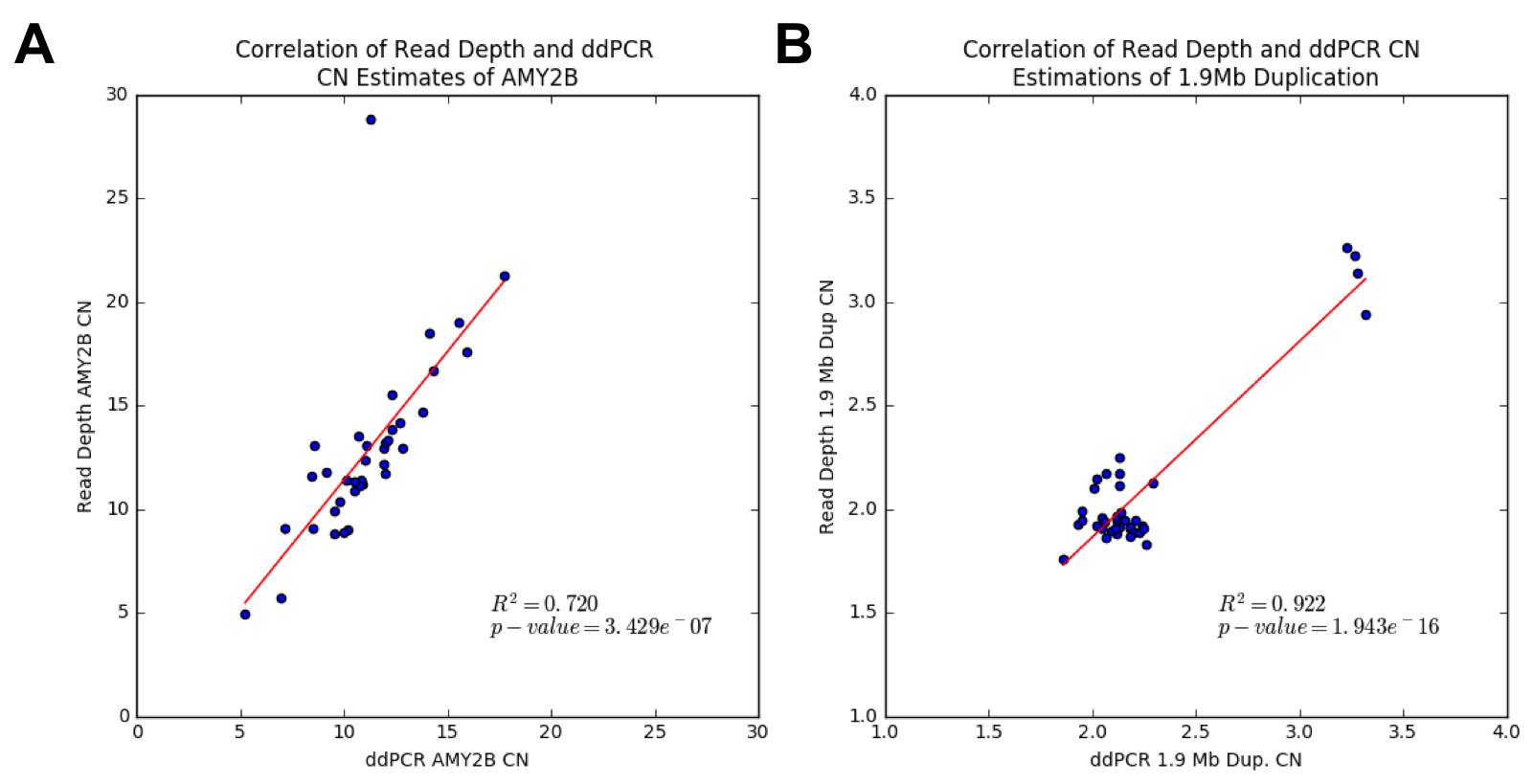


**Figure S3.5.2.2** Correlations between the ddPCR estimated copy number and the read-depth based copy number estimations of (A) the *AMY2B* tandem duplication and (B) the 1.9Mb duplication. For the large-scale duplication, the read-depth data is averaged from all windows within the coordinates of the duplication.

The accuracy of the ddPCR methodology to accurately quantify tandem duplications can be limited by the requirement of , effective restriction digests between individual tandem duplications, which can be difficult to accomplish without sufficient digest time or availability of cut sites between the tandem duplicates. Also, due to extreme CN differences between ddPCR targets, it can be difficult to achieve DNA input concentrations that result in fluorescence of the low CN control above the threshold of detection while also maintaining detection of high CN regions (e.g. *AMY2B*) below the saturation threshold (Figure S3.5.2.3). In our experiments, the estimation of *AMY2B* CN may have an upper bound because in order to detect our control CN, we may have loaded lower concentrations of our *AMY2B* target.


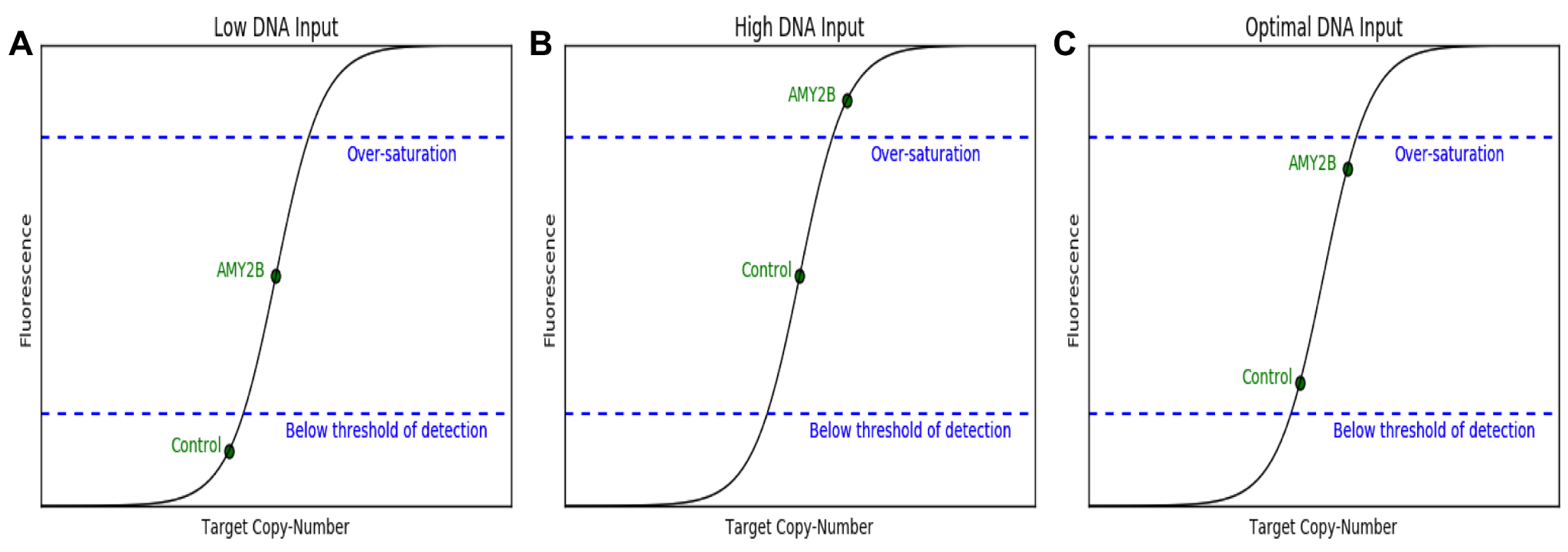


**Figure S3.5.2.3** Hypothetical optimization curves that illustrate the difficulty in determining input DNA concentrations that are sufficient to obtain optimal fluorescence levels for low CN targets (such as the control) versus high CN targets (such as *AMY2B*). Relationships between the two target types are shown under (A) low, (B) high, and (C) optimal input DNA concentrations.

# Works Cited

1. Sudmant PH, Mallick S, Nelson BJ, Hormozdiari F, Krumm N, Huddleston J, et al. Global diversity, population stratification, and selection of human copy-number variation. Science. 2015;349(6253):aab3761.

2. Sudmant PH, Huddleston J, Catacchio CR, Malig M, Hillier LW, Baker C, et al. Evolution and diversity of copy number variation in the great ape lineage. Genome research. 2013;23(9):1373-82.

3. Sudmant PH, Kitzman JO, Antonacci F, Alkan C, Malig M, Tsalenko A, et al. Diversity of human copy number variation and multicopy genes. Science. 2010;330(6004):641-6.

4. Alkan C, Kidd JM, Marques-Bonet T, Aksay G, Antonacci F, Hormozdiari F, et al. Personalized copy number and segmental duplication maps using next-generation sequencing. Nature genetics. 2009;41(10):1061.

5. Hach F, Hormozdiari F, Alkan C, Hormozdiari F, Birol I, Eichler EE, et al. mrsFAST: a cache-oblivious algorithm for short-read mapping. Nature methods. 2010;7(8):576.

6. fastCN.

7. Alkan C, Coe BP, Eichler EE. Genome structural variation discovery and genotyping. Nature Reviews Genetics. 2011;12(5):363.

8. Handsaker RE, Van Doren V, Berman JR, Genovese G, Kashin S, Boettger LM, et al. Large multiallelic copy number variations in humans. Nature genetics. 2015;47(3):296.

9. Zhang Z, Wang W. RNA-Skim: a rapid method for RNA-Seq quantification at transcript level. Bioinformatics. 2014;30(12):i283-i92.

10. Patro R, Mount SM, Kingsford C. Sailfish enables alignment-free isoform quantification from RNA-seq reads using lightweight algorithms. Nature biotechnology. 2014;32(5):462.

11. Bray NL, Pimentel H, Melsted P, Pachter L. Near-optimal probabilistic RNA-seq quantification. Nature biotechnology. 2016;34(5):525.

12. QuicK-mer [Available from: <https://github.com/KiddLab/QuicK-mer>.

13. Marçais G, Kingsford C. A fast, lock-free approach for efficient parallel counting of occurrences of k-mers. Bioinformatics. 2011;27(6):764-70.

14. Consortium GP. An integrated map of genetic variation from 1,092 human genomes. Nature. 2012;491(7422):56.

15. Consortium GP. A map of human genome variation from population-scale sequencing. Nature. 2010;467(7319):1061.

16. Consortium GP. A global reference for human genetic variation. Nature. 2015;526(7571):68.

17. Bentley DR, Balasubramanian S, Swerdlow HP, Smith GP, Milton J, Brown CG, et al. Accurate whole human genome sequencing using reversible terminator chemistry. nature. 2008;456(7218):53.

18. Song S, Sliwerska E, Emery S, Kidd JM. Modeling human population separation history using physically phased genomes. Genetics. 2016:genetics. 116.192963.

19. Hughes JF, Skaletsky H, Pyntikova T, Graves TA, van Daalen SK, Minx PJ, et al. Chimpanzee and human Y chromosomes are remarkably divergent in structure and gene content. Nature. 2010;463(7280):536.

20. Oetjens MT, Shen F, Emery SB, Zou Z, Kidd JM. Y-chromosome structural diversity in the bonobo and chimpanzee lineages. Genome biology and evolution. 2016;8(7):2231-40.

21. QuicK-mer Precomputed K-mers [Available from: <http://kiddlabshare.umms.med.umich.edu/public-data/QuicK-mer/Ref/>.

22. Nicholas TJ, Cheng Z, Ventura M, Mealey K, Eichler EE, Akey JM. The genomic architecture of segmental duplications and associated copy number variants in dogs. Genome research. 2009;19(3):491-9.

23. Nicholas TJ, Baker C, Eichler EE, Akey JM. A high-resolution integrated map of copy number polymorphisms within and between breeds of the modern domesticated dog. BMC genomics. 2011;12(1):414.

24. Freedman AH, Gronau I, Schweizer RM, Ortega-Del Vecchyo D, Han E, Silva PM, et al. Genome sequencing highlights the dynamic early history of dogs. PLoS genetics. 2014;10(1):e1004016.

25. Chen W-K, Swartz JD, Rush LJ, Alvarez CE. Mapping DNA structural variation in dogs. Genome research. 2009;19(3):500-9.

26. Ramirez O, Olalde I, Berglund J, Lorente-Galdos B, Hernandez-Rodriguez J, Quilez J, et al. Analysis of structural diversity in wolf-like canids reveals post-domestication variants. BMC genomics. 2014;15(1):465.

27. Redon R, Ishikawa S, Fitch KR, Feuk L, Perry GH, Andrews TD, et al. Global variation in copy number in the human genome. nature. 2006;444(7118):444.

28. Quinlan AR, Hall IM. BEDTools: a flexible suite of utilities for comparing genomic features. Bioinformatics. 2010;26(6):841-2.

29. Arendt M, Fall T, Lindblad-Toh K, Axelsson E. Amylase activity is associated with AMY2B copy numbers in dog: implications for dog domestication, diet and diabetes. Anim Genet. 2014;45(5):716-22.

30. Axelsson E, Ratnakumar A, Arendt ML, Maqbool K, Webster MT, Perloski M, et al. The genomic signature of dog domestication reveals adaptation to a starch-rich diet. Nature. 2013;495(7441):360-4.

31. Ollivier M, Tresset A, Bastian F, Lagoutte L, Axelsson E, Arendt ML, et al. Amy2B copy number variation reveals starch diet adaptations in ancient European dogs. R Soc Open Sci. 2016;3(11):160449.

32. Arendt M, Cairns KM, Ballard JW, Savolainen P, Axelsson E. Diet adaptation in dog reflects spread of prehistoric agriculture. Heredity (Edinb). 2016;117(5):301-6.

33. Botigue LR, Song S, Scheu A, Gopalan S, Pendleton AL, Oetjens M, et al. Ancient European dog genomes reveal continuity since the Early Neolithic. Nat Commun. 2017;8:16082.

34. Frantz LA, Mullin VE, Pionnier-Capitan M, Lebrasseur O, Ollivier M, Perri A, et al. Genomic and archaeological evidence suggest a dual origin of domestic dogs. Science. 2016;352(6290):1228-31.

35. Shannon LM, Boyko RH, Castelhano M, Corey E, Hayward JJ, McLean C, et al. Genetic structure in village dogs reveals a Central Asian domestication origin. Proc Natl Acad Sci U S A. 2015;112(44):13639-44.

36. Untergasser A, Cutcutache I, Koressaar T, Ye J, Faircloth BC, Remm M, et al. Primer3—new capabilities and interfaces. Nucleic acids research. 2012;40(15):e115-e.
