# Supplementary material for: Comparison of village dog and wolf genomes highlights the role of the neural crest in dog domestication
Source: BMC Biol. 2018 Jun 28;16:64. doi: 10.1186/s12915-018-0535-2 (PMC6022502; doi:10.1186/s12915-018-0535-2)
Supplement: Supplementary file 4 — Supplemental QuicK-mer validation figures. (PDF 1024 kb) [file 12915_2018_535_MOESM4_ESM.pdf]

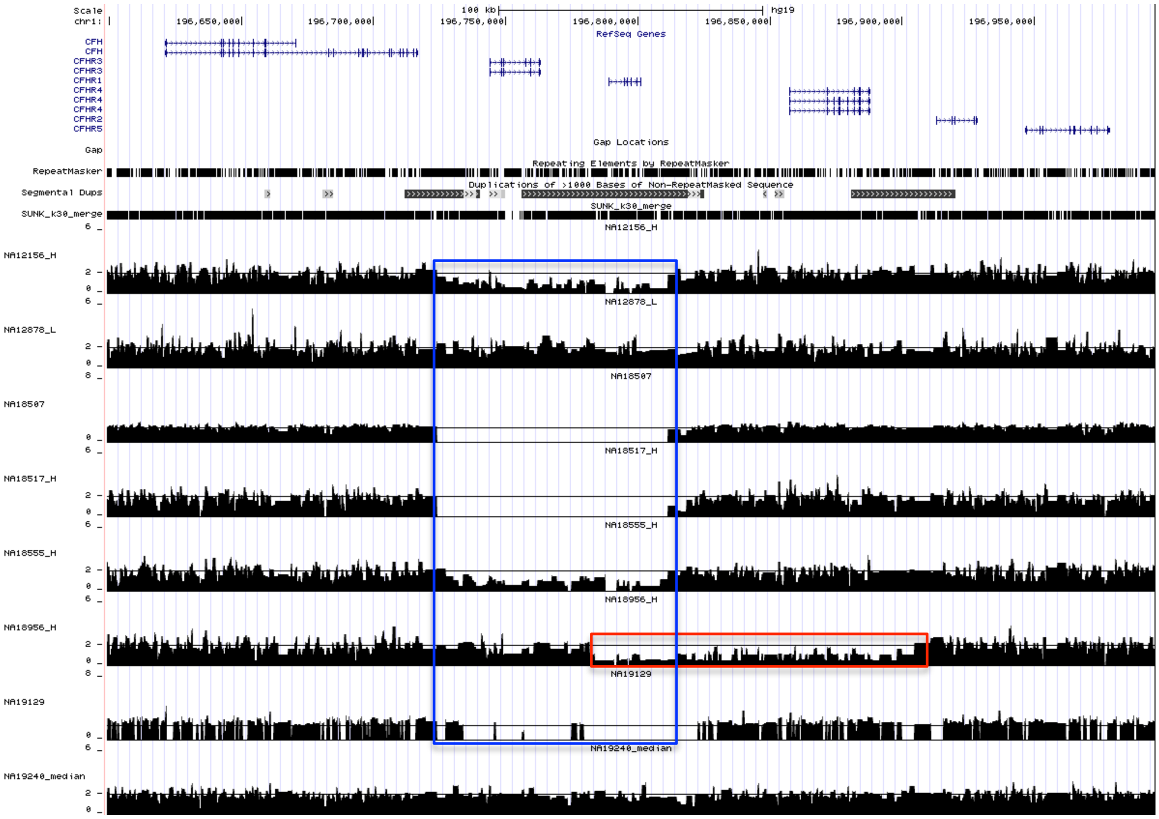

Figure 1
Genome browser screenshot that details two overlapping deletions found in several individuals and match with S60 in Sudmant *et al.,* 2010.

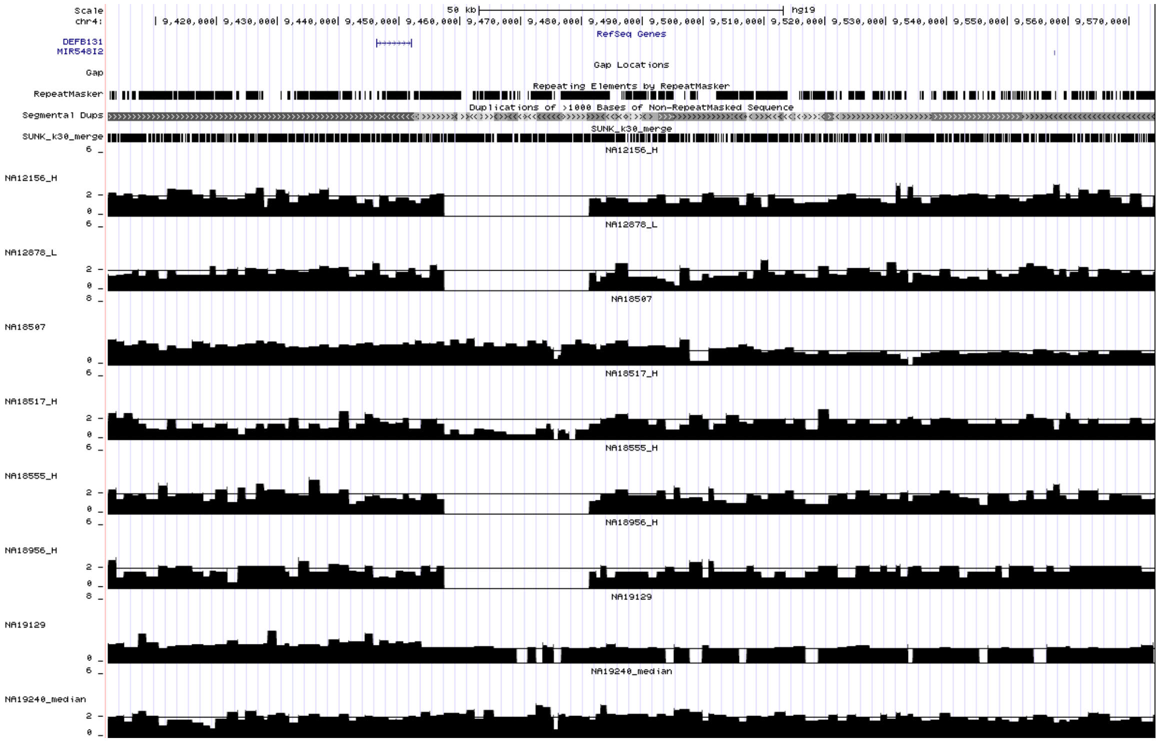

Figure 2

Genome browser screenshot that illustrates deletions detected in 5 individuals and a hemizygous deletion in NA18517. This figure corresponds to S61 in Sudmant *et al.*, 2010.

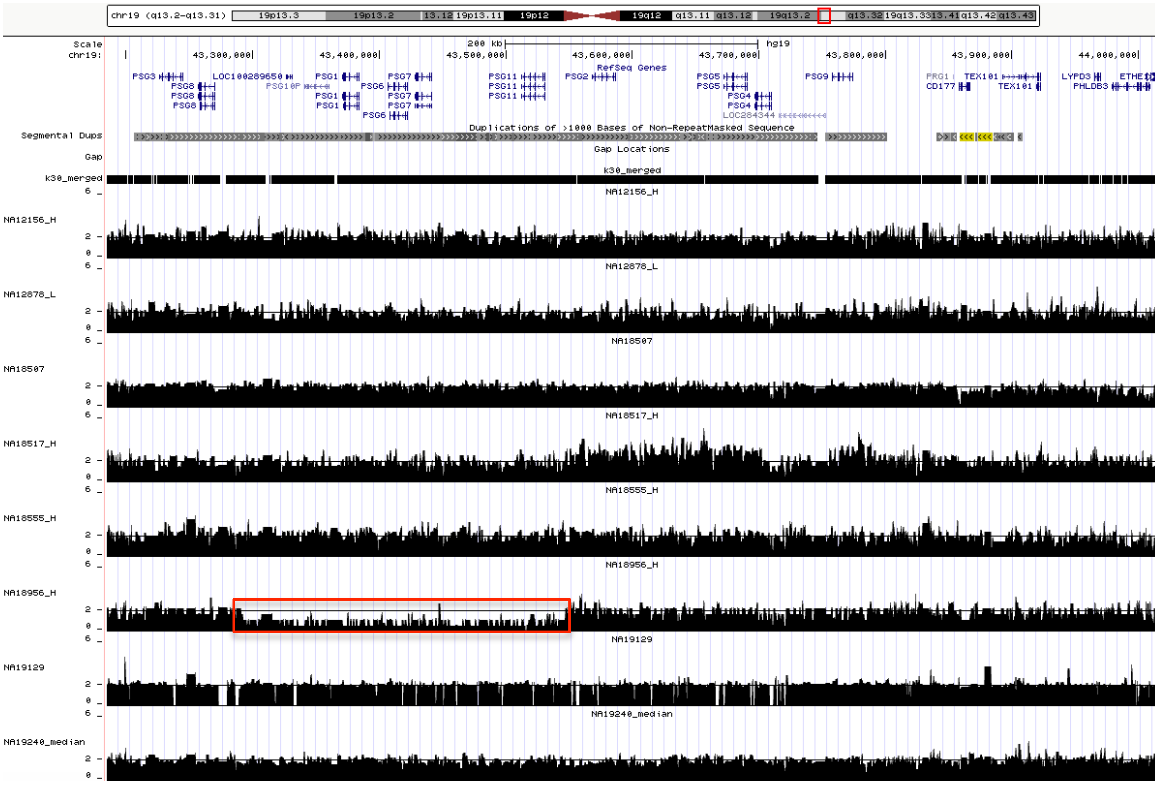

Figure 3

Hemizygous deletion of the *PSG* gene cluster at 19q13.31 in NA18956 within the highlighted region. This figure corresponds to S62 in Sudmant *et al.*, 2010.

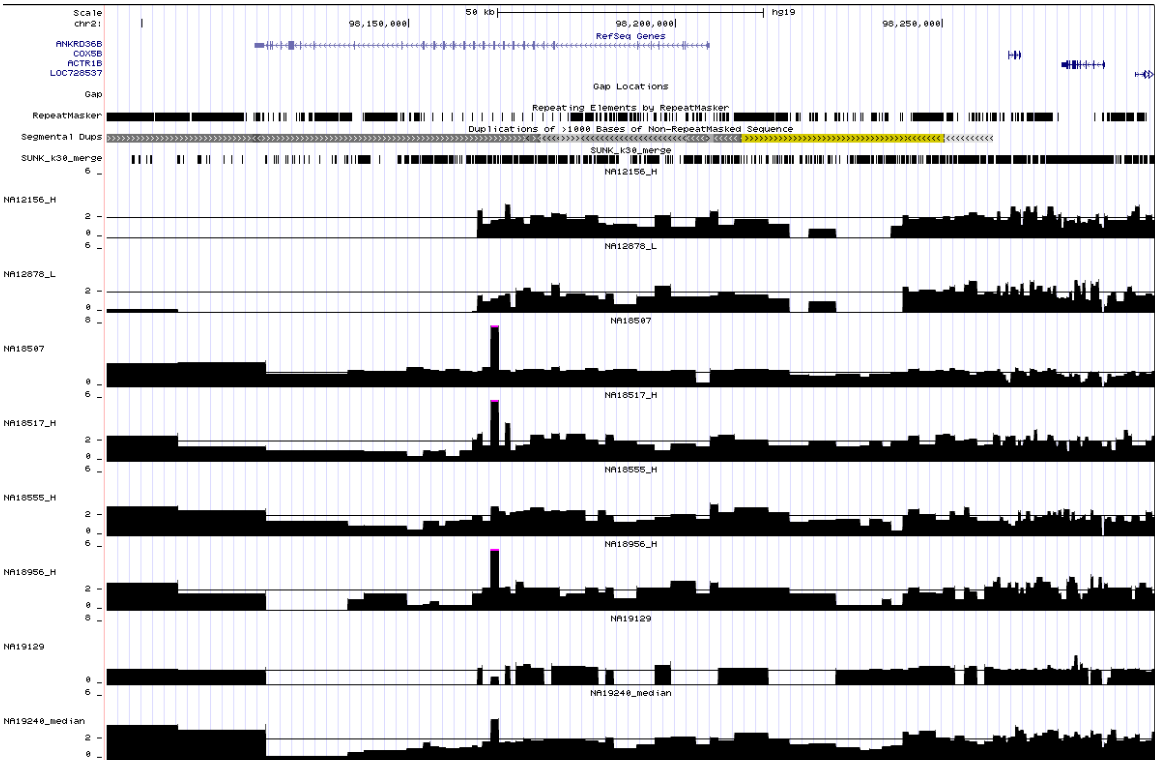

Figure 4

Deletion of the 3' regions of *ANKRD36B* in NA12156 and NA12878, which matches the prediction in S63 in Sudmant *et al.,* 2010.

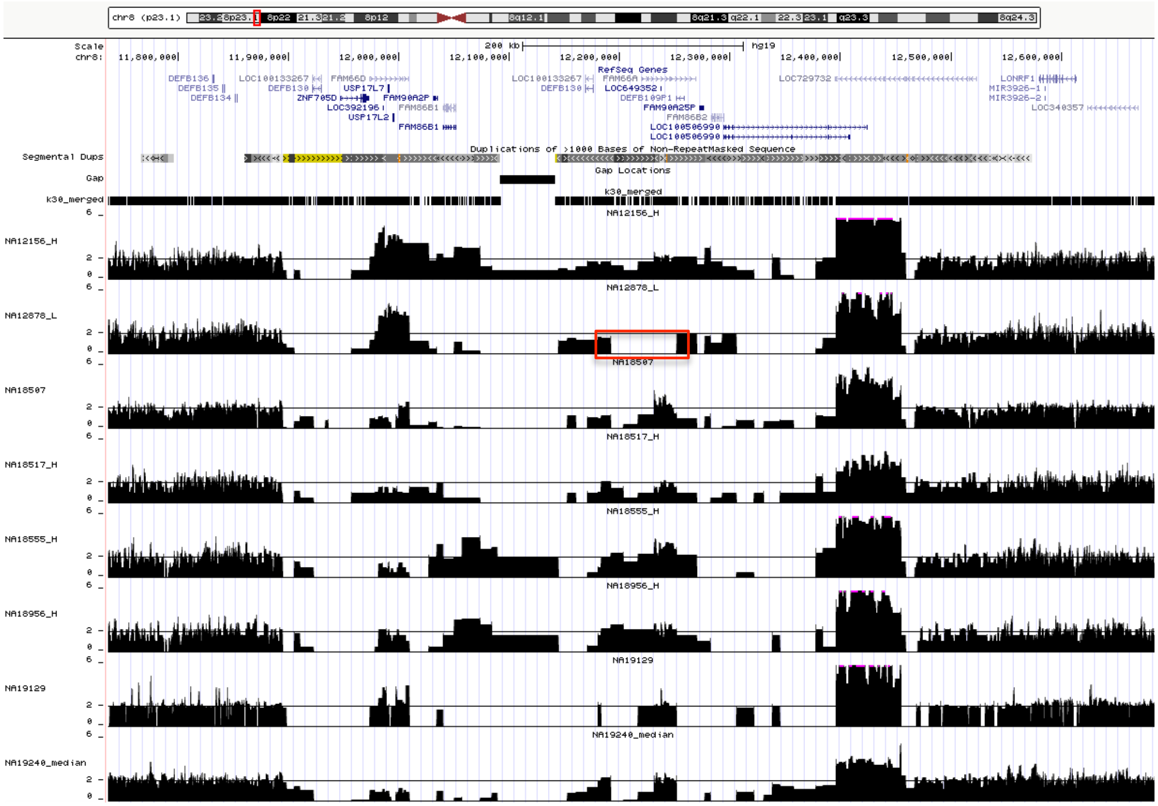

Figure 5

This figure corresponds to S66 in Sudmant *et al.,* 2010 and illustrates a deletion of a defensin gene (observed in NA12878).

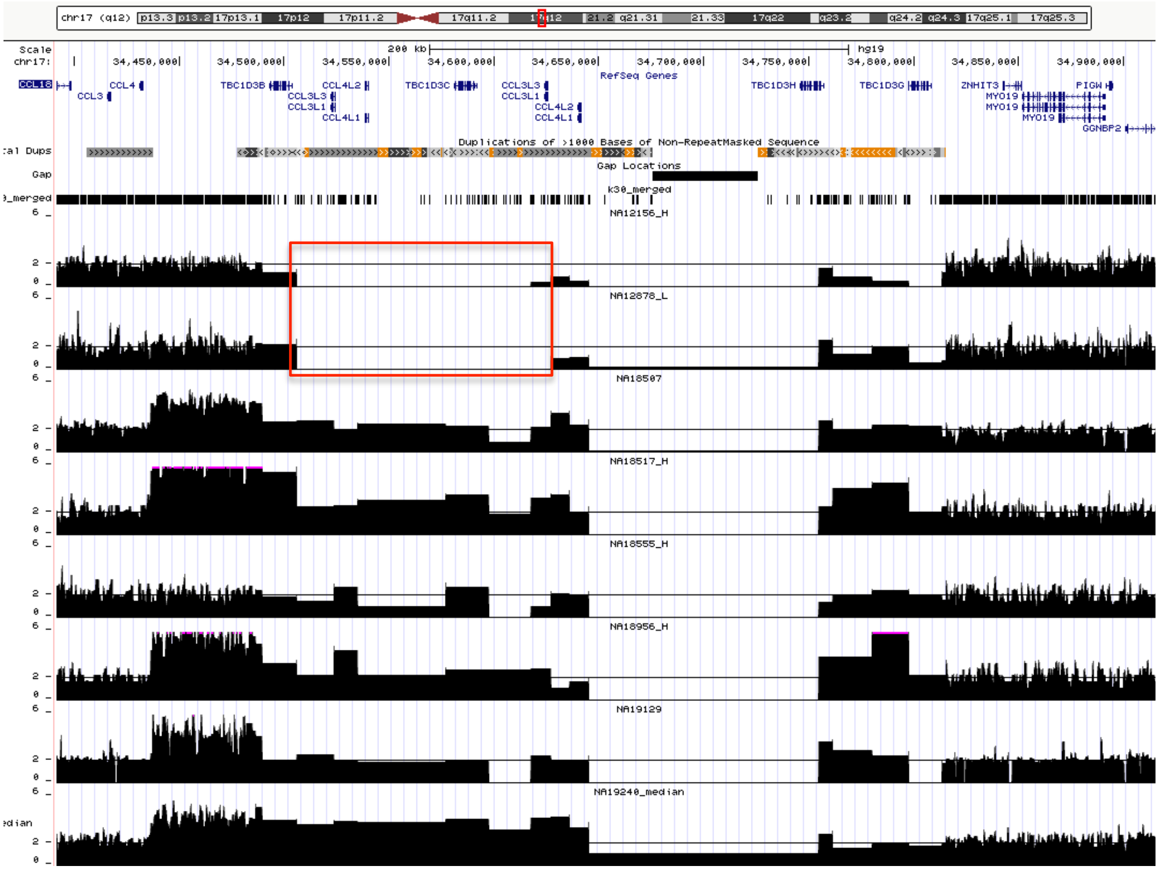

Figure 6

This figure corresponds to S67 in Sudmant *et al.*, 2010. Here, a deletion of chemokine ligand genes (see RefSeq gene IDs in top track) is observed in NA12156 and NA12878.

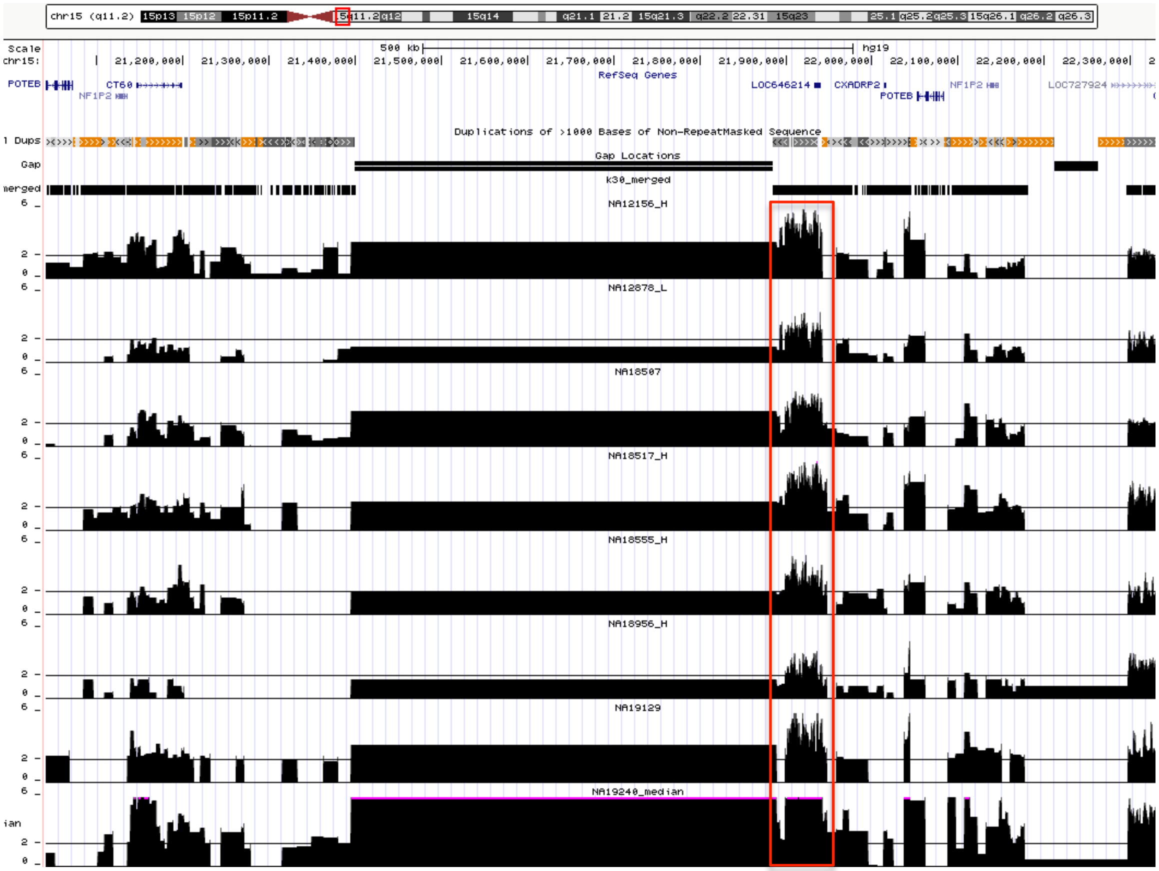

Figure 7

This figure corresponds to S68 in Sudmant *et al.*, 2010. The center window overlaps with an assembly gap and the actual copy number is invalid. An adjacent duplication is observed in all samples shown. NA19240 also exhibits duplication in the olfactory receptor gene clusters (see RefSeq gene IDs in top track).

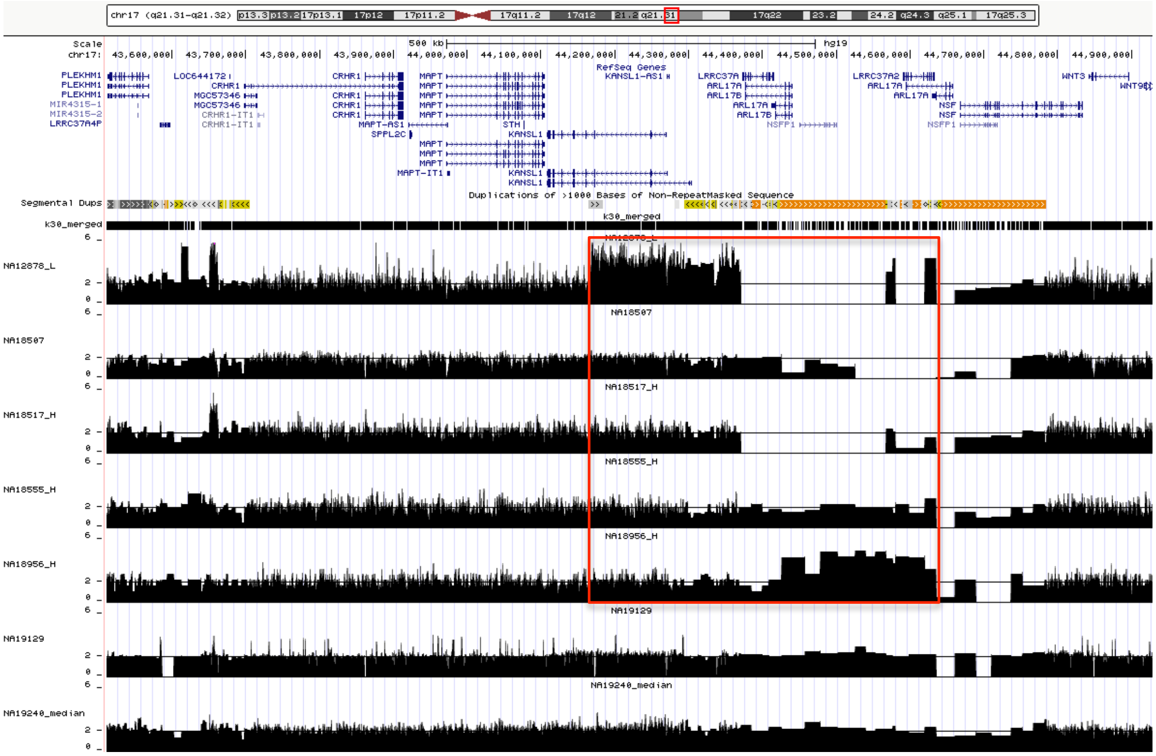

Figure 8

This figure corresponds to S69 in Sudmant *et al*., 2010 and displays segmental duplications in the17q21.31 *MAPT* region.

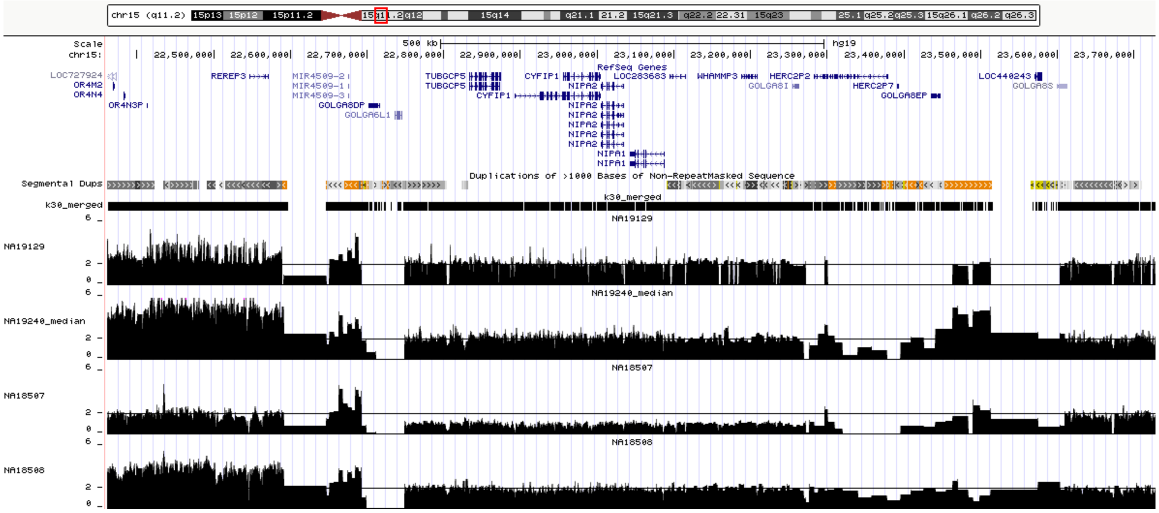

Figure 9

This figure corresponds to S70 in Sudmant *et al*., 2010 and illustrates several gene deletions at 15q11.2 in NA18507.

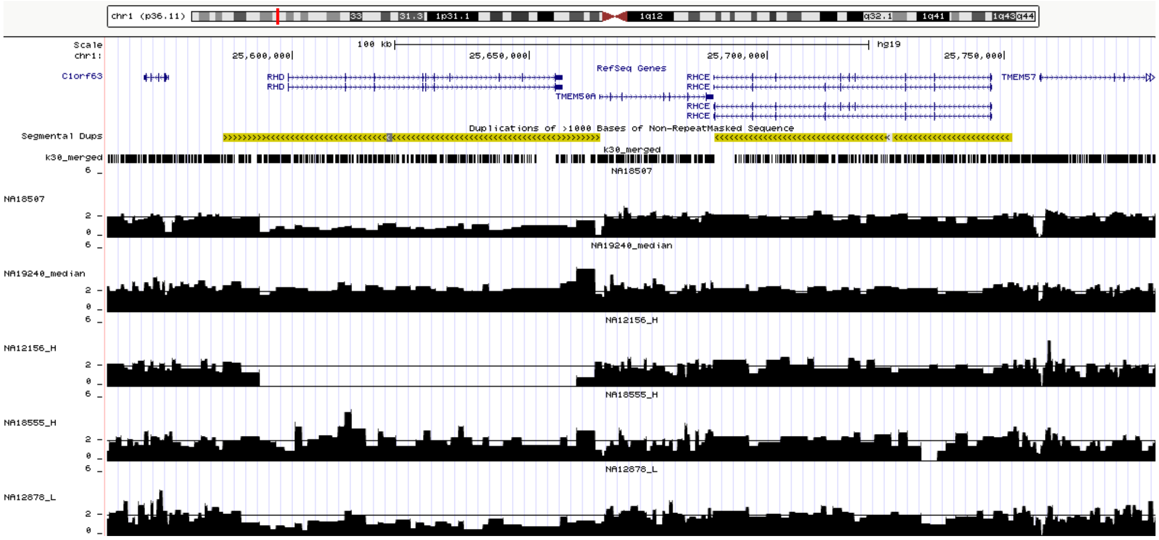

Figure 10

This figure corresponds to S71 in Sudmant *et al*., 2010 and shows copy number variation at the *RHD* and *RHCE* genes. NA18507 and NA12878 each have one copy of RHD deleted.
