# Supplementary material for: Comparison of village dog and wolf genomes highlights the role of the neural crest in dog domestication
Source: BMC Biol. 2018 Jun 28;16:64. doi: 10.1186/s12915-018-0535-2 (PMC6022502; doi:10.1186/s12915-018-0535-2)
Supplement: Supplementary file 5 — Plots displaying aCGH probe intensity correlations with in silico copy number estimates. (PDF 1916 kb) [file 12915_2018_535_MOESM5_ESM.pdf]

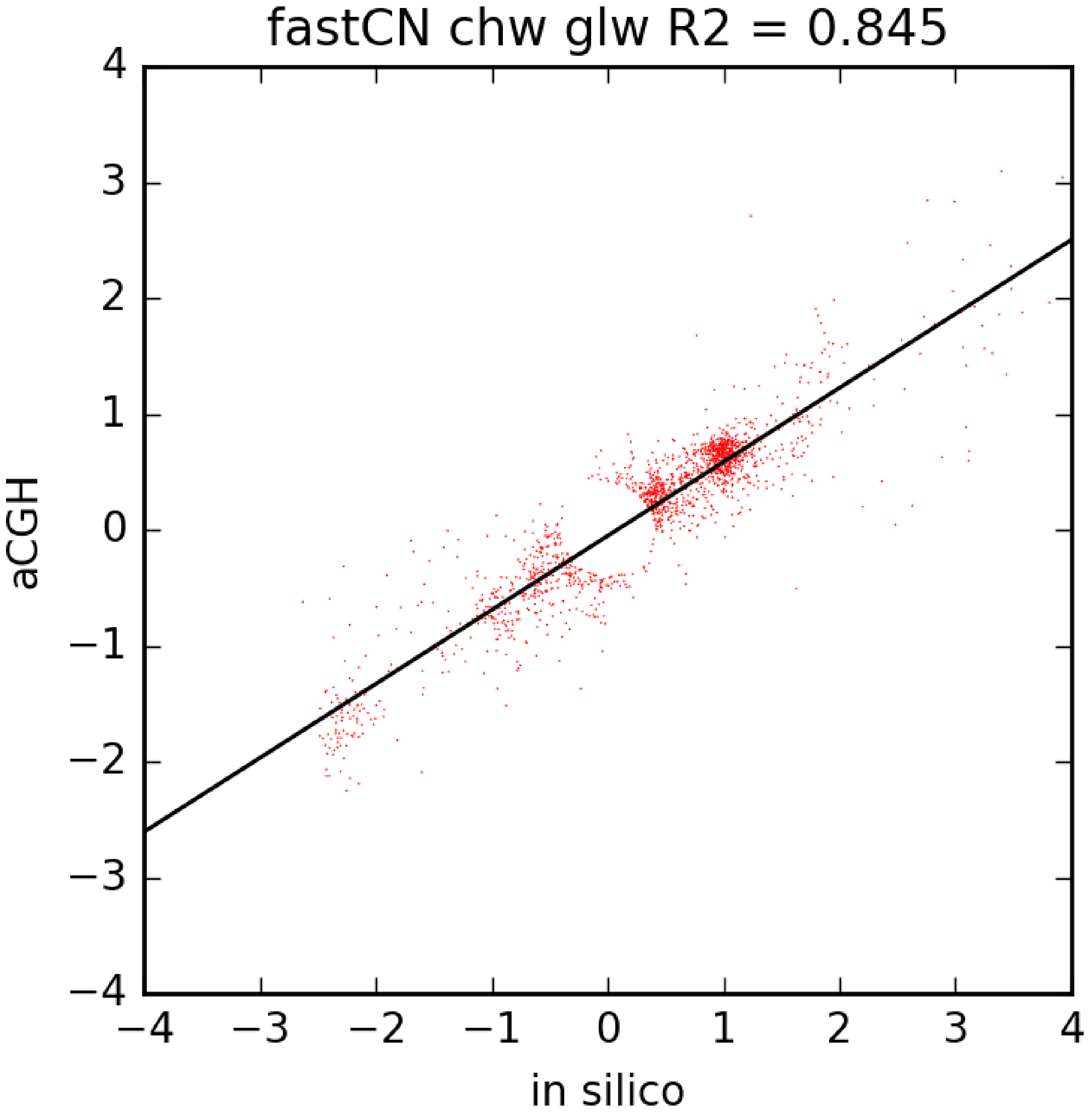

fastCN chw glw R2 = 0.845
aCGH
in silico
4
3
2
1
0
−1
−2
−3
−4

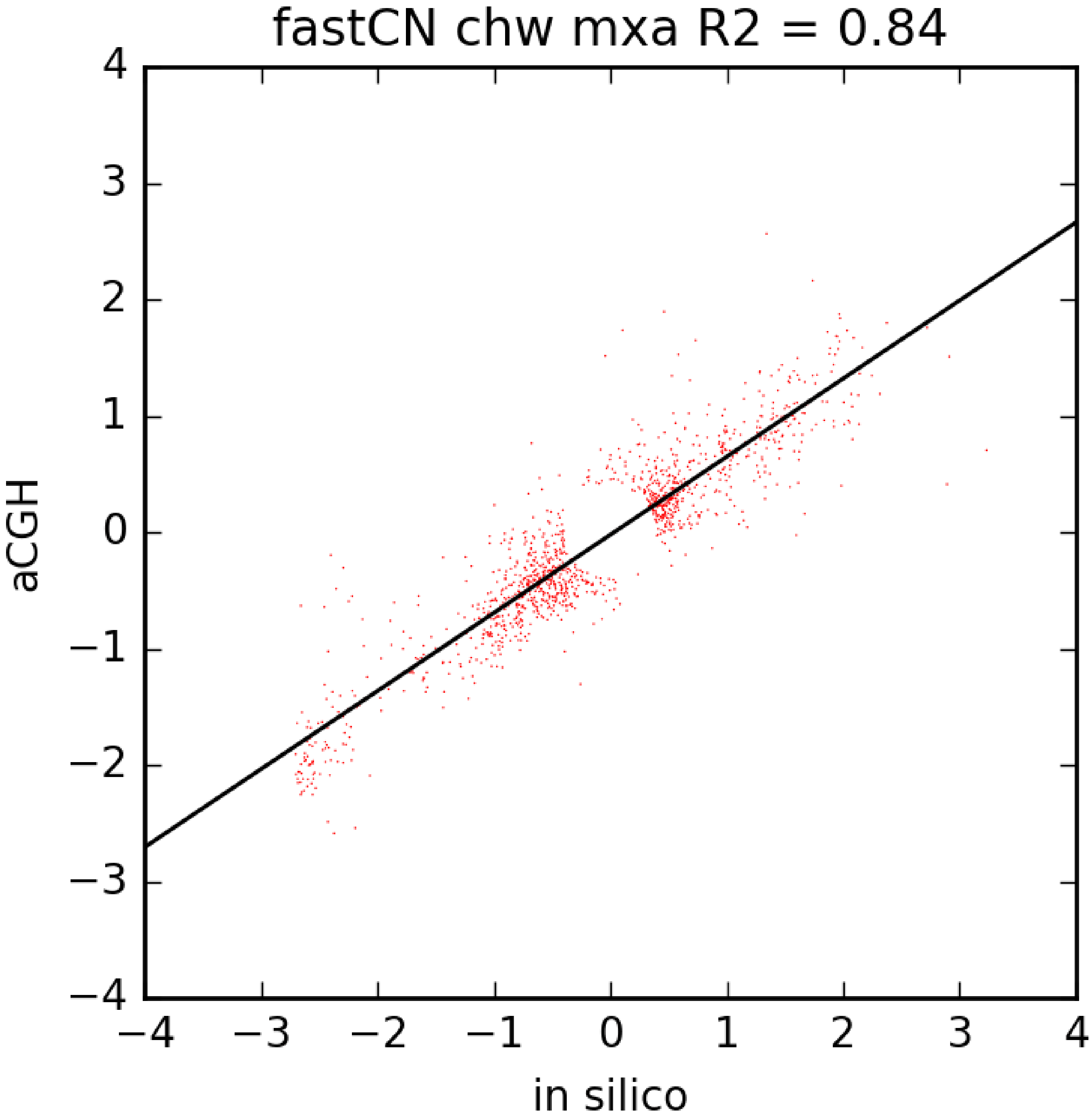

fastCN chw mxa R2 = 0.84
4
3
2
1
0
−1
−2
−3
−4
aCGH
−4
−3
−2
−1
0
1
2
3
4
in silico

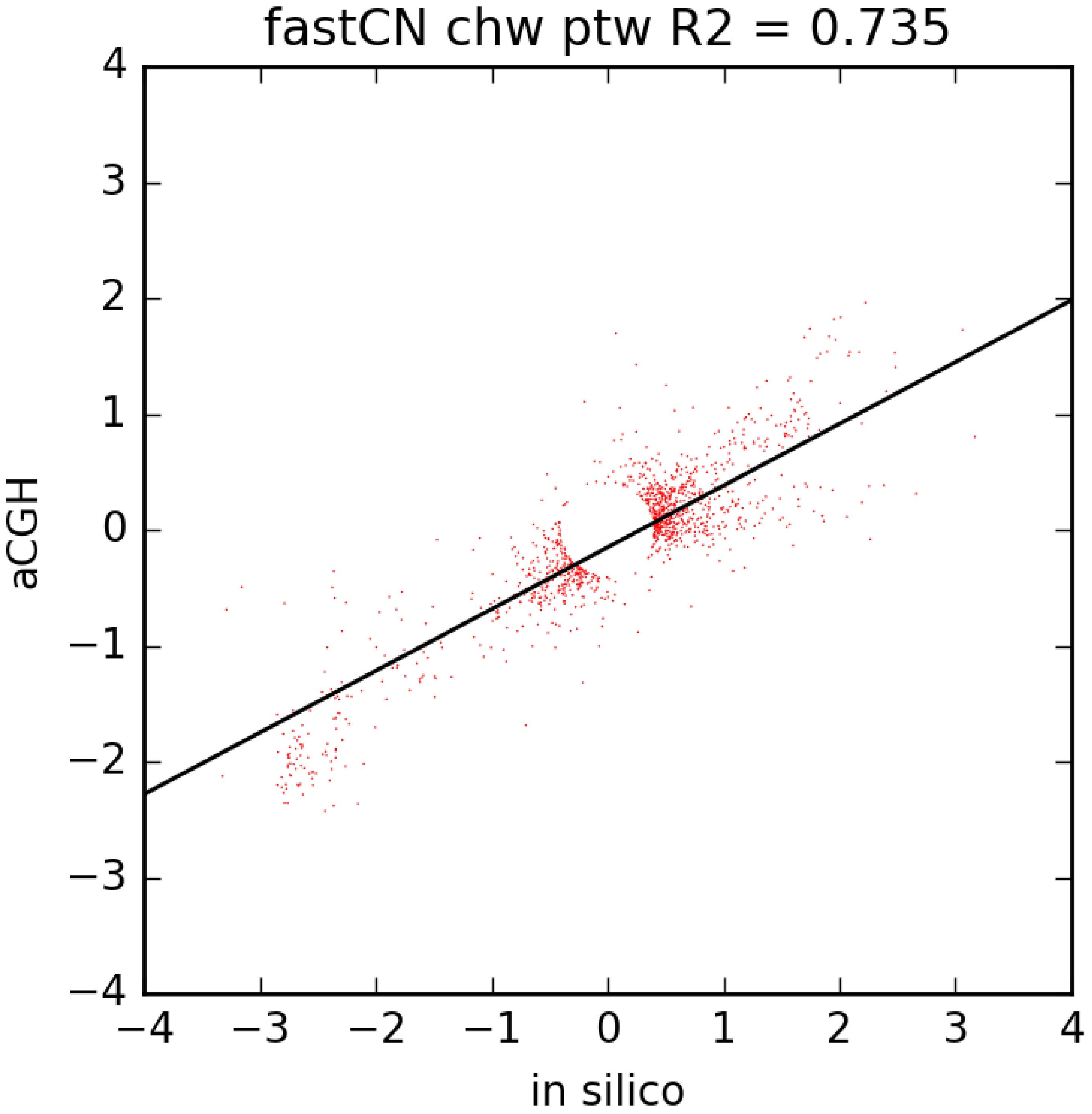
fastCN chw ptw R2 = 0.735
aCGH
in silico
4
3
2
1
0
−1
−2
−3
−4
−4
−3
−2
−1
0
1
2
3
4

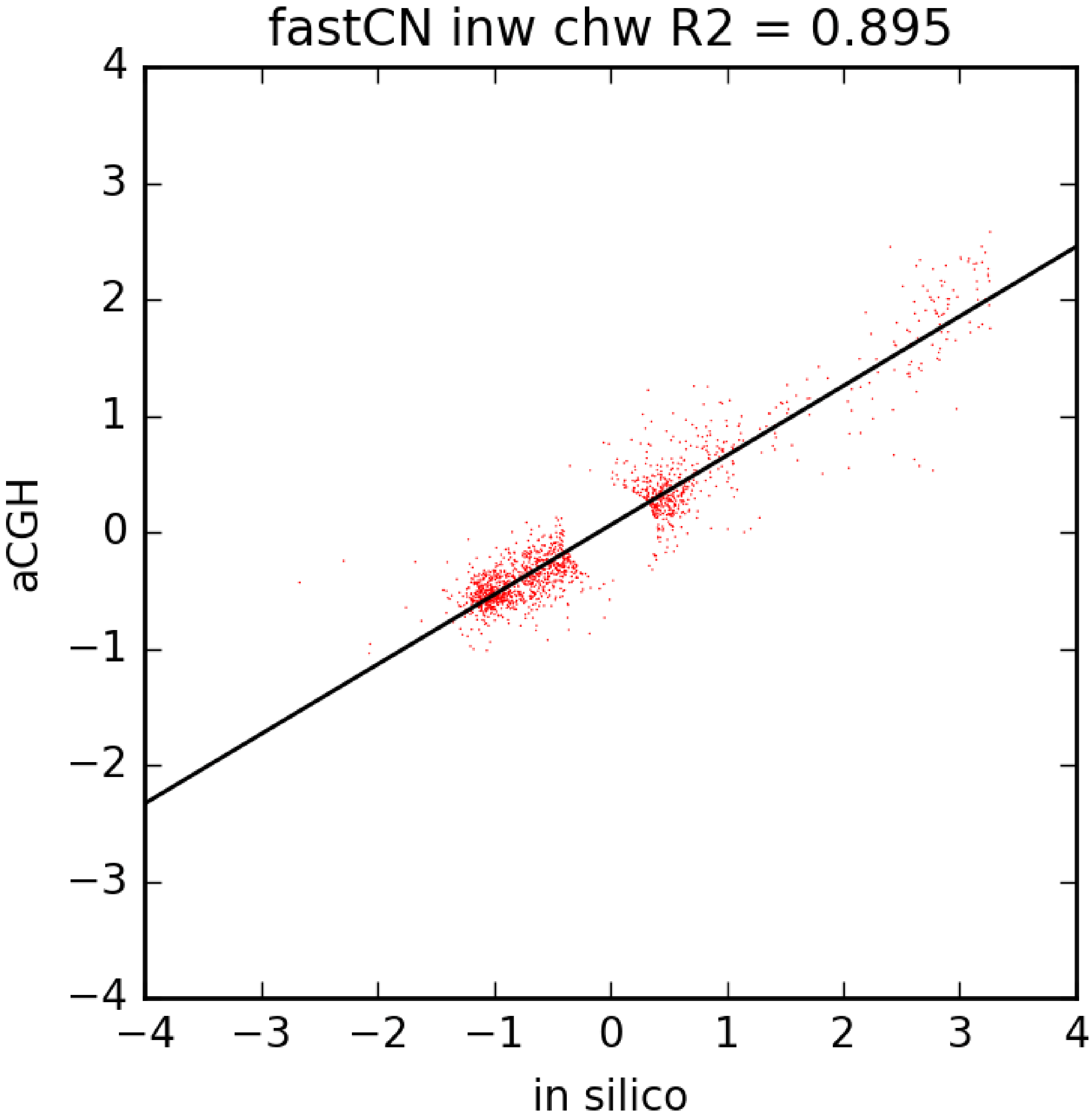

fastCN inw chw R2 = 0.895
aCGH
in silico
4
3
2
1
0
−1
−2
−3
−4
−4
−3
−2
−1
0
1
2
3
4

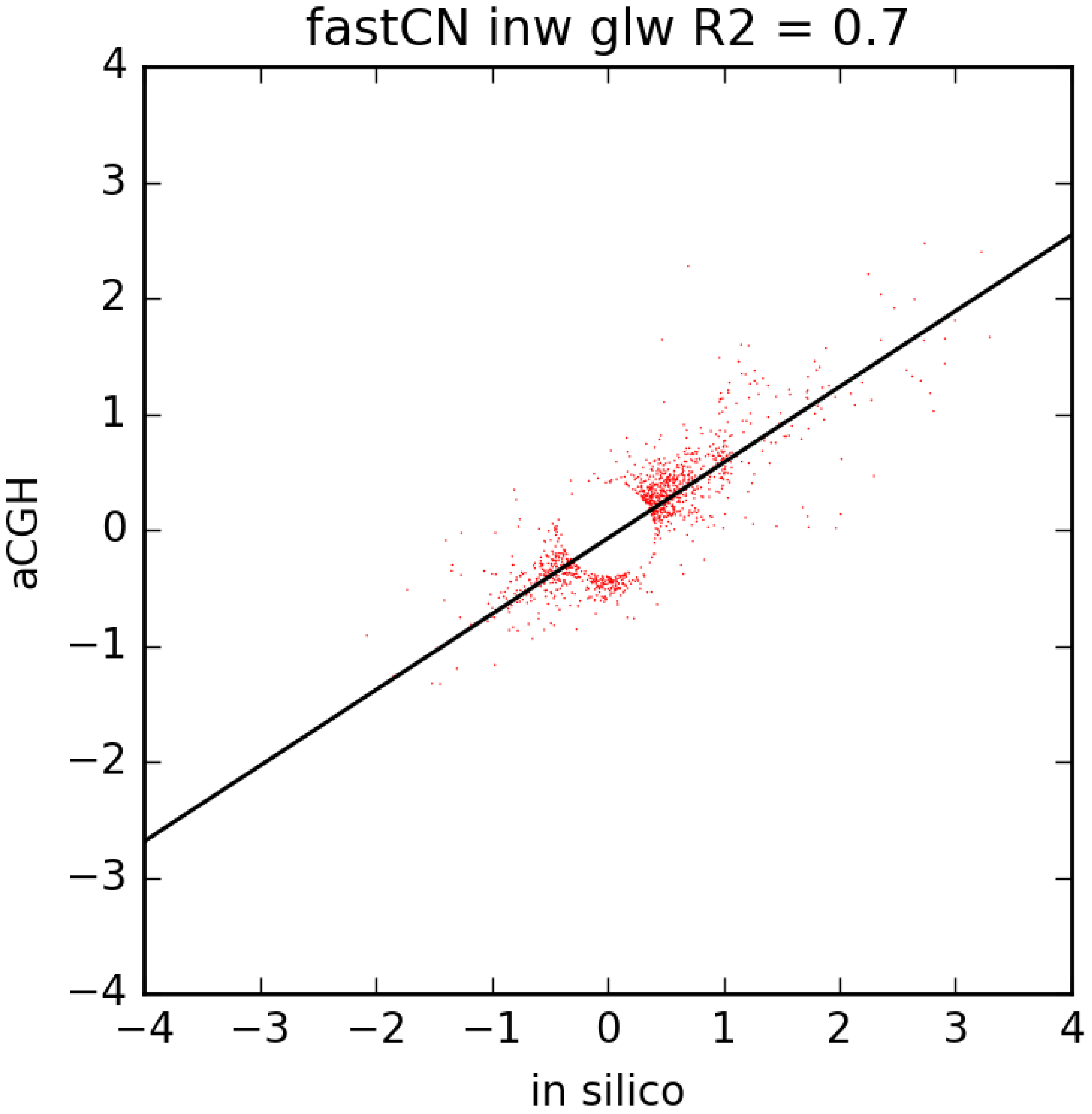
fastCN inw glw R2 = 0.7
aCGH
in silico
4
3
2
1
0
−1
−2
−3
−4

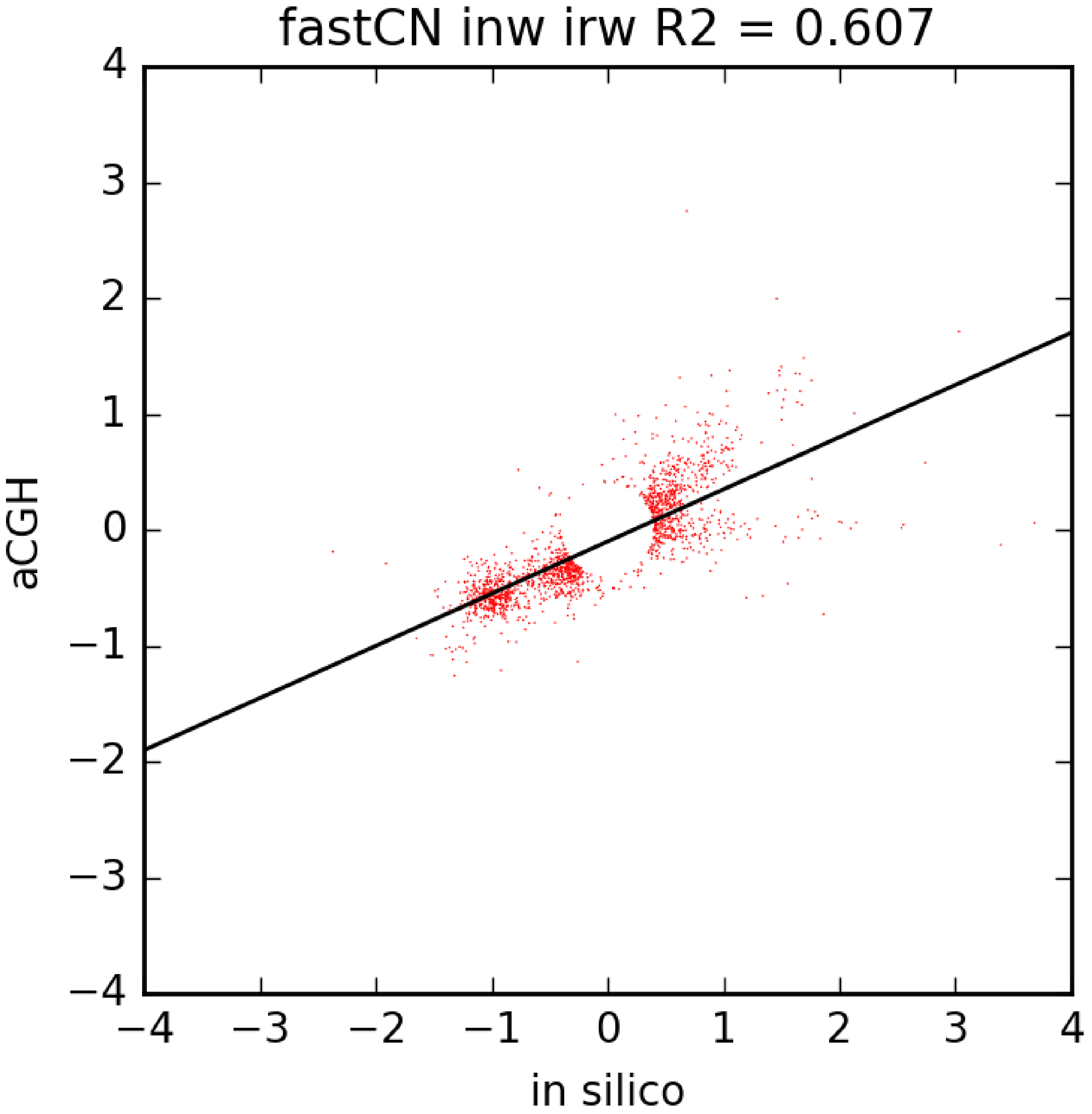
fastCN inw irw R2 = 0.607
aCGH
in silico
4
3
2
1
0
−1
−2
−3
−4
−4
−3
−2
−1
0
1
2
3
4

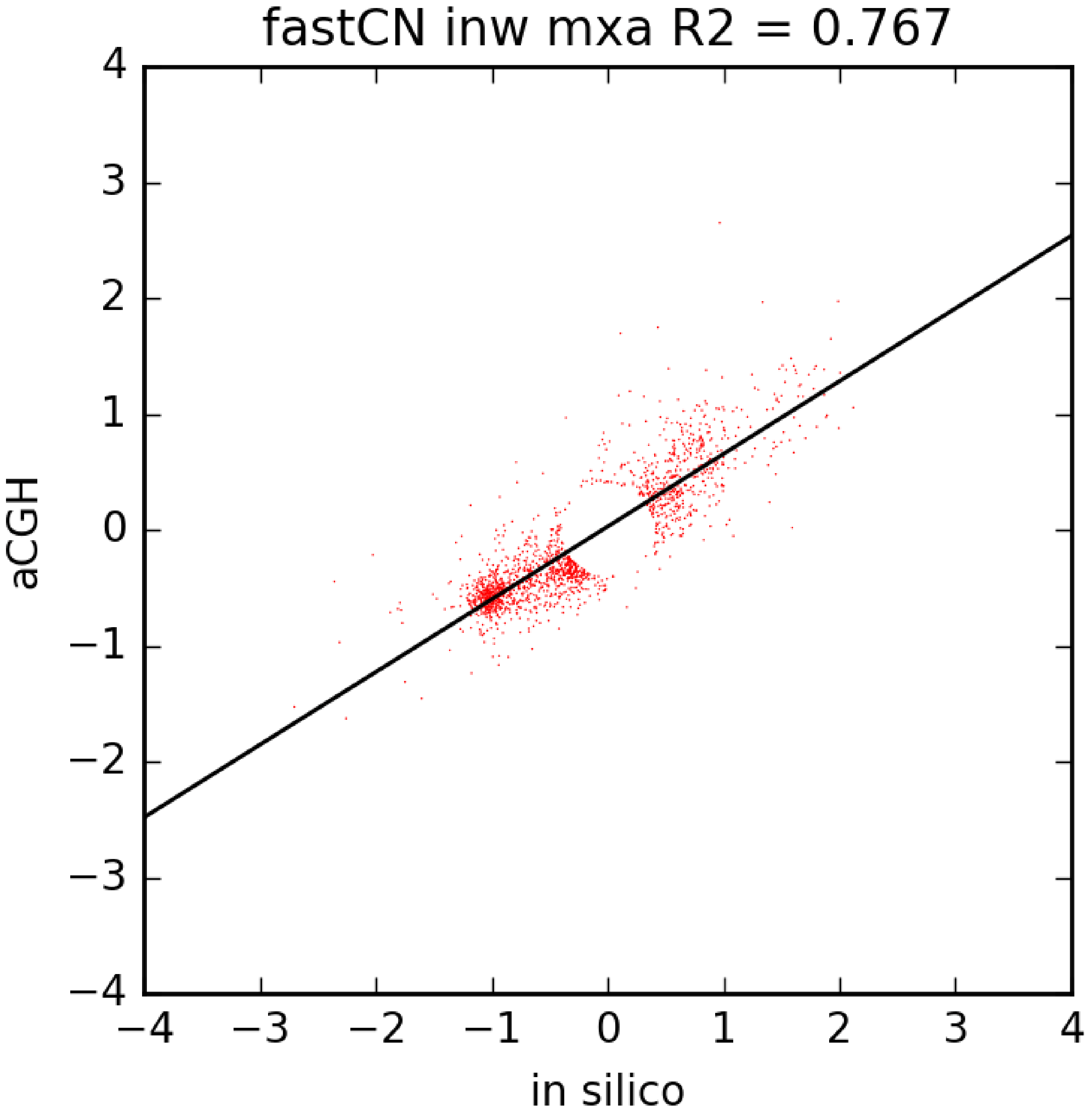
fastCN inw mxa R2 = 0.767
aCGH
in silico
4
3
2
1
0
−1
−2
−3
−4
−4
−3
−2
−1
0
1
2
3
4

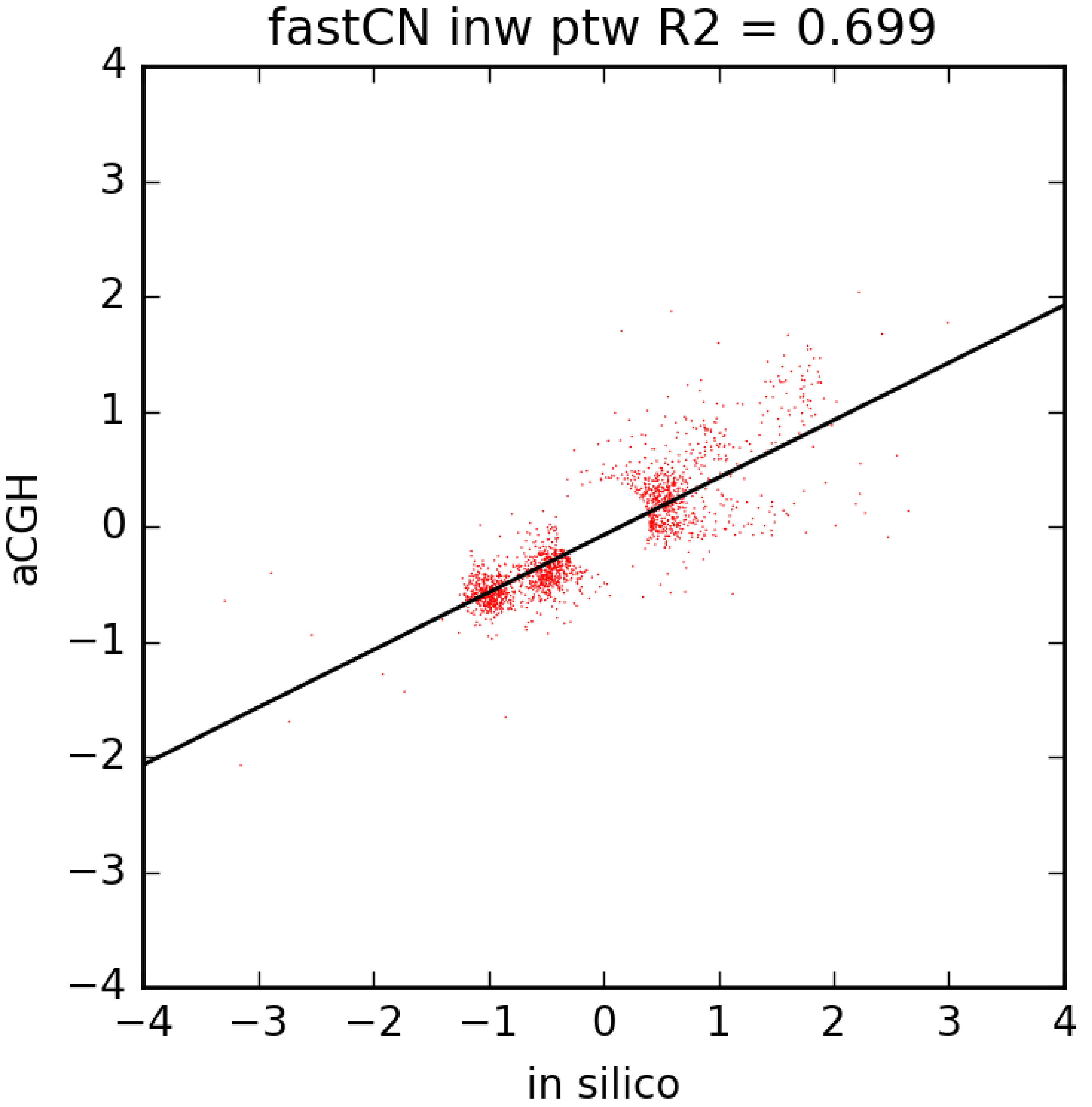

fastCN inw ptw R2 = 0.699
aCGH
in silico
−4
−3
−2
−1
0
1
2
3
4

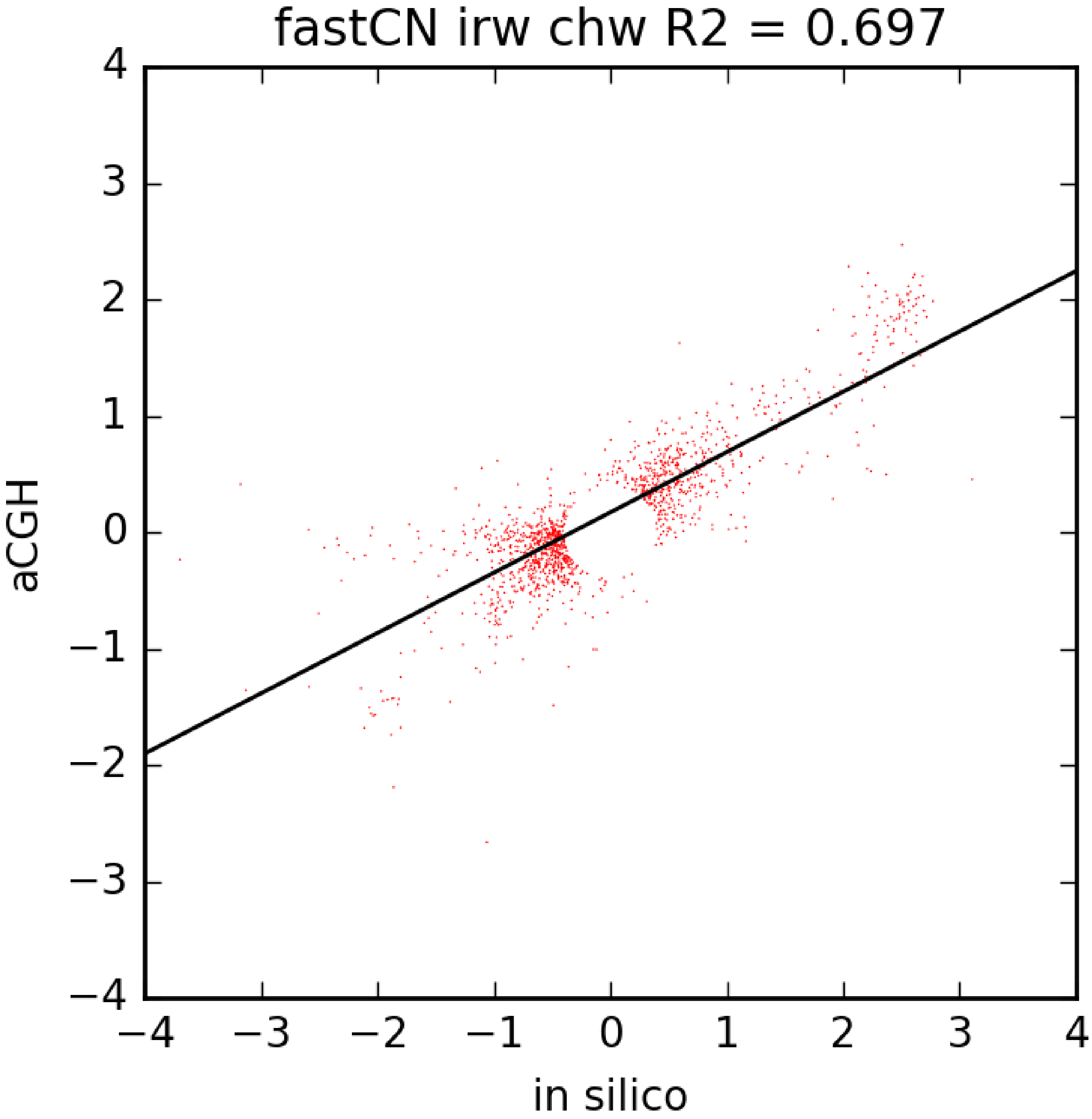

fastCN irw chw R2 = 0.697
aCGH
in silico
4
3
2
1
0
−1
−2
−3
−4
−4
−3
−2
−1
0
1
2
3
4

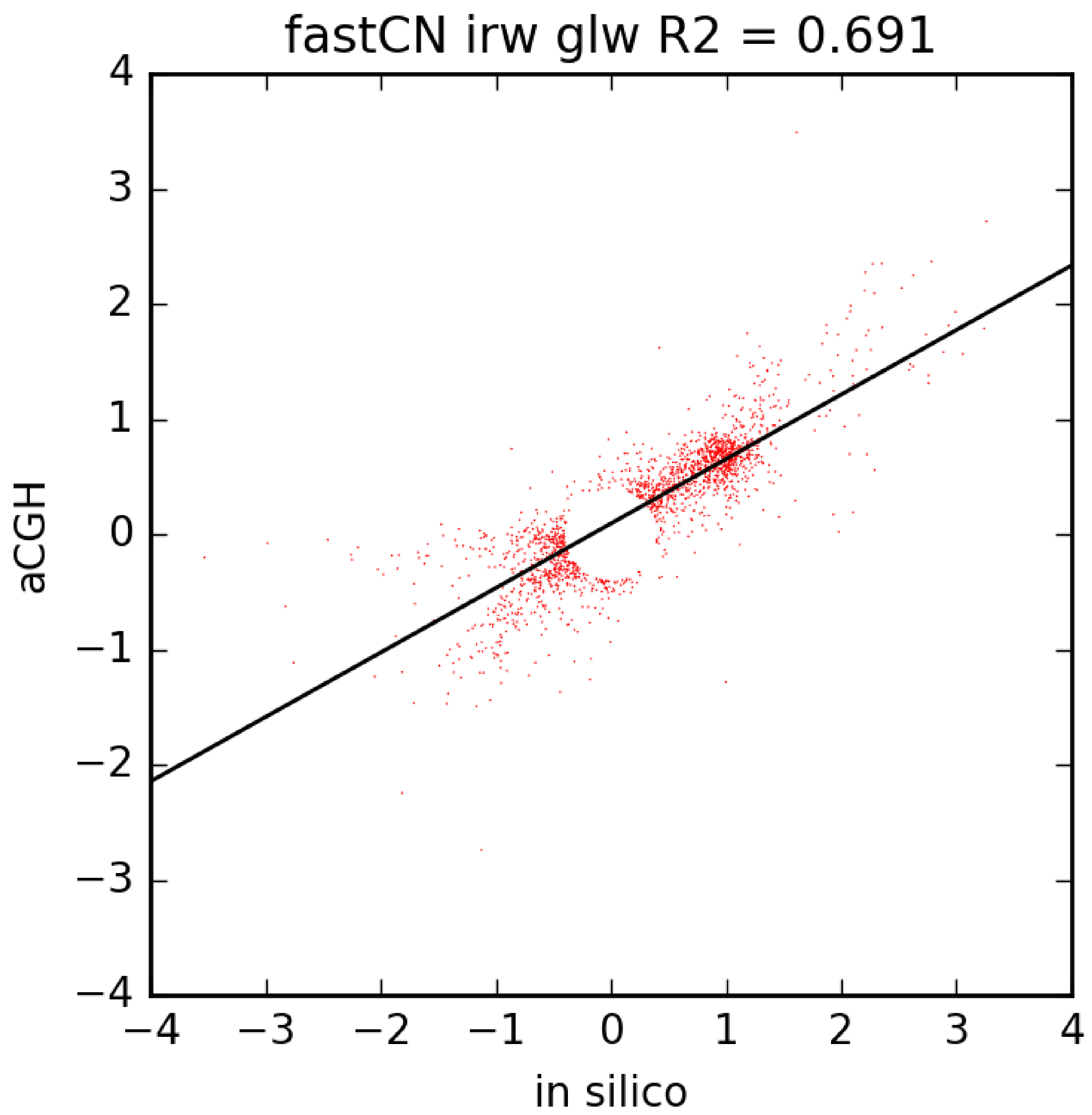

fastCN irw glw R2 = 0.691
aCGH
in silico
4
3
2
1
0
−1
−2
−3
−4
−4
−3
−2
−1
0
1
2
3
4

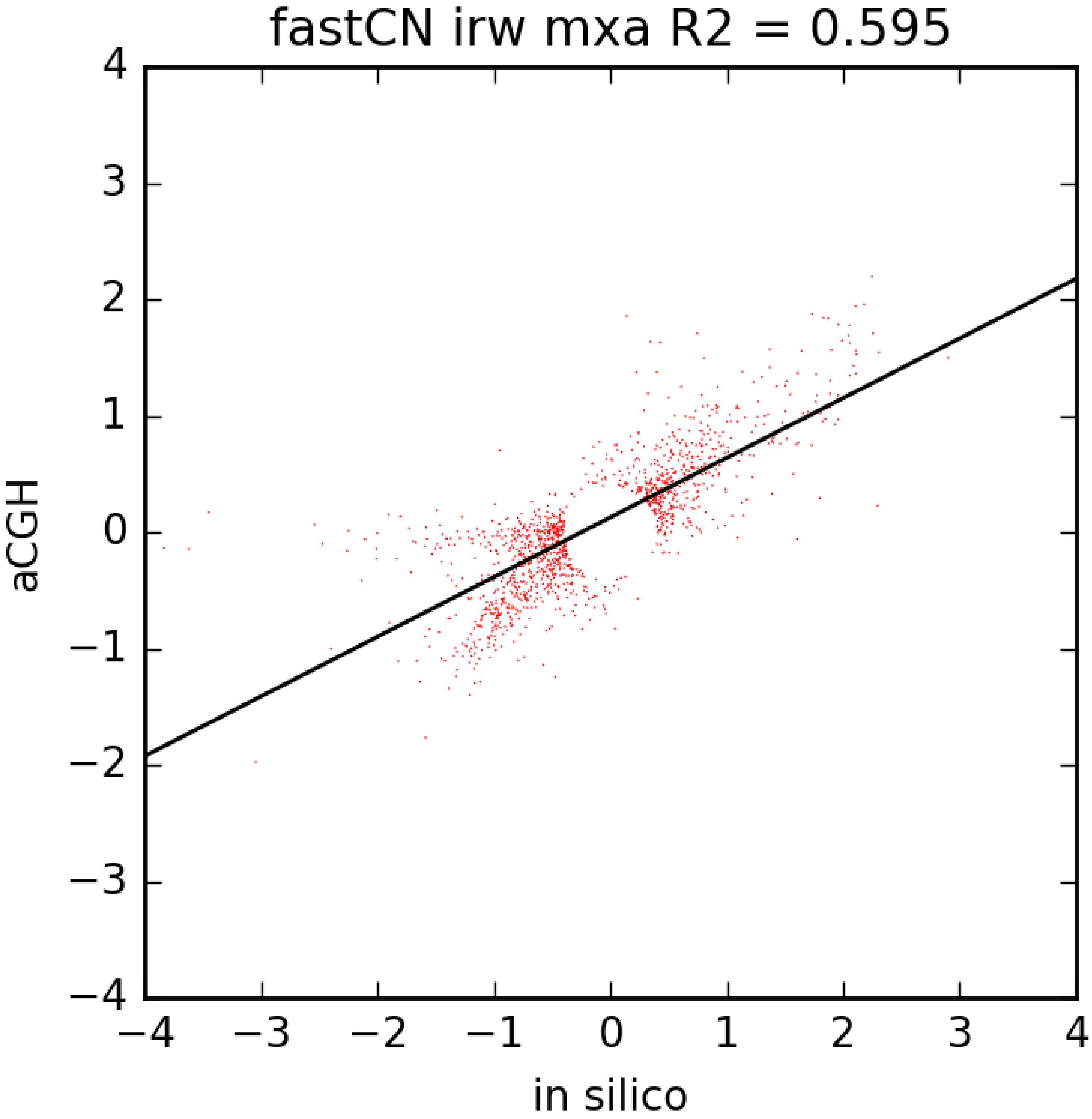
fastCN irw mxa R2 = 0.595
aCGH
in silico
4
3
2
1
0
−1
−2
−3
−4
−4
−3
−2
−1
0
1
2
3
4

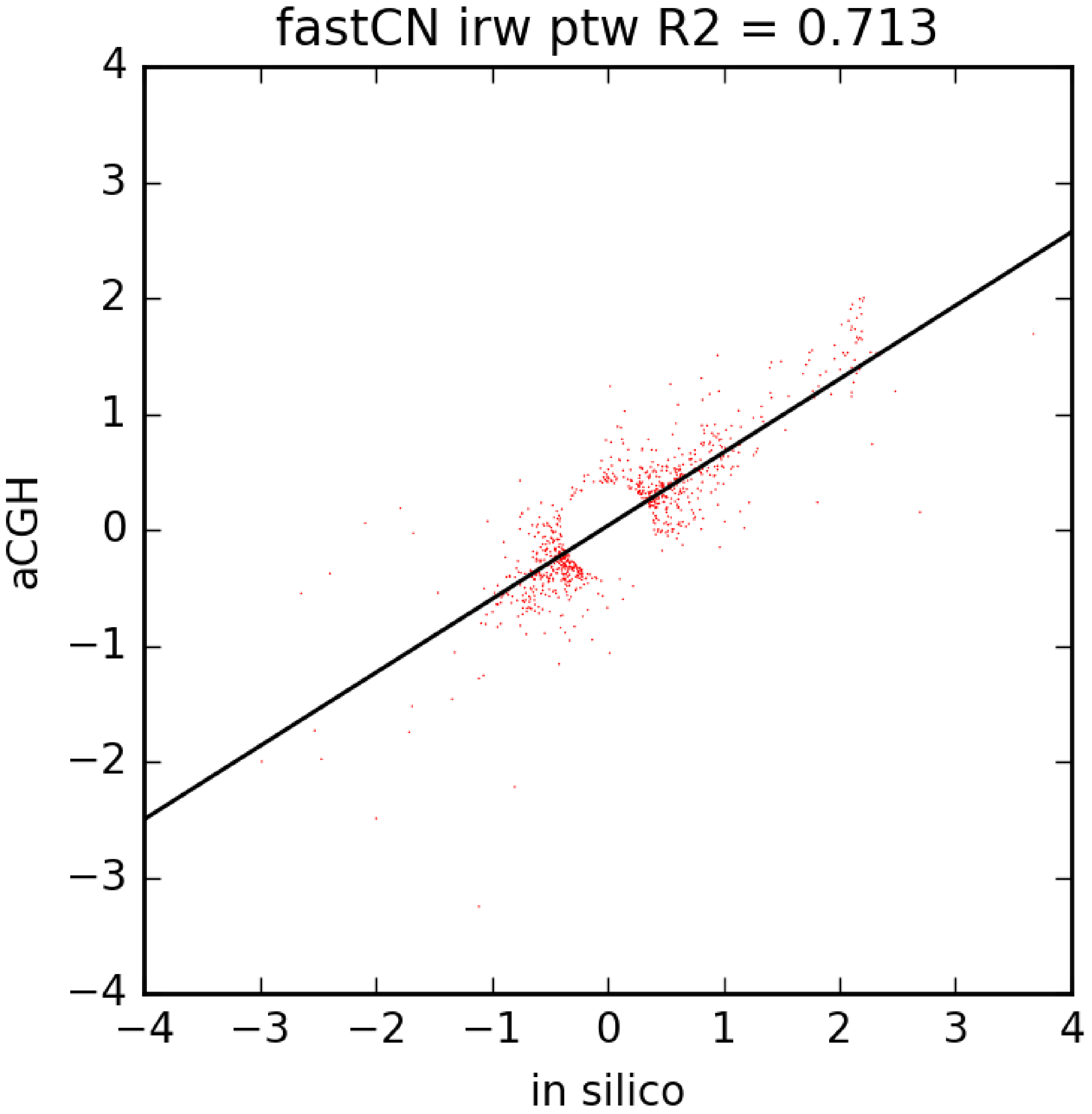
fastCN irw ptw R2 = 0.713
aCGH
in silico
4
3
2
1
0
−1
−2
−3
−4
−4
−3
−2
−1
0
1
2
3
4

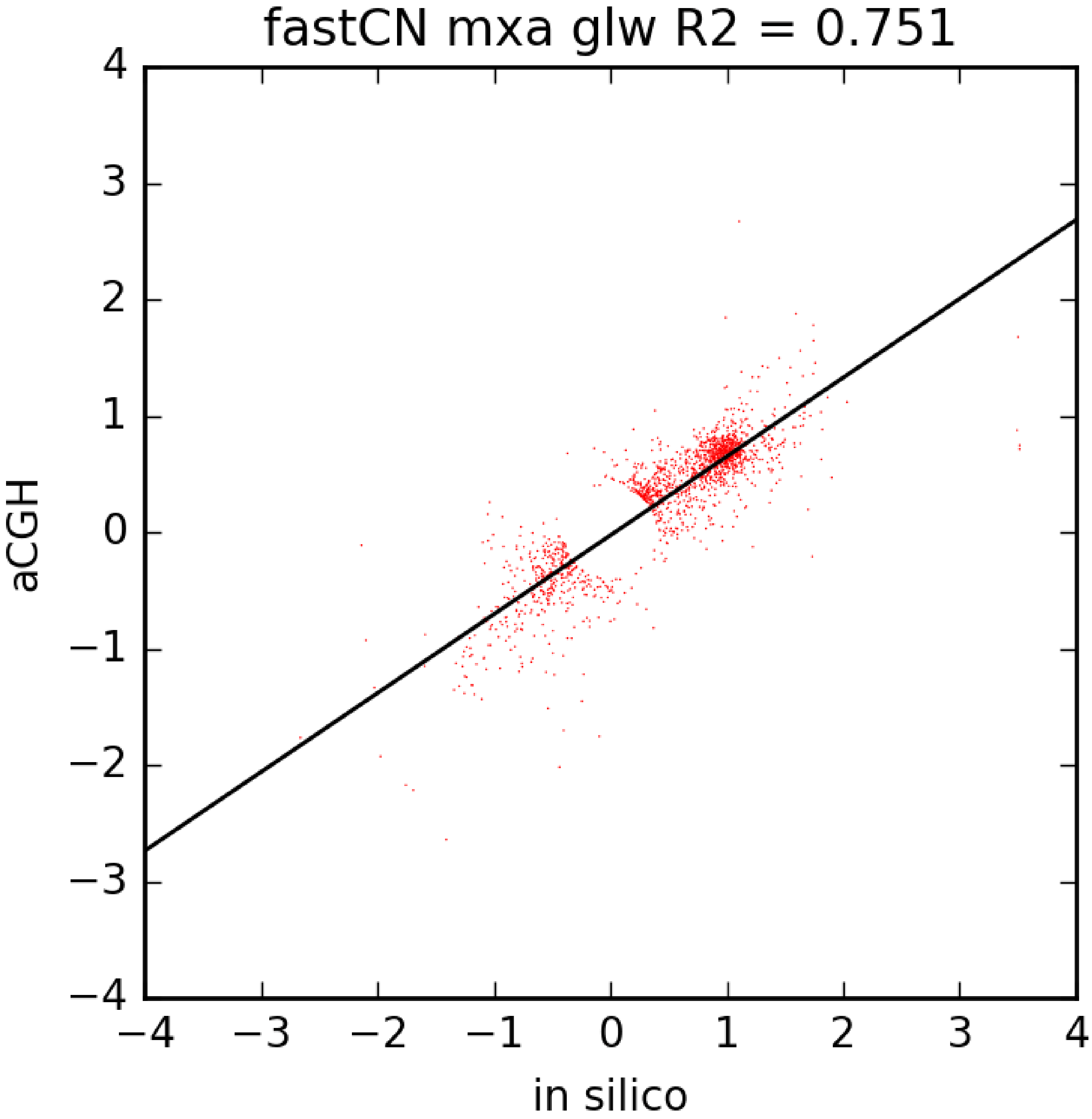

fastCN mxa glw R2 = 0.751
aCGH
in silico
4
3
2
1
0
−1
−2
−3
−4
−4
−3
−2
−1
0
1
2
3
4

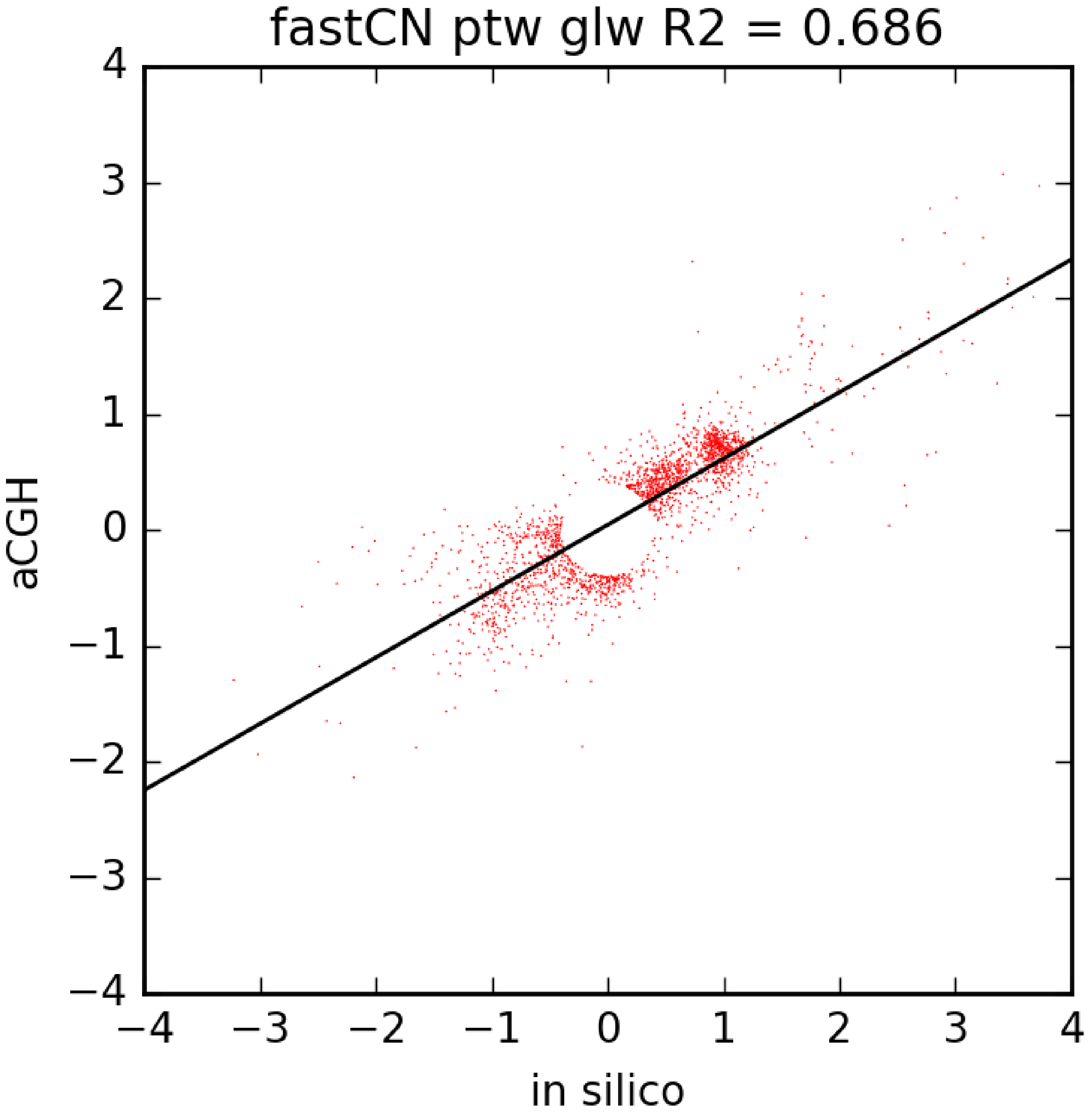
fastCN ptw glw R2 = 0.686
aCGH
in silico
4
3
2
1
0
−1
−2
−3
−4
−4
−3
−2
−1
0
1
2
3
4

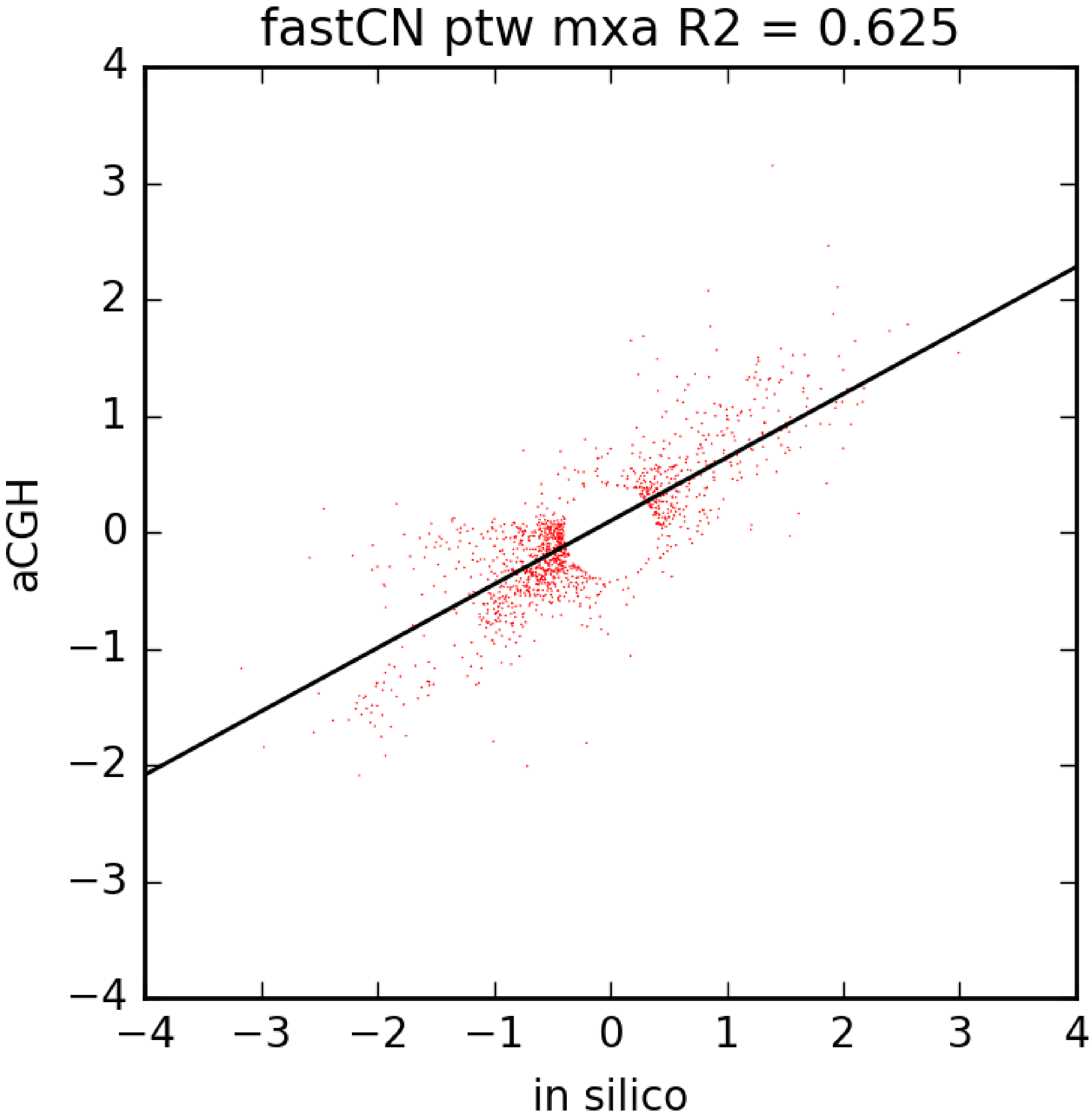

fastCN ptw mxa R2 = 0.625
aCGH
in silico
4
3
2
1
0
−1
−2
−3
−4

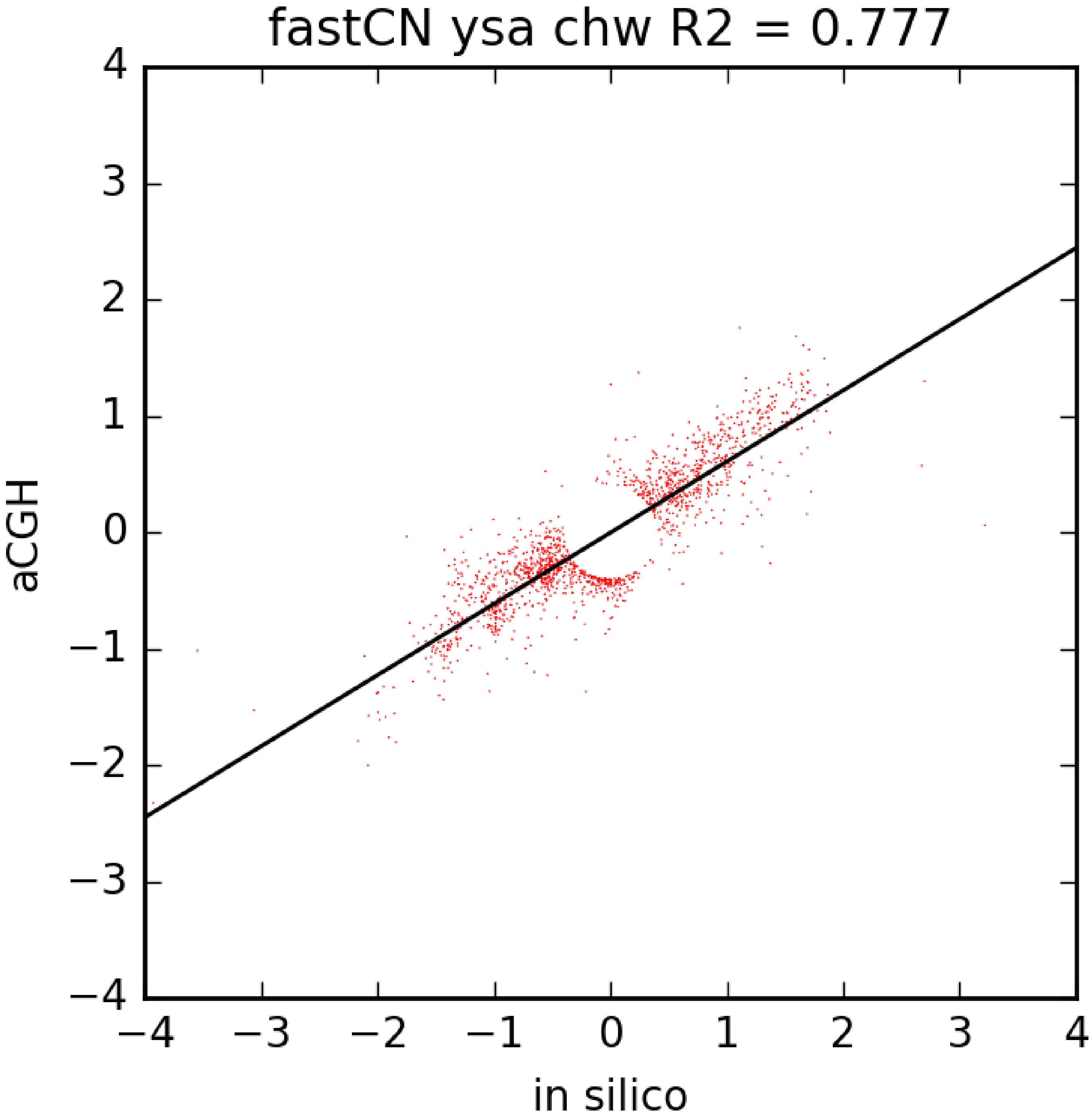
fastCN ysa chw R2 = 0.777
aCGH
in silico
4
3
2
1
0
−1
−2
−3
−4
−4
−3
−2
−1
0
1
2
3
4

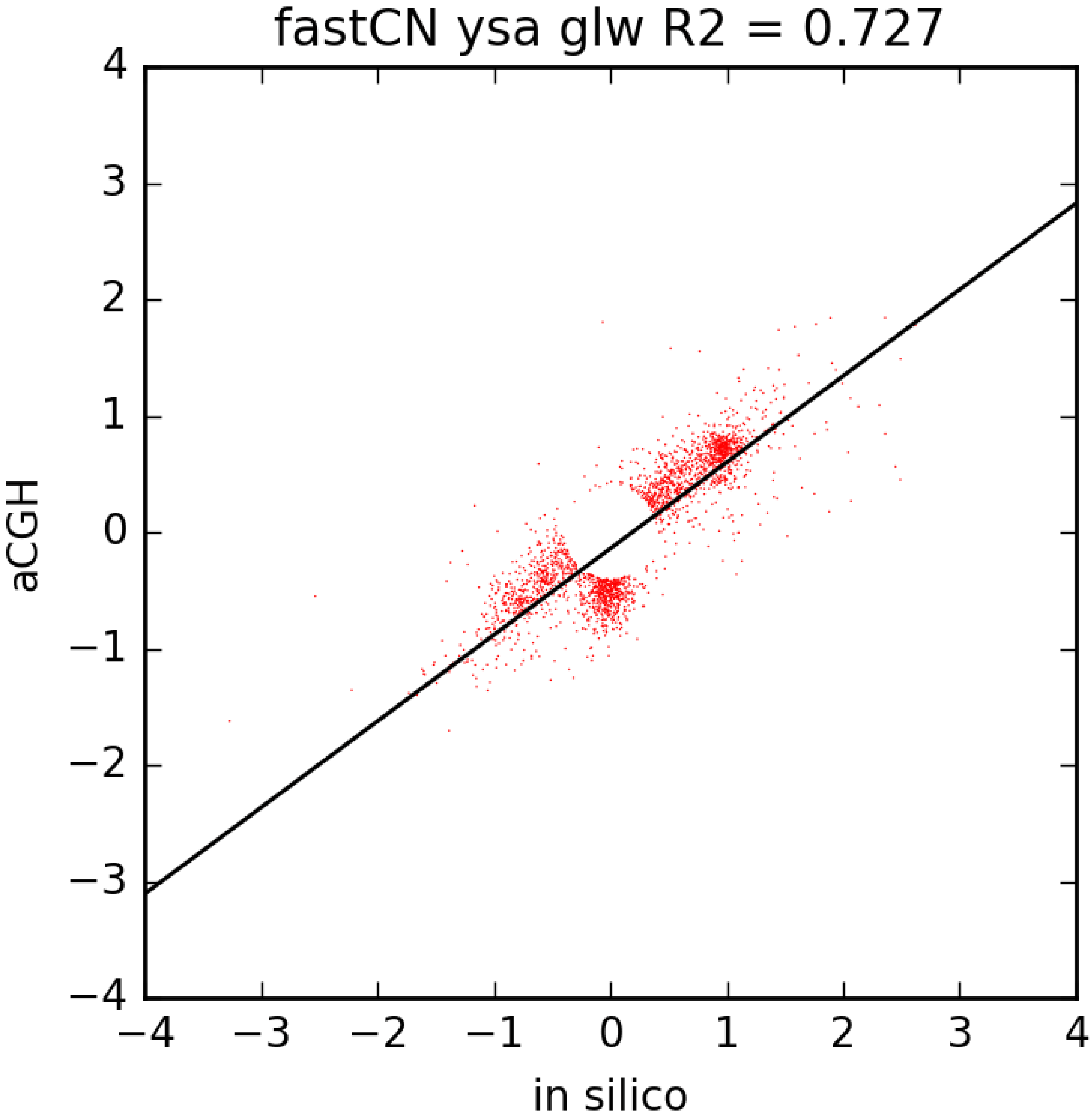

fastCN ysa glw R2 = 0.727
aCGH
in silico
4
3
2
1
0
−1
−2
−3
−4

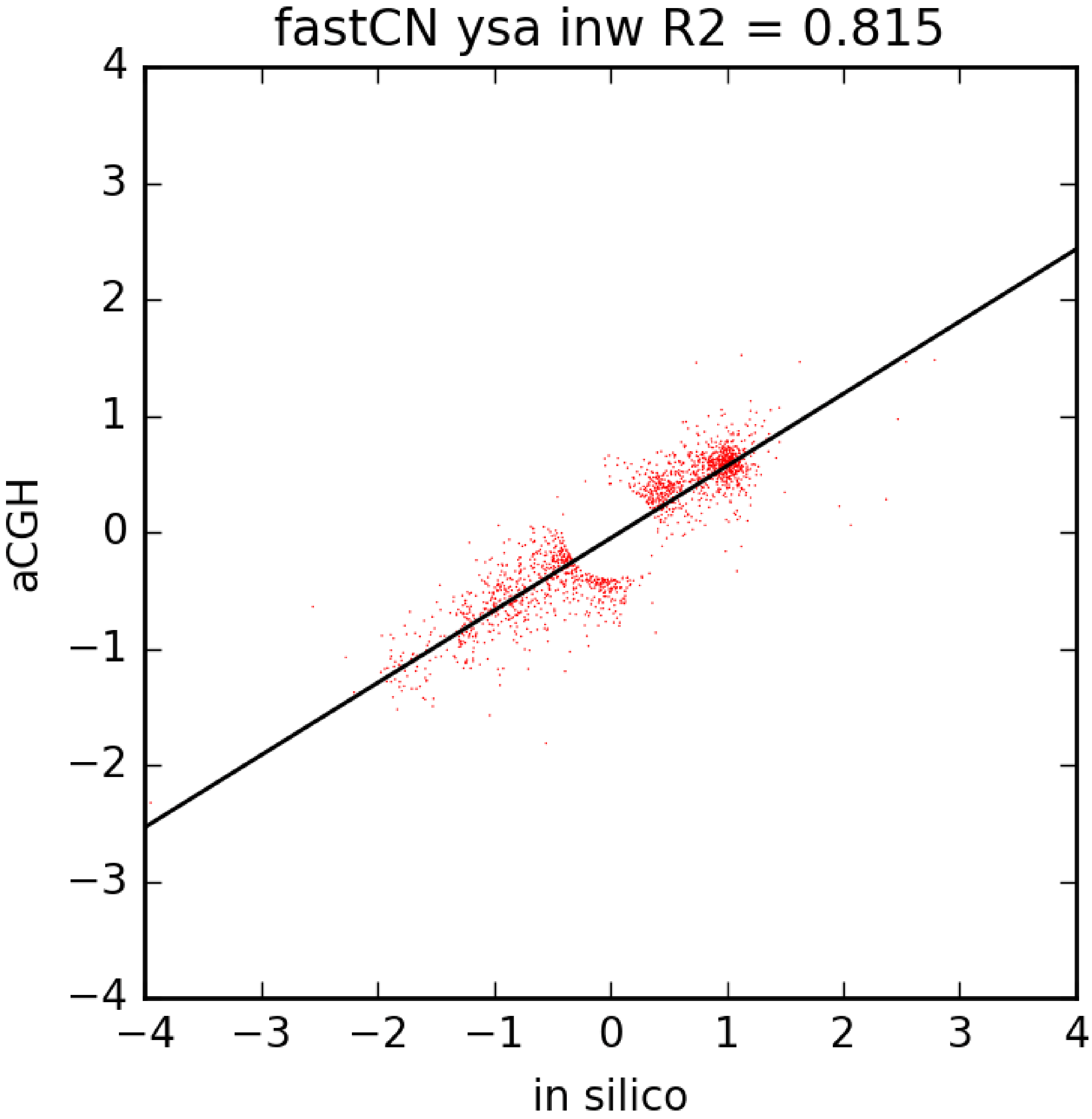
fastCN ysa inw R2 = 0.815
aCGH
in silico
4
3
2
1
0
−1
−2
−3
−4
−4
−3
−2
−1
0
1
2
3
4

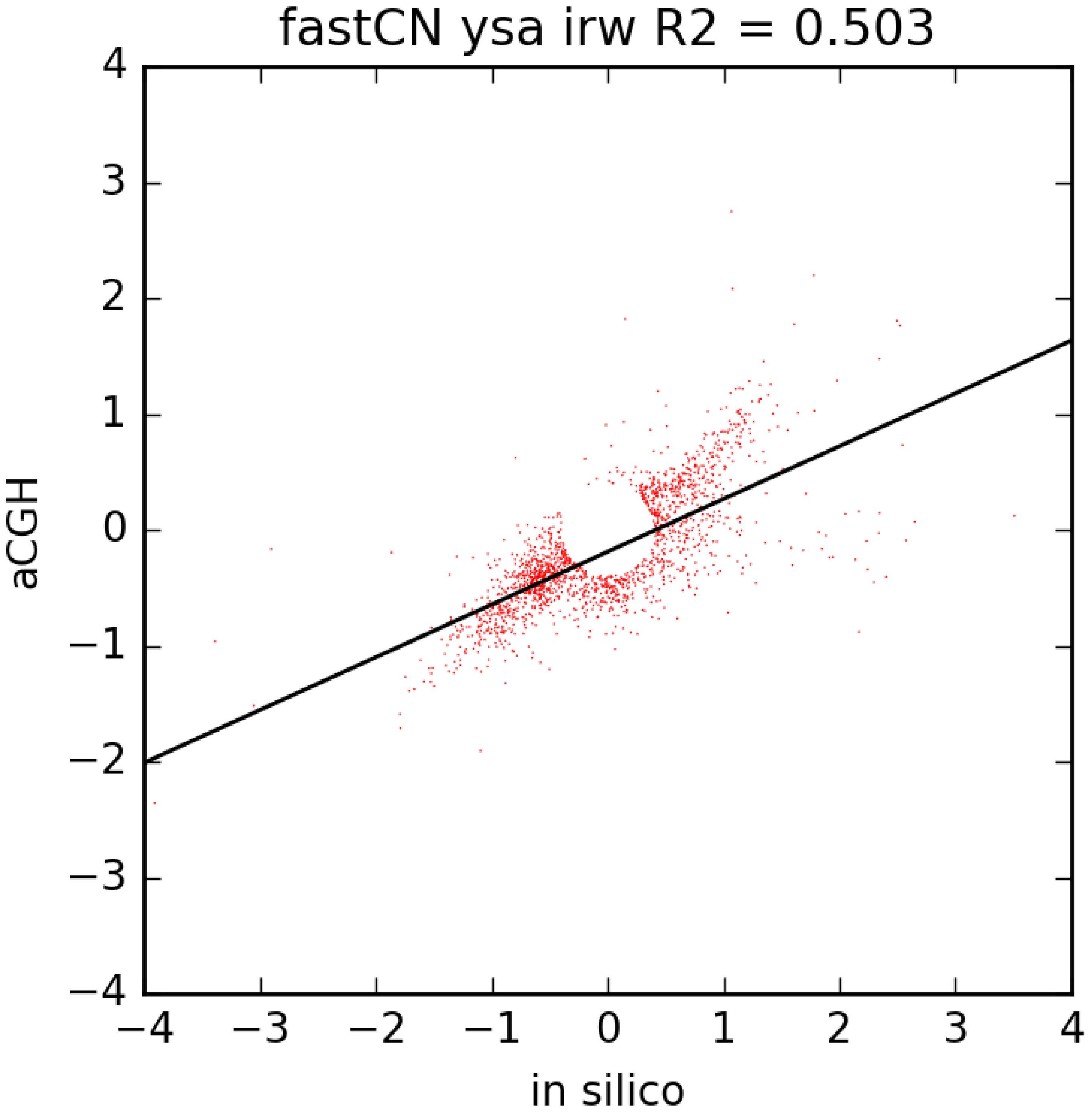
fastCN ysa irw R2 = 0.503
aCGH
in silico
4
3
2
1
0
−1
−2
−3
−4
−4
−3
−2
−1
0
1
2
3
4

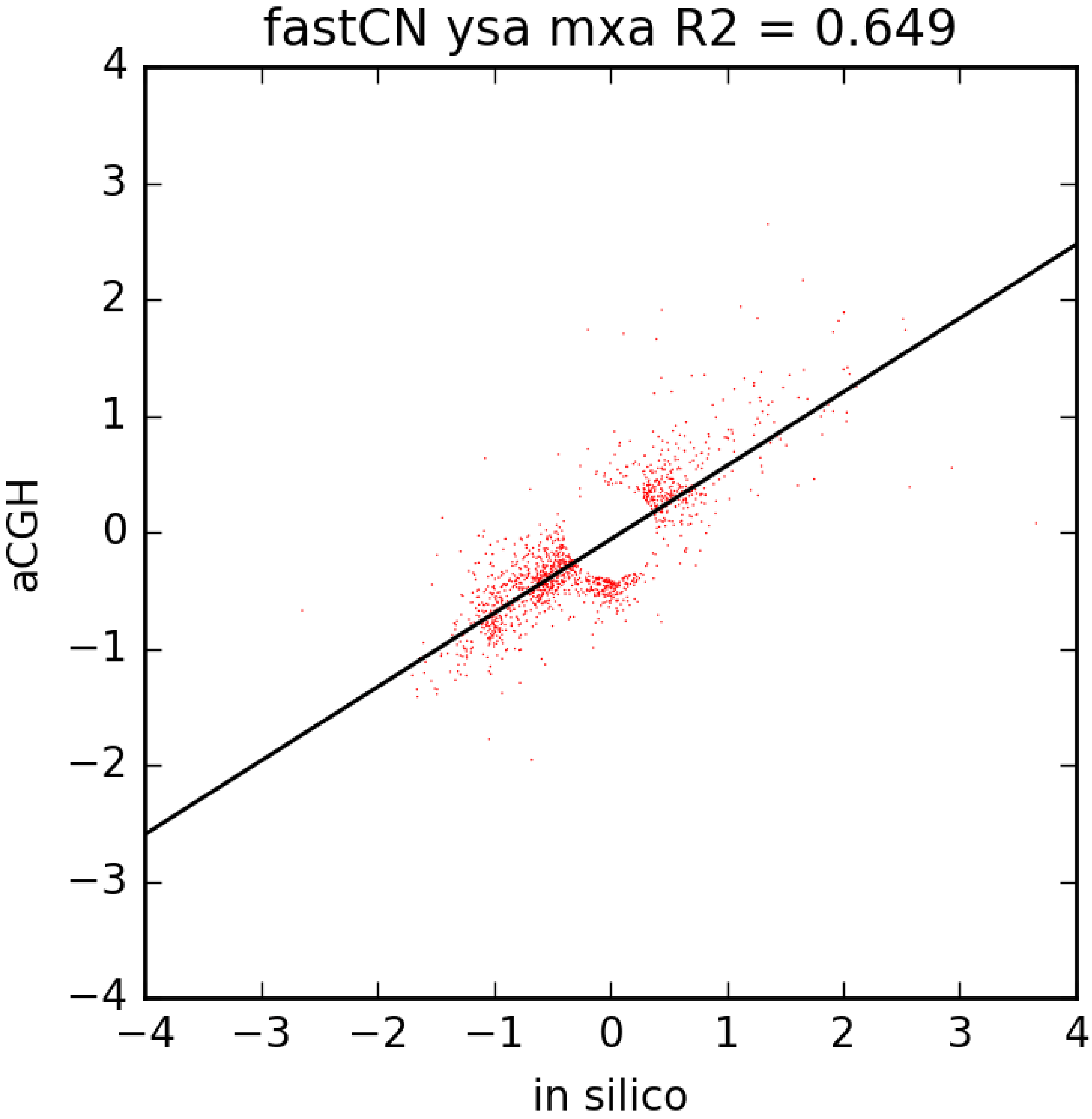

fastCN ysa mxa R2 = 0.649
4
3
2
1
0
−1
−2
−3
−4
aCGH
−4
−3
−2
−1
0
1
2
3
4
in silico

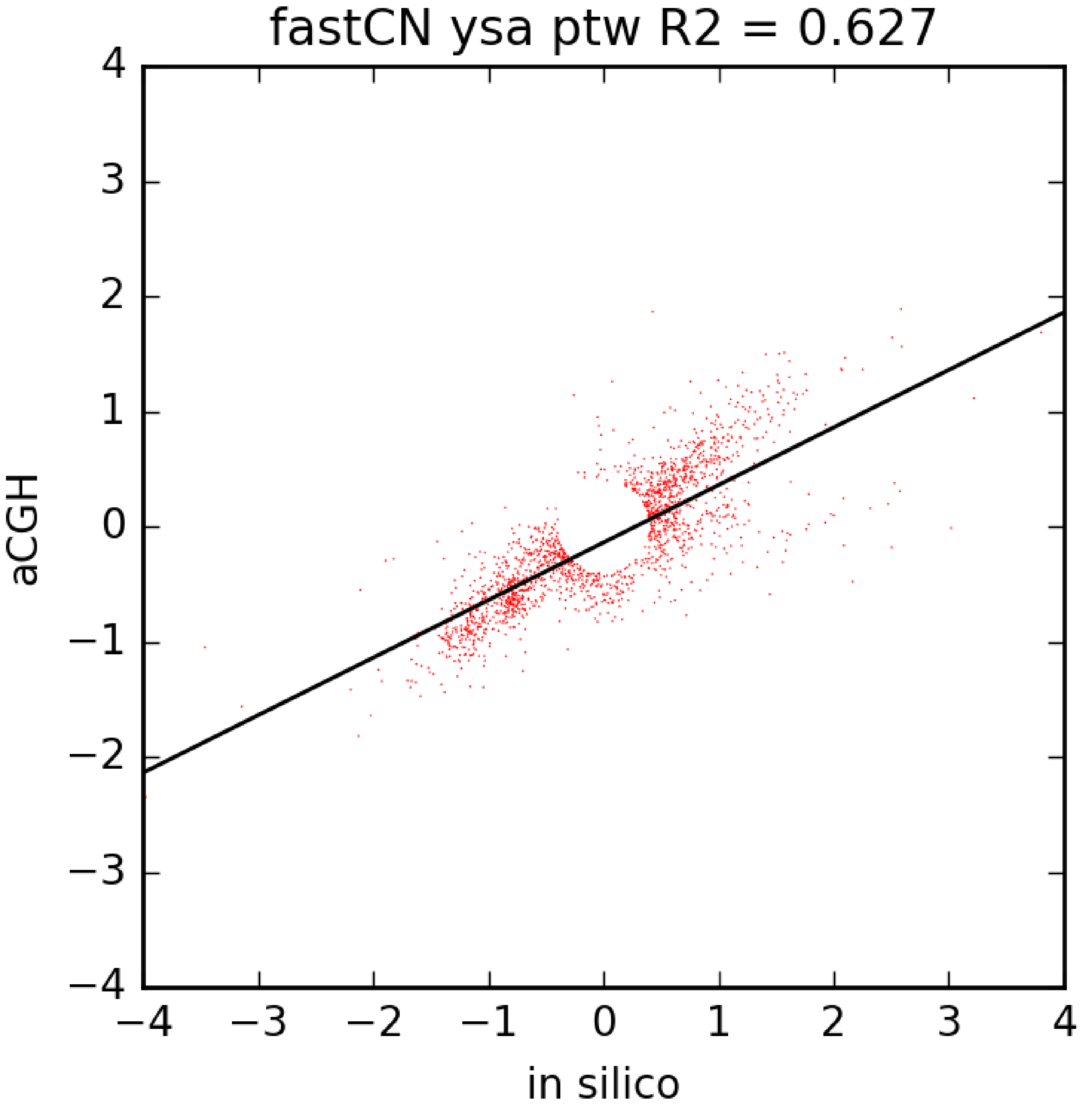
fastCN ysa ptw R2 = 0.627
aCGH
in silico
4
3
2
1
0
−1
−2
−3
−4
−4
−3
−2
−1
0
1
2
3
4

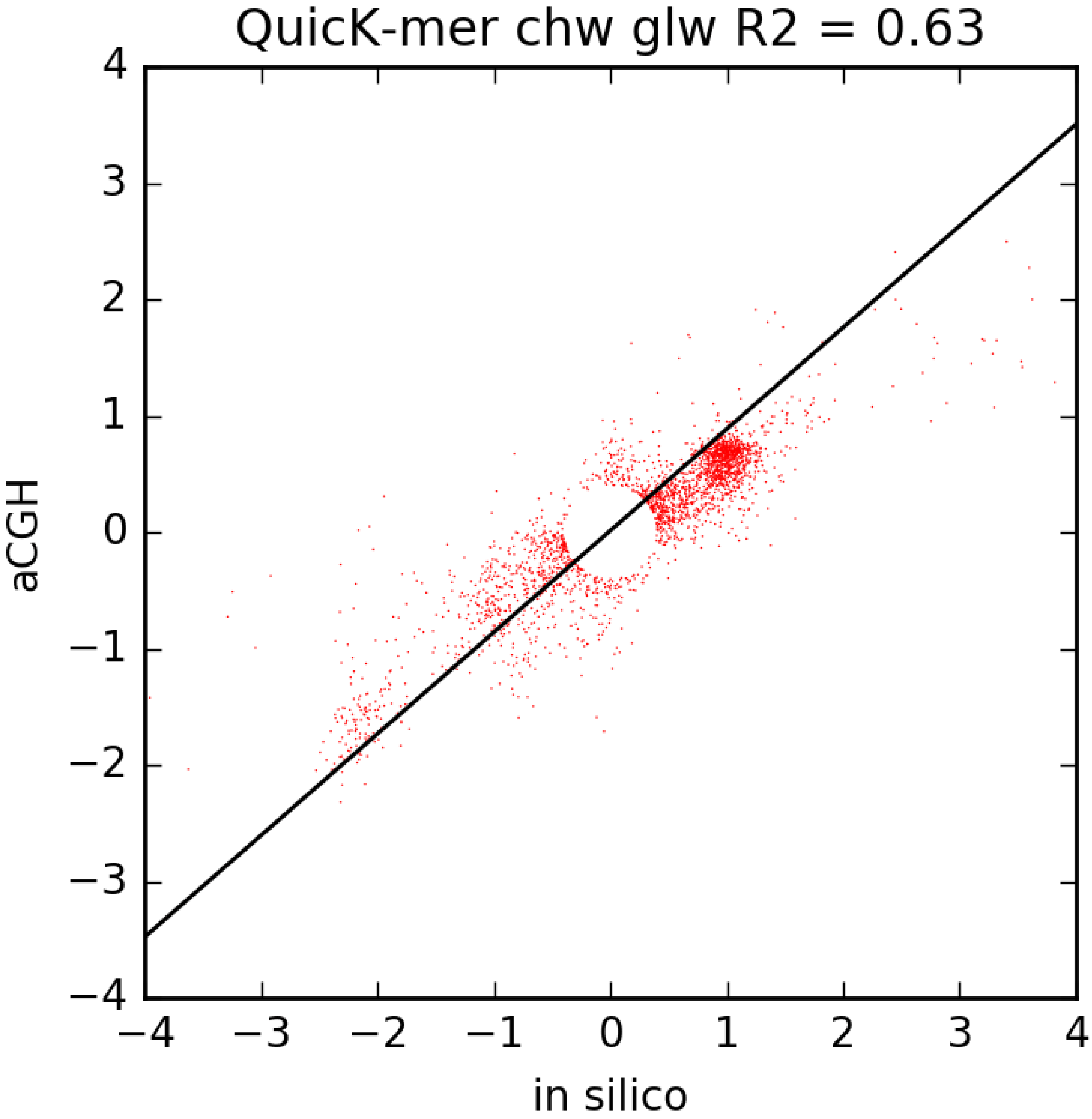
QuicK-mer chw glw R2 = 0.63
aCGH
in silico
4
3
2
1
0
−1
−2
−3
−4
−4
−3
−2
−1
0
1
2
3
4

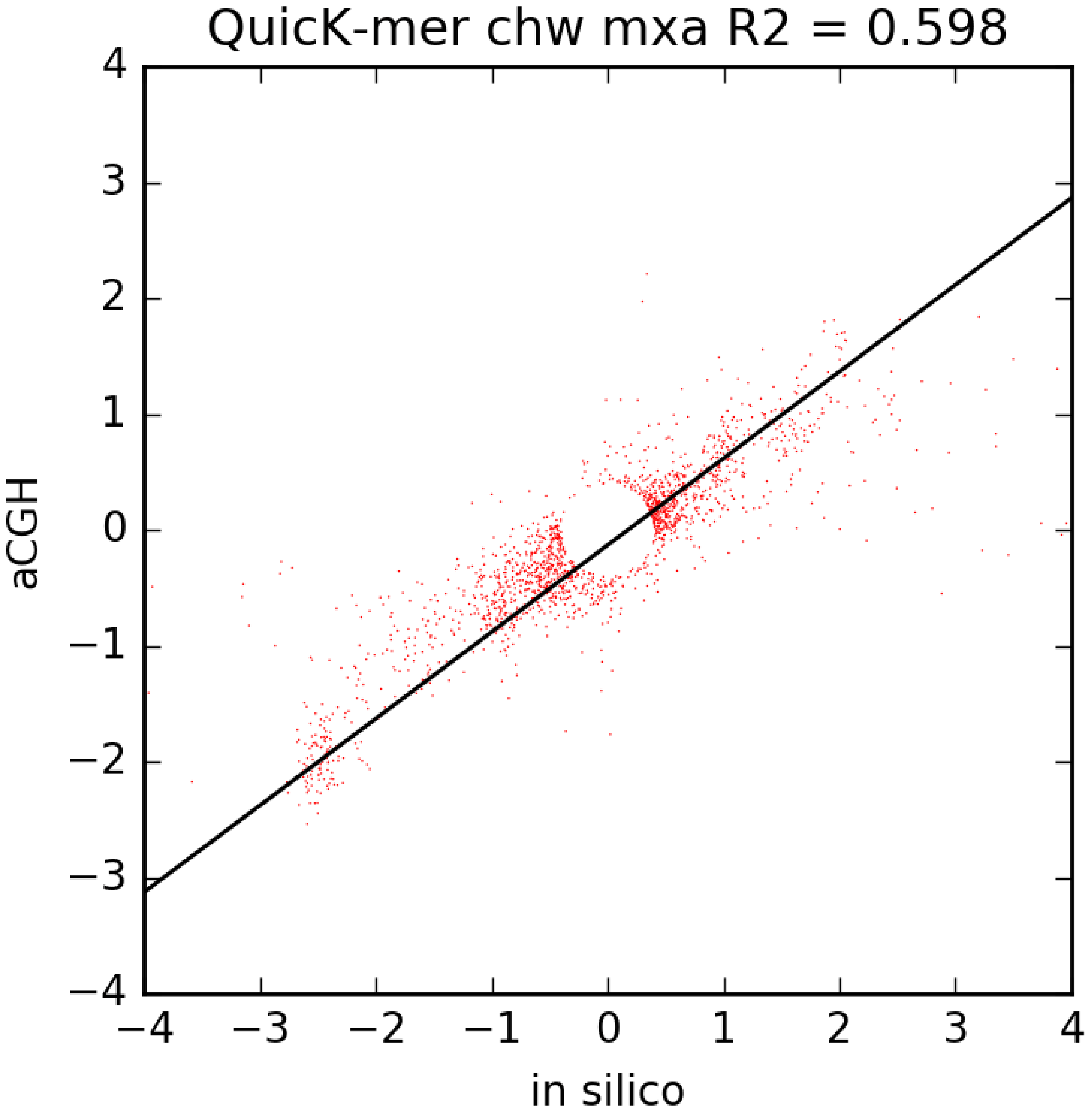
QuicK-mer chw mxa R2 = 0.598
aCGH
in silico
4
3
2
1
0
−1
−2
−3
−4
−4
−3
−2
−1
0
1
2
3
4

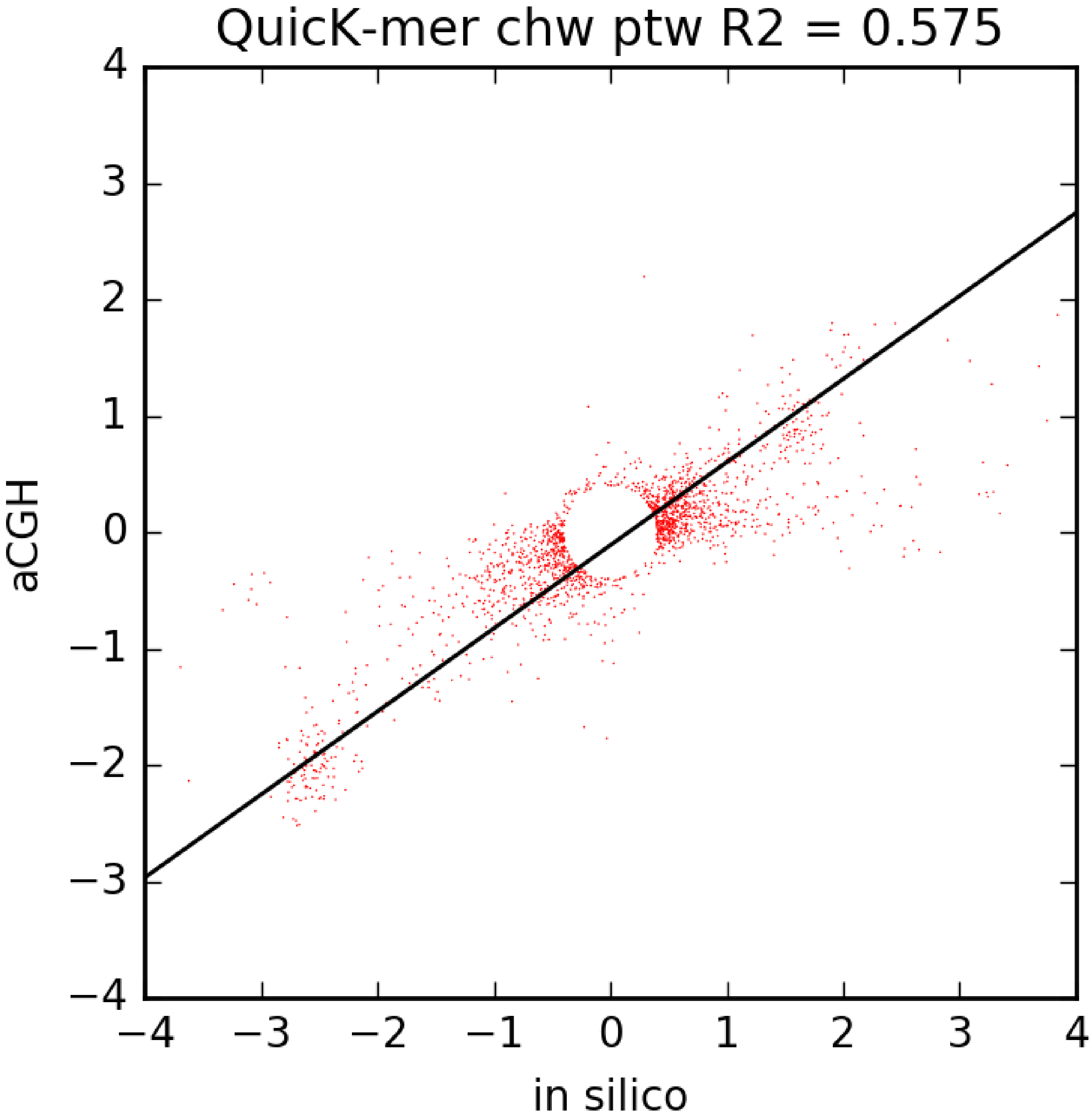
QuicK-mer chw ptw R2 = 0.575
aCGH
in silico
4
3
2
1
0
−1
−2
−3
−4
−4
−3
−2
−1
0
1
2
3
4

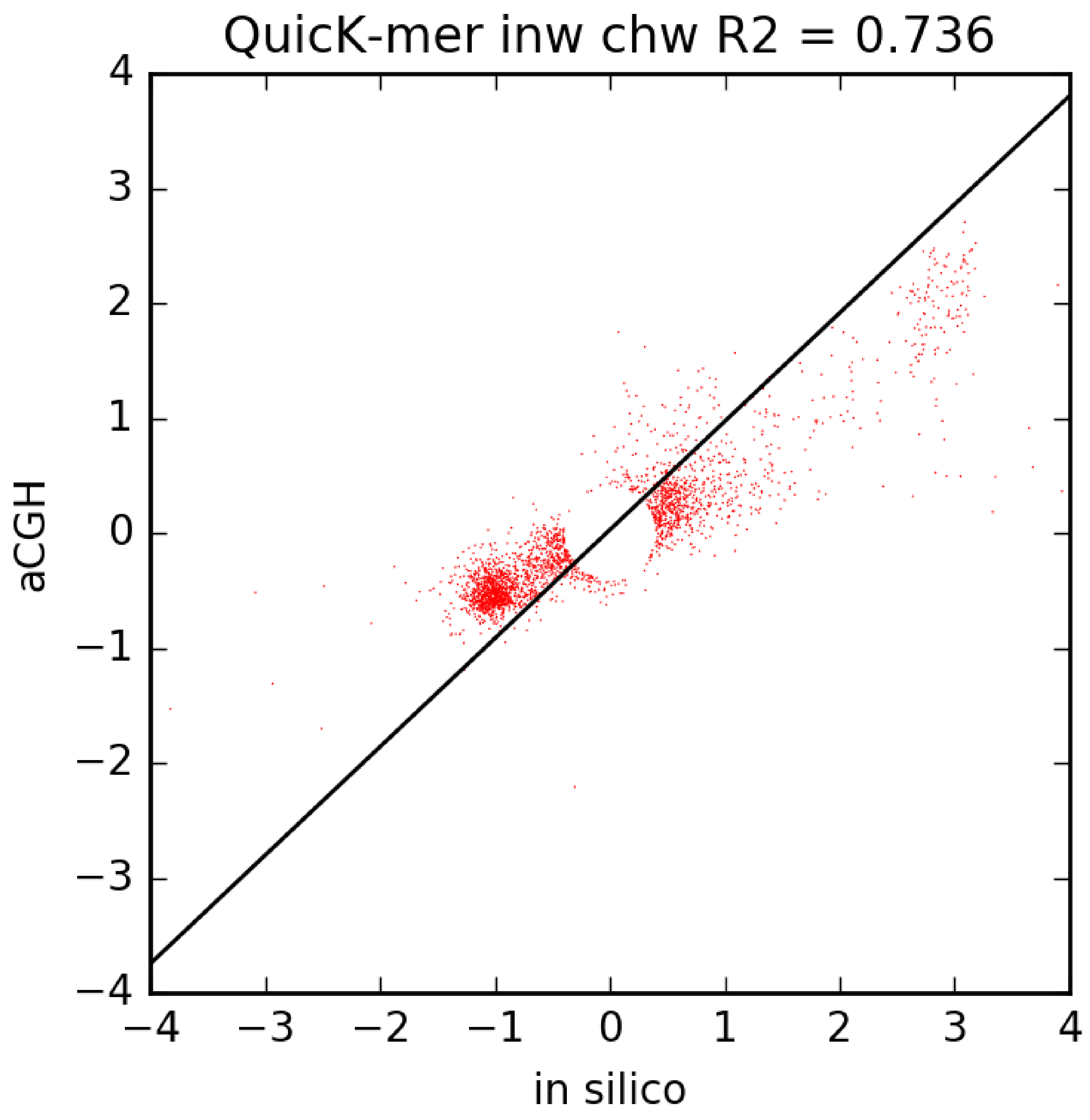
QuicK-mer inw chw R2 = 0.736
aCGH
in silico
4
3
2
1
0
−1
−2
−3
−4
−4
−3
−2
−1
0
1
2
3
4

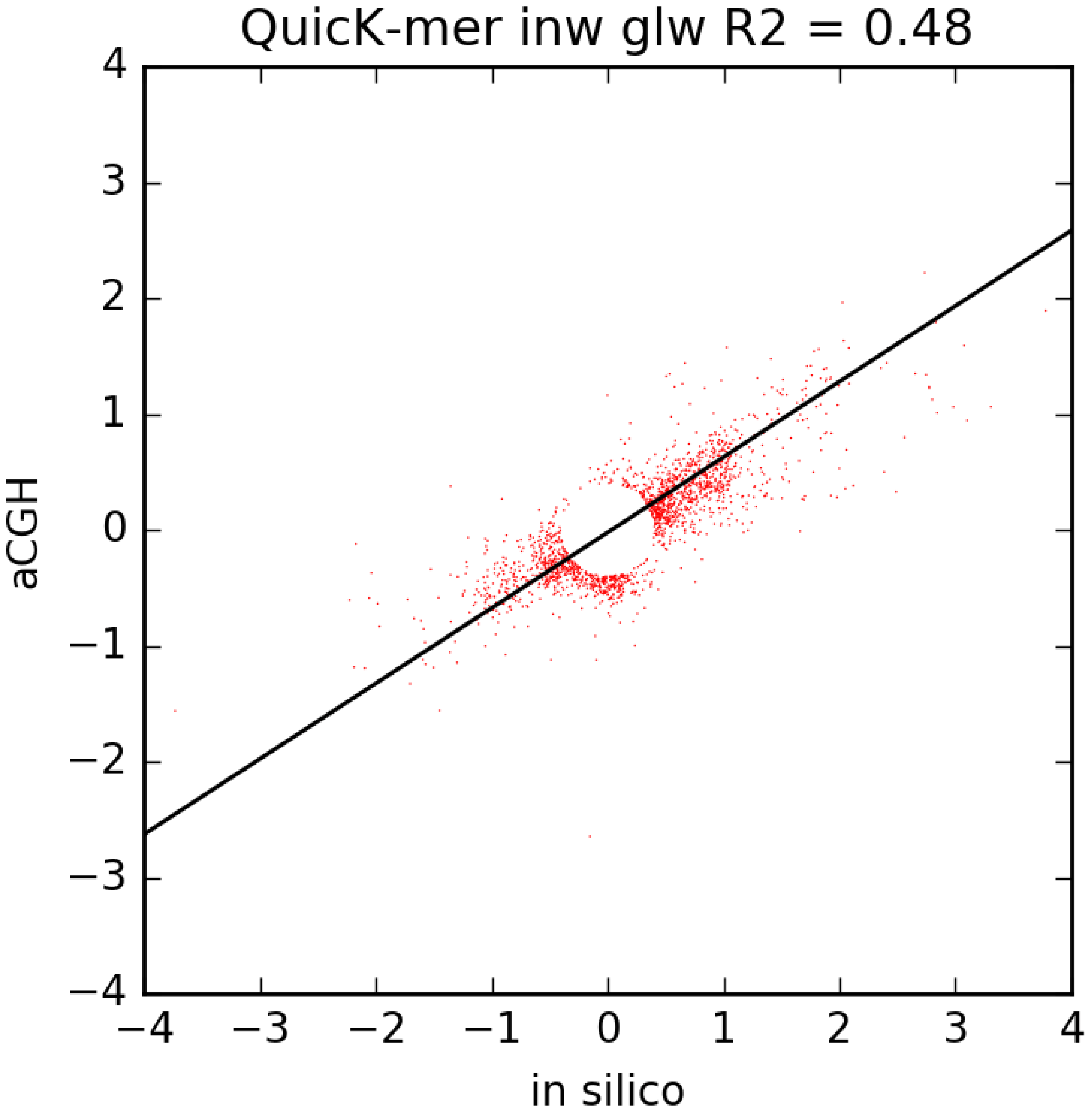

QuicK-mer inw glw R2 = 0.48
aCGH
in silico
4
3
2
1
0
−1
−2
−3
−4
−4
−3
−2
−1
0
1
2
3
4

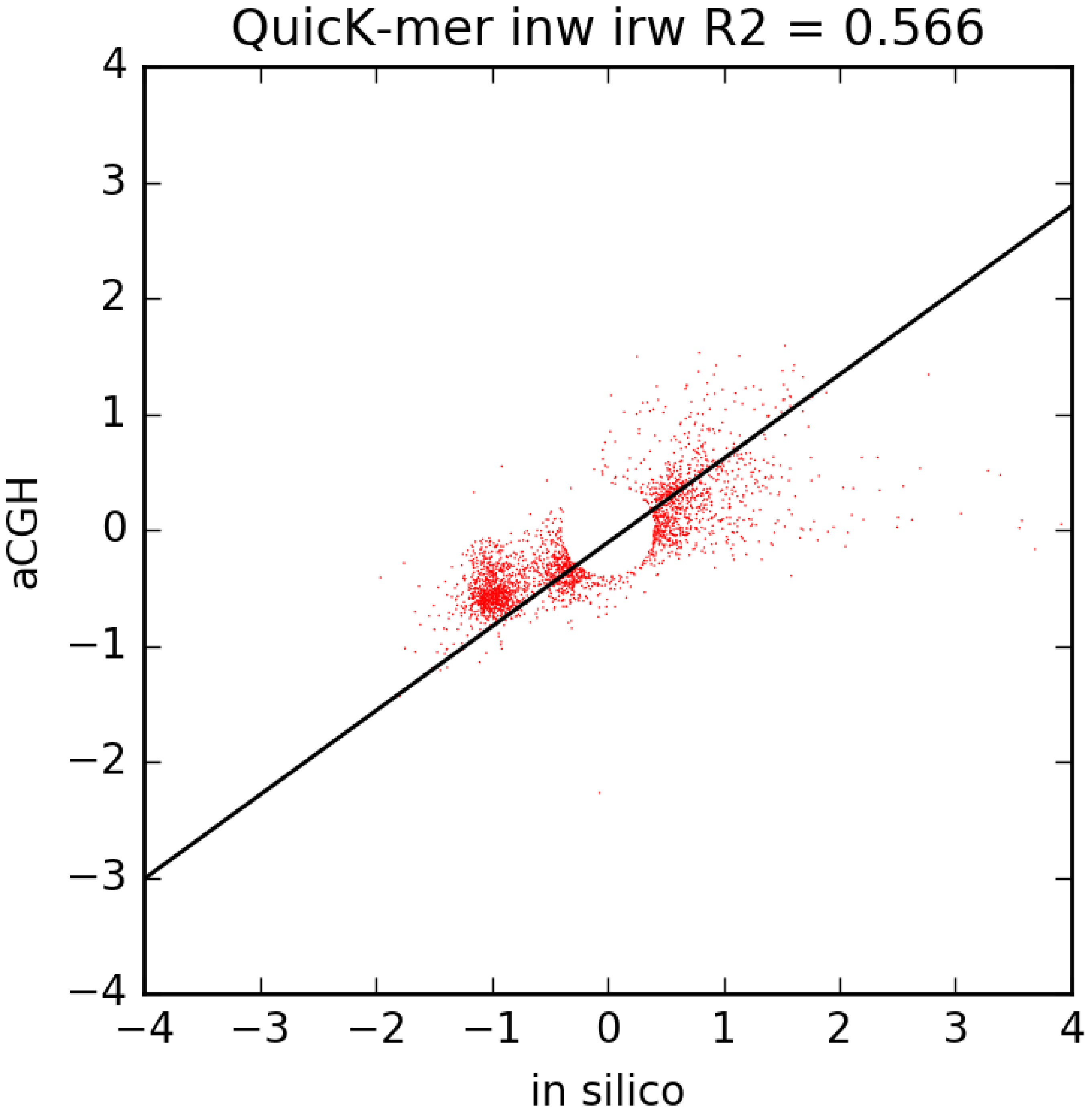
QuicK-mer inw irw R2 = 0.566
aCGH
in silico
4
3
2
1
0
−1
−2
−3
−4
−4
−3
−2
−1
0
1
2
3
4

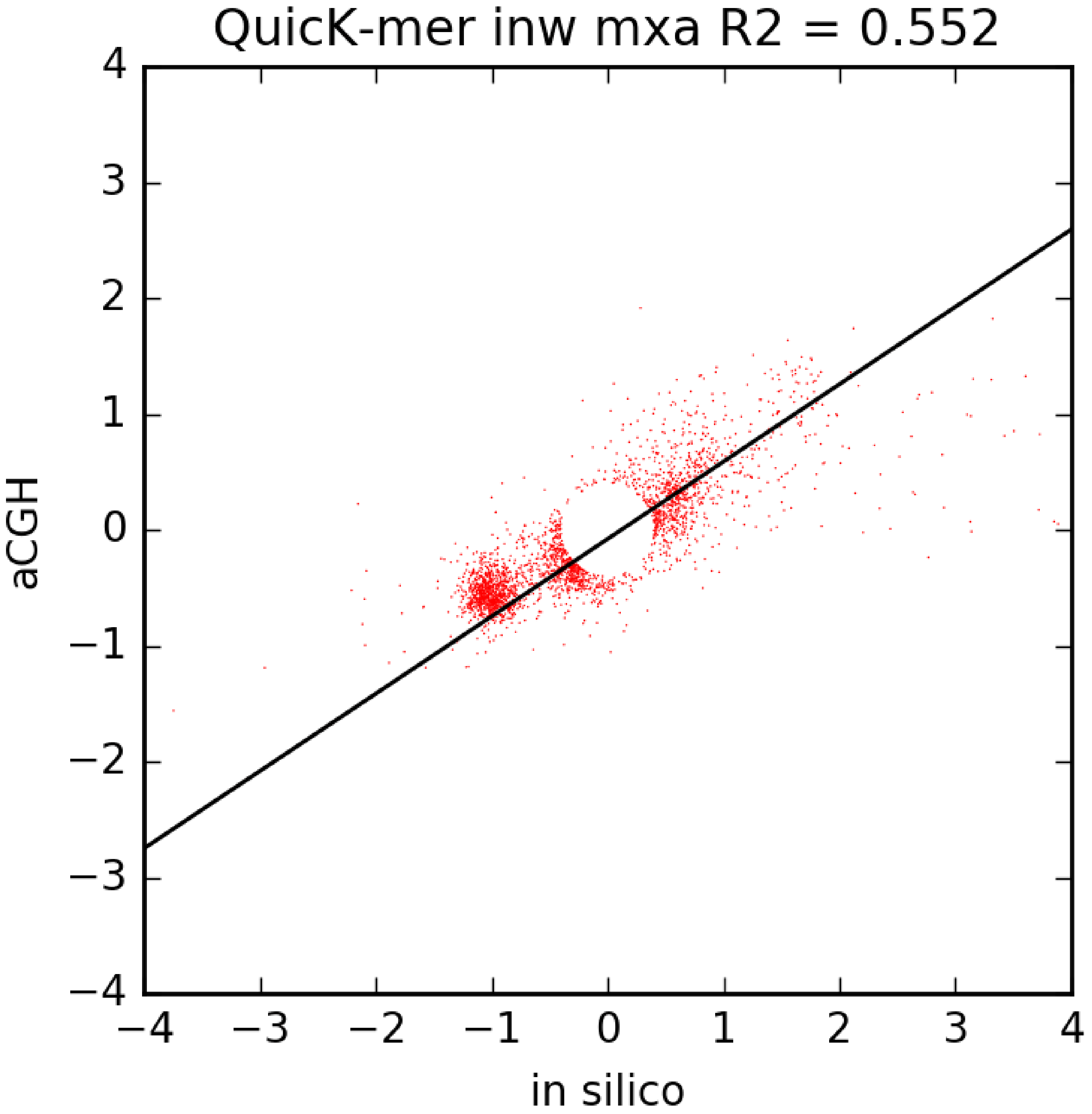
QuicK-mer inw mxa R2 = 0.552
aCGH
in silico
4
3
2
1
0
−1
−2
−3
−4
−4
−3
−2
−1
0
1
2
3
4

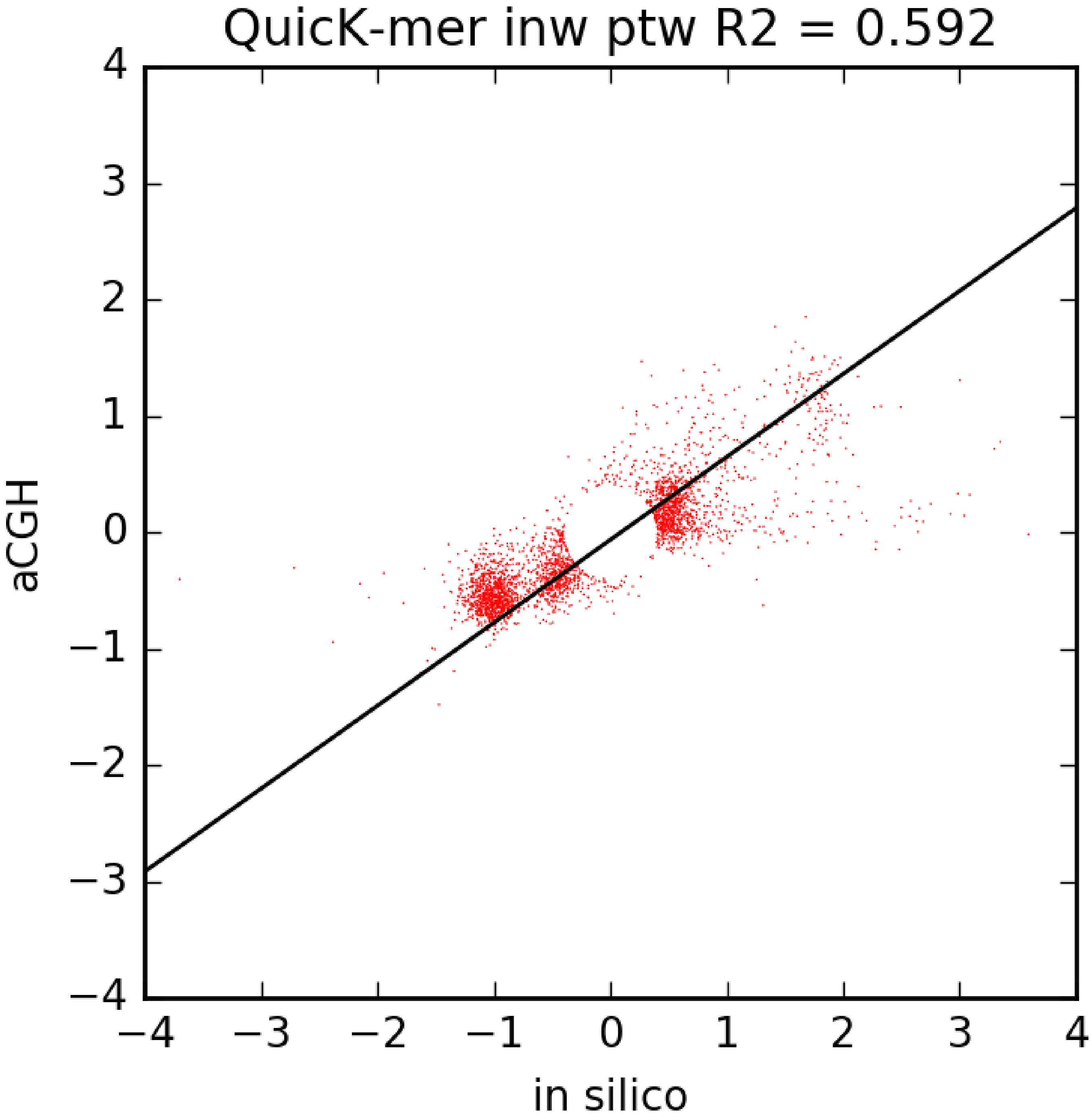
QuicK-mer inw ptw R2 = 0.592
aCGH
in silico
4
3
2
1
0
−1
−2
−3
−4
−4
−3
−2
−1
0
1
2
3
4

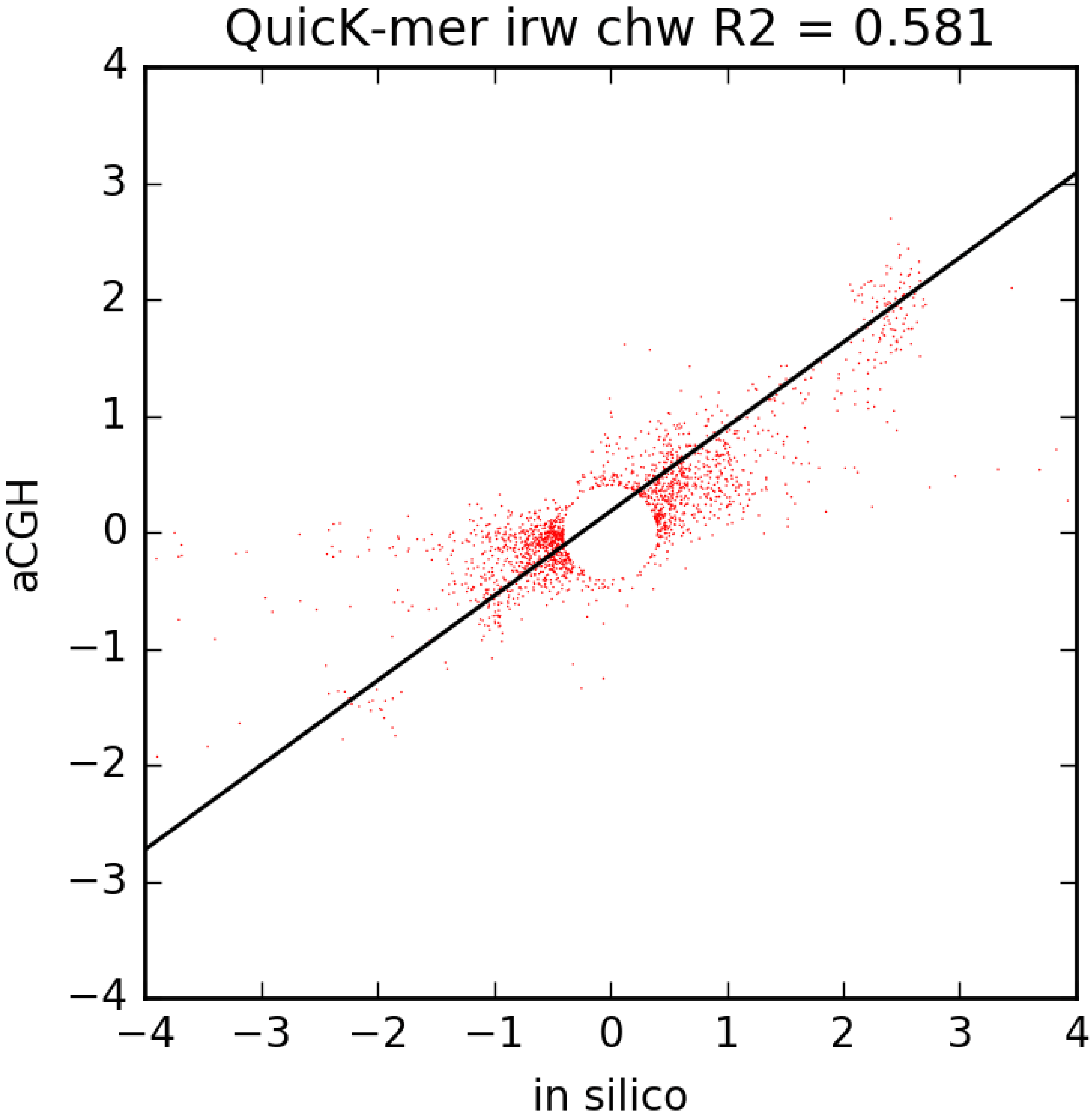
QuicK-mer irw chw R2 = 0.581
aCGH
in silico
4
3
2
1
0
−1
−2
−3
−4
−4
−3
−2
−1
0
1
2
3
4

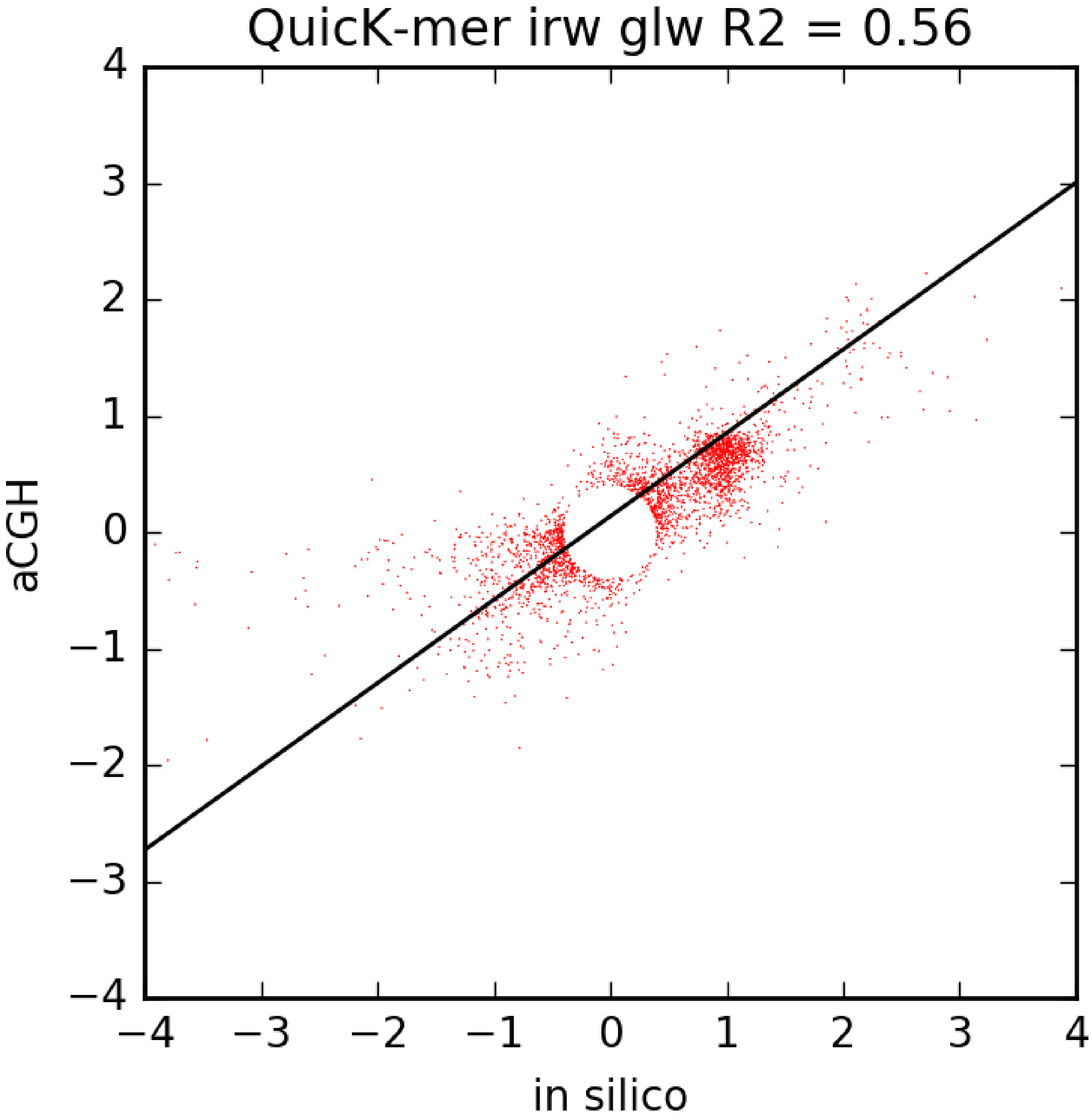
QuicK-mer irw glw R2 = 0.56
aCGH
in silico
4
3
2
1
0
−1
−2
−3
−4
−4
−3
−2
−1
0
1
2
3
4

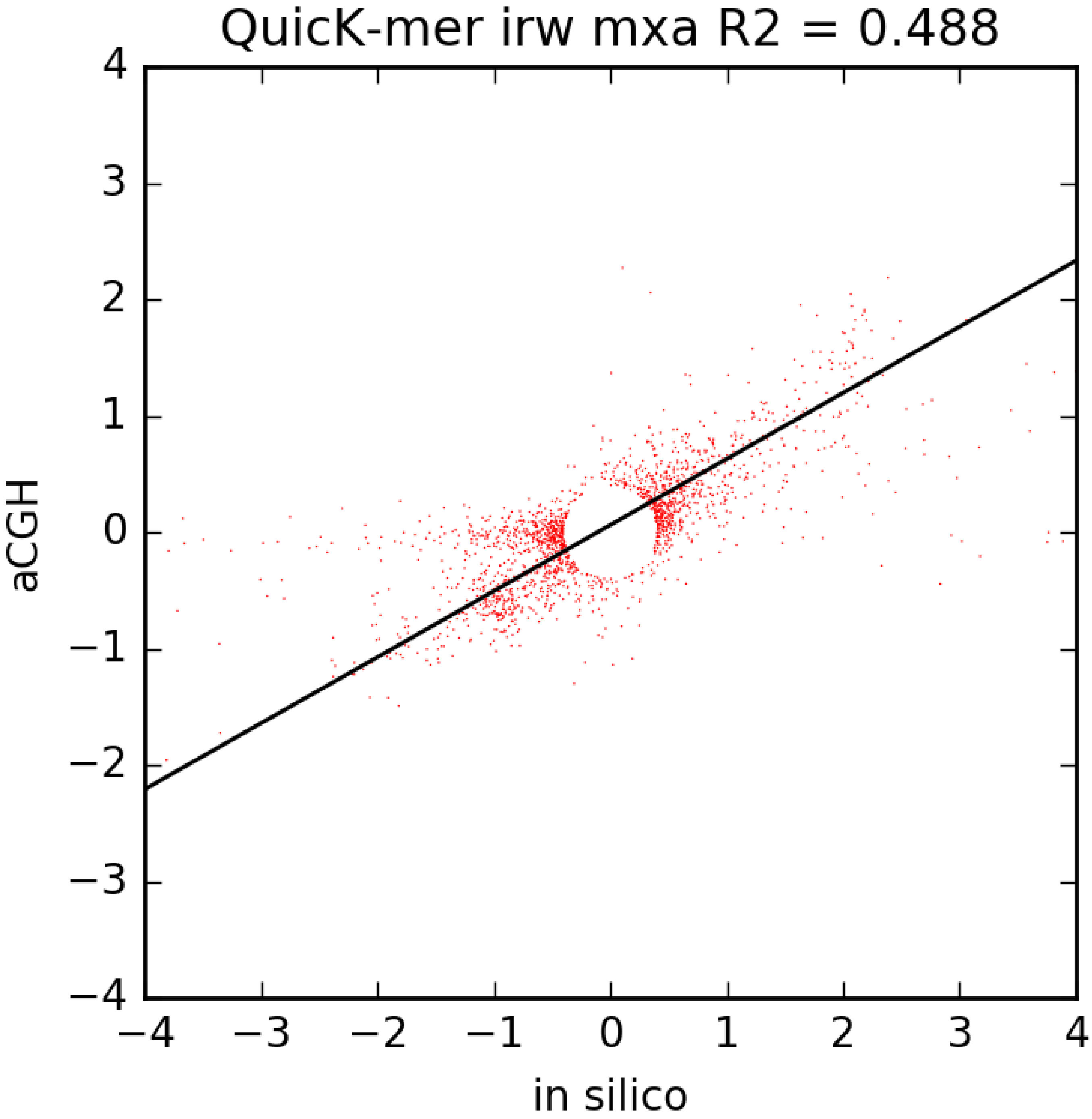
QuicK-mer irw mxa R2 = 0.488
aCGH
in silico
4
3
2
1
0
−1
−2
−3
−4
−4
−3
−2
−1
0
1
2
3
4

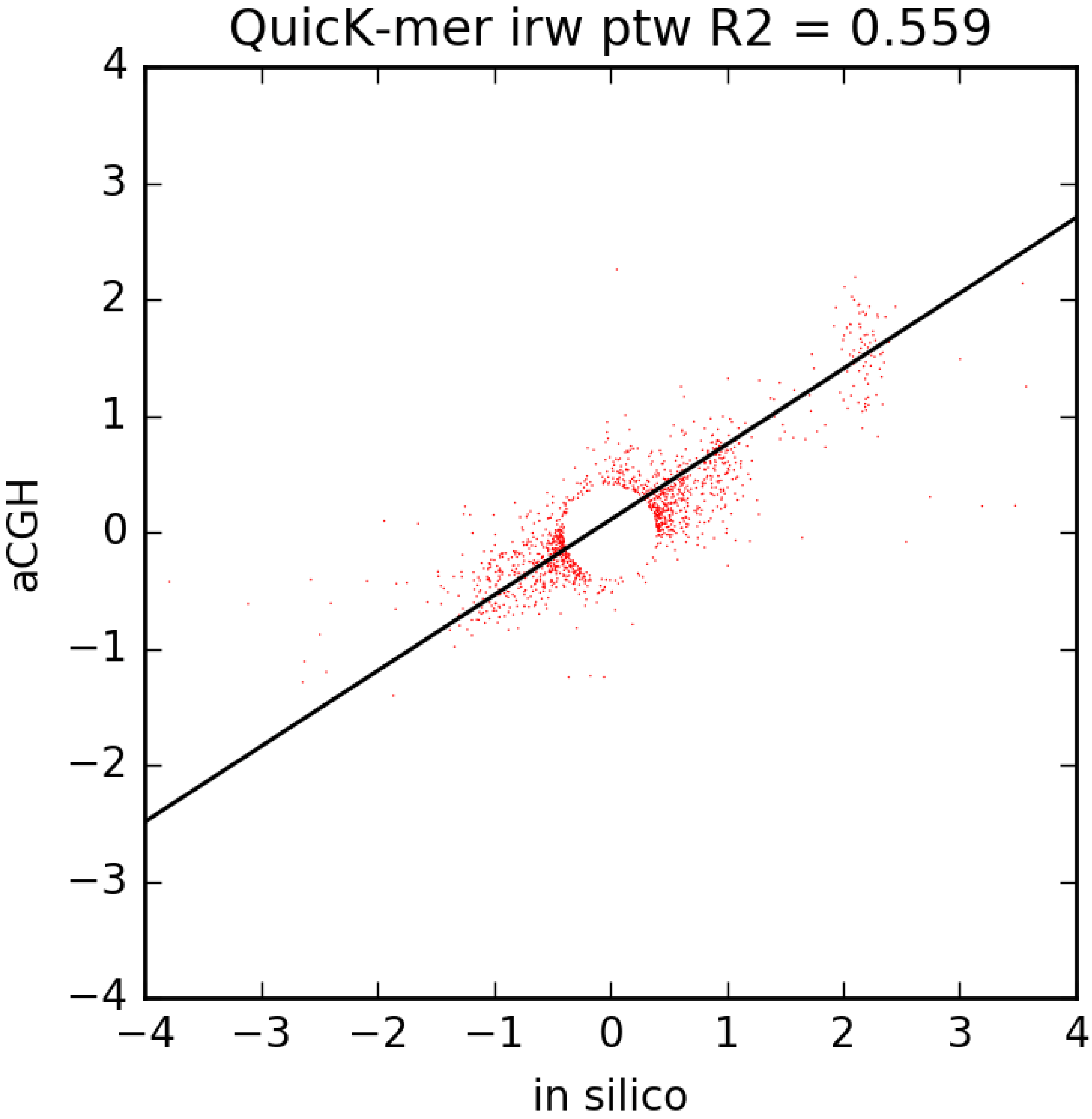

QuicK-mer irw ptw R2 = 0.559
aCGH
in silico
4
3
2
1
0
−1
−2
−3
−4
−4
−3
−2
−1
0
1
2
3
4

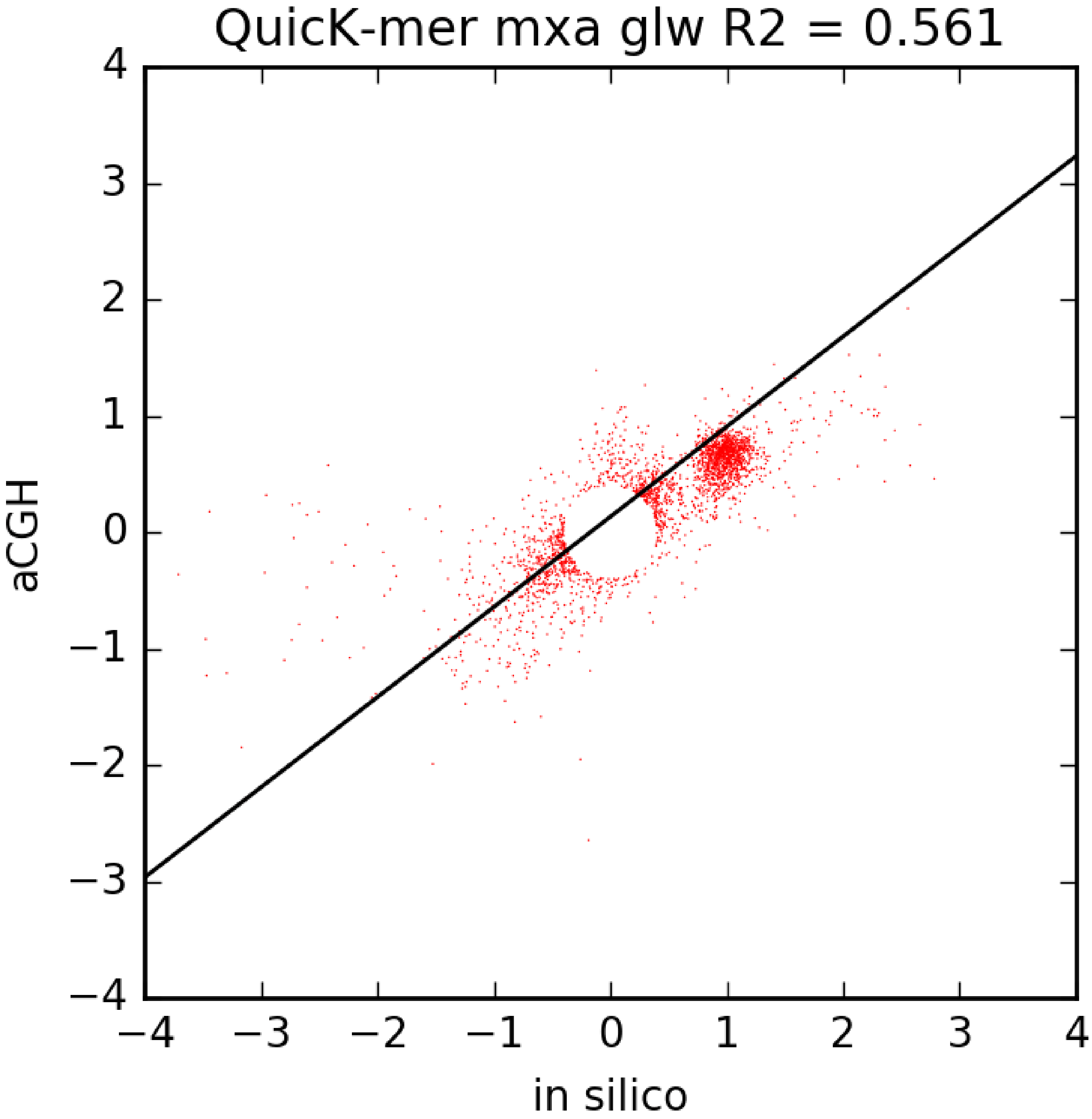

QuicK-mer mxa glw R2 = 0.561
aCGH
in silico
4
3
2
1
0
−1
−2
−3
−4
−4
−3
−2
−1
0
1
2
3
4

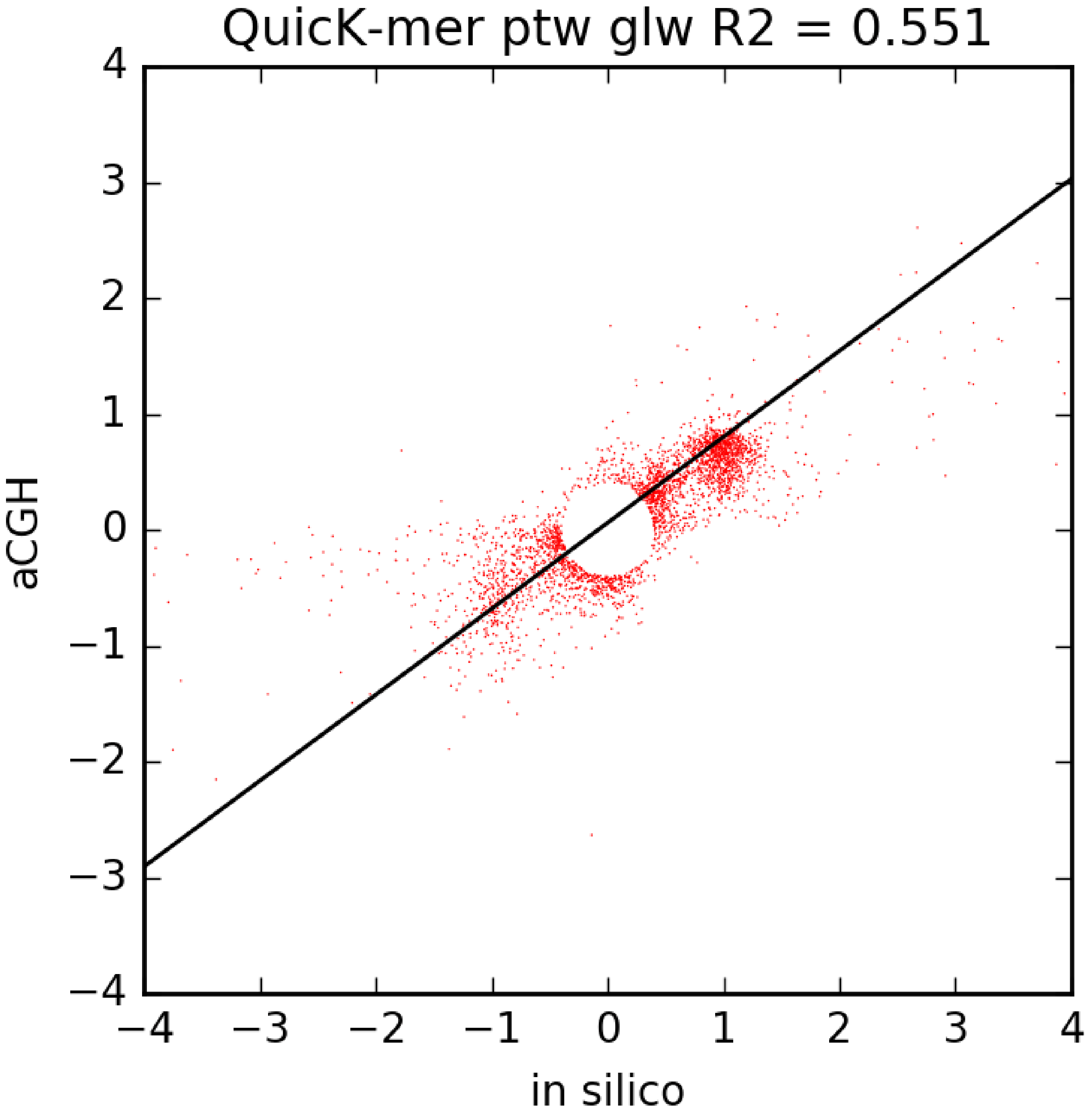

QuicK-mer ptw glw R2 = 0.551
aCGH
in silico
4
3
2
1
0
−1
−2
−3
−4
−4
−3
−2
−1
0
1
2
3
4

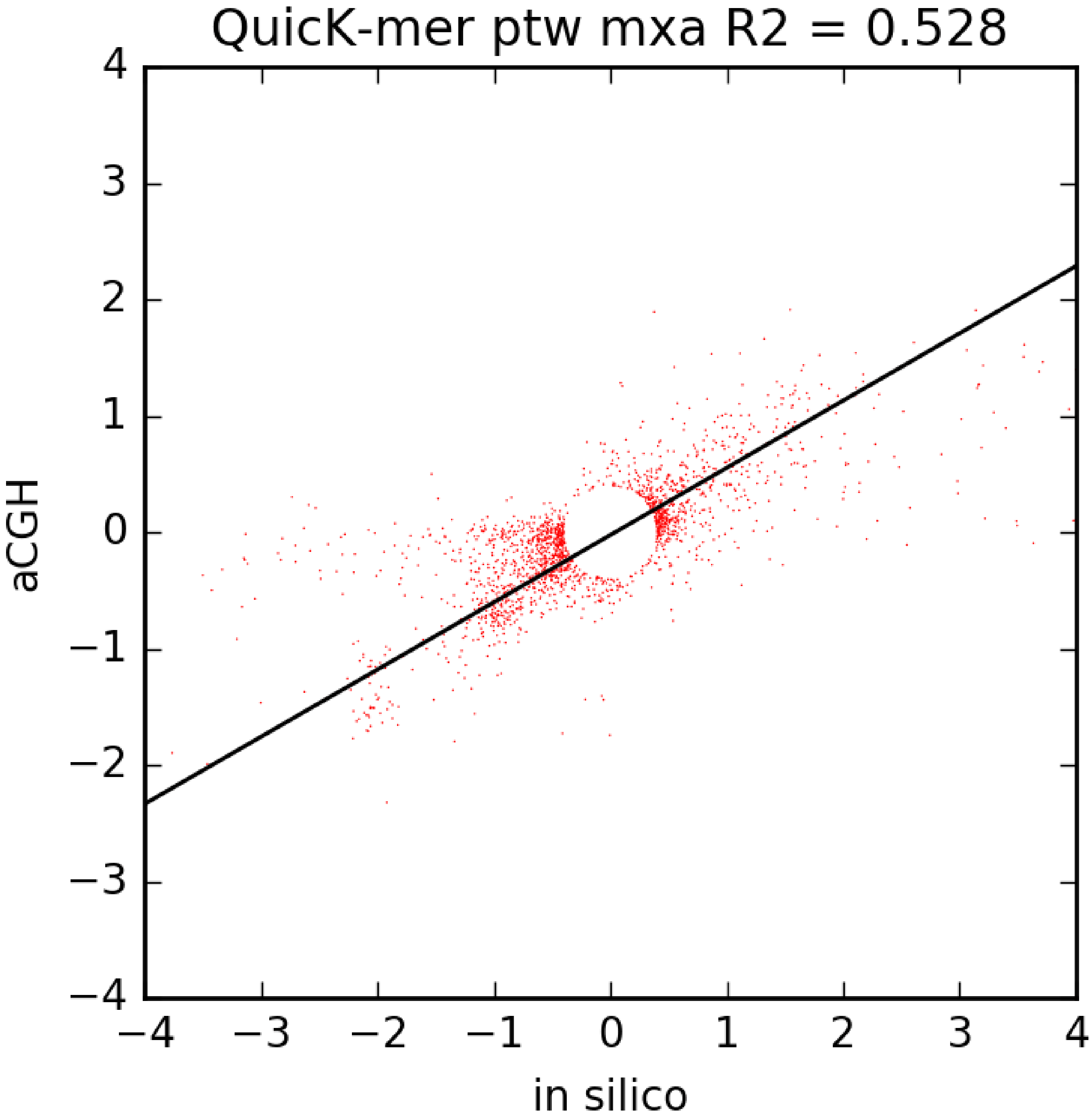
QuicK-mer ptw mxa R2 = 0.528
aCGH
in silico
4
3
2
1
0
−1
−2
−3
−4
−4
−3
−2
−1
0
1
2
3
4

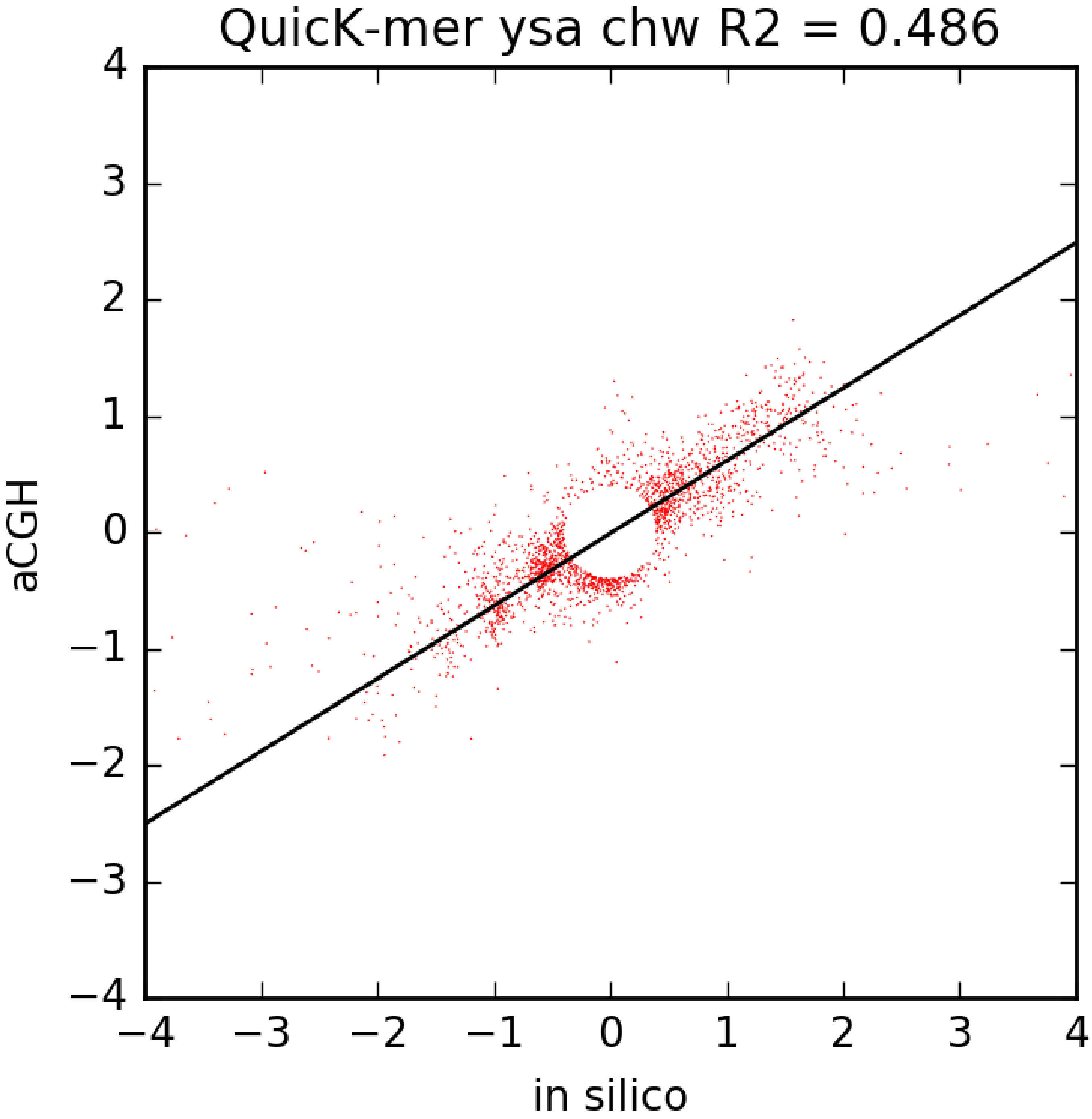

QuicK-mer ysa chw R2 = 0.486
aCGH
in silico
4
3
2
1
0
−1
−2
−3
−4
−4
−3
−2
−1
0
1
2
3
4

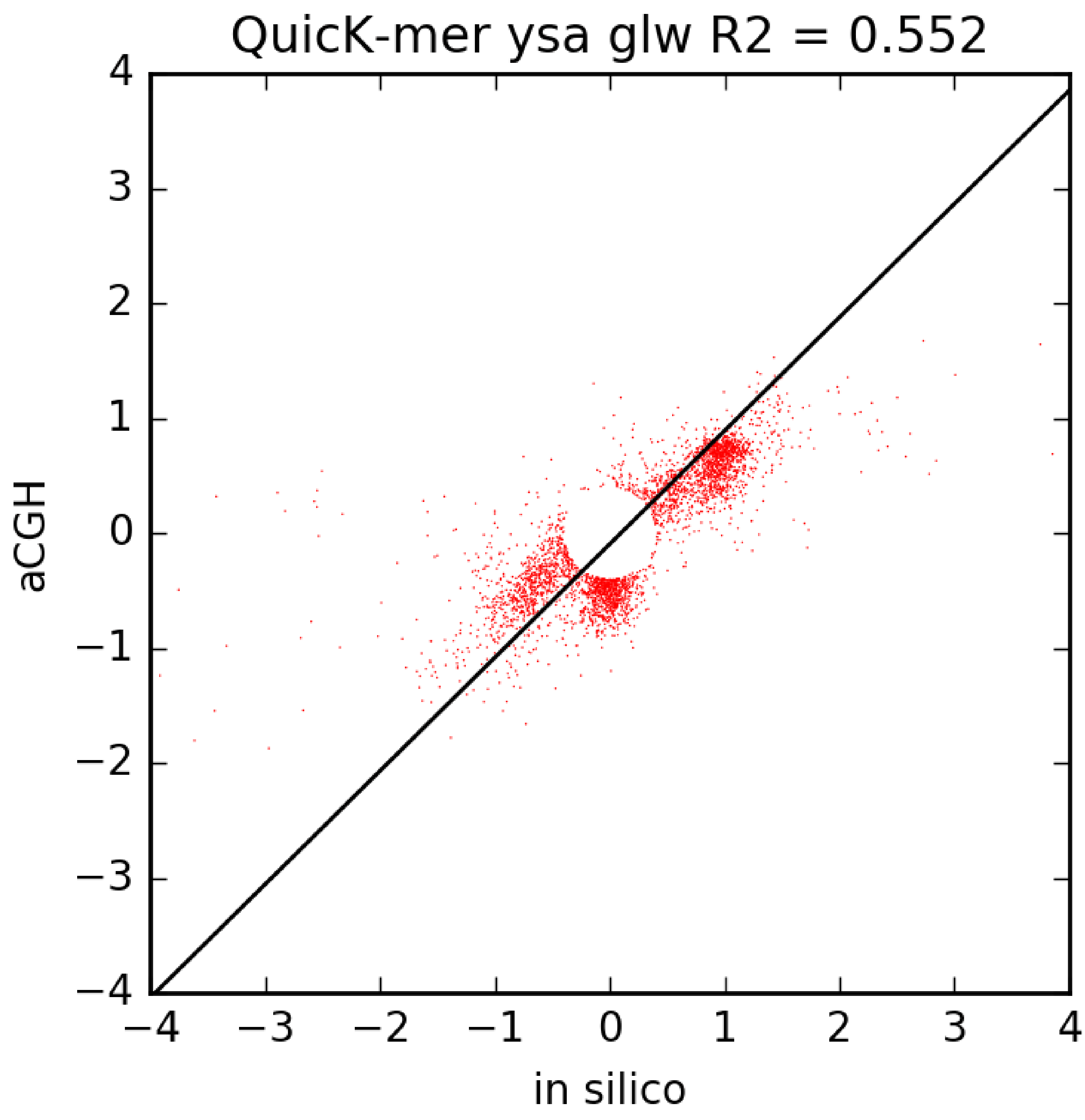
QuicK-mer ysa glw R2 = 0.552
aCGH
in silico
4
3
2
1
0
−1
−2
−3
−4
−4
−3
−2
−1
0
1
2
3
4

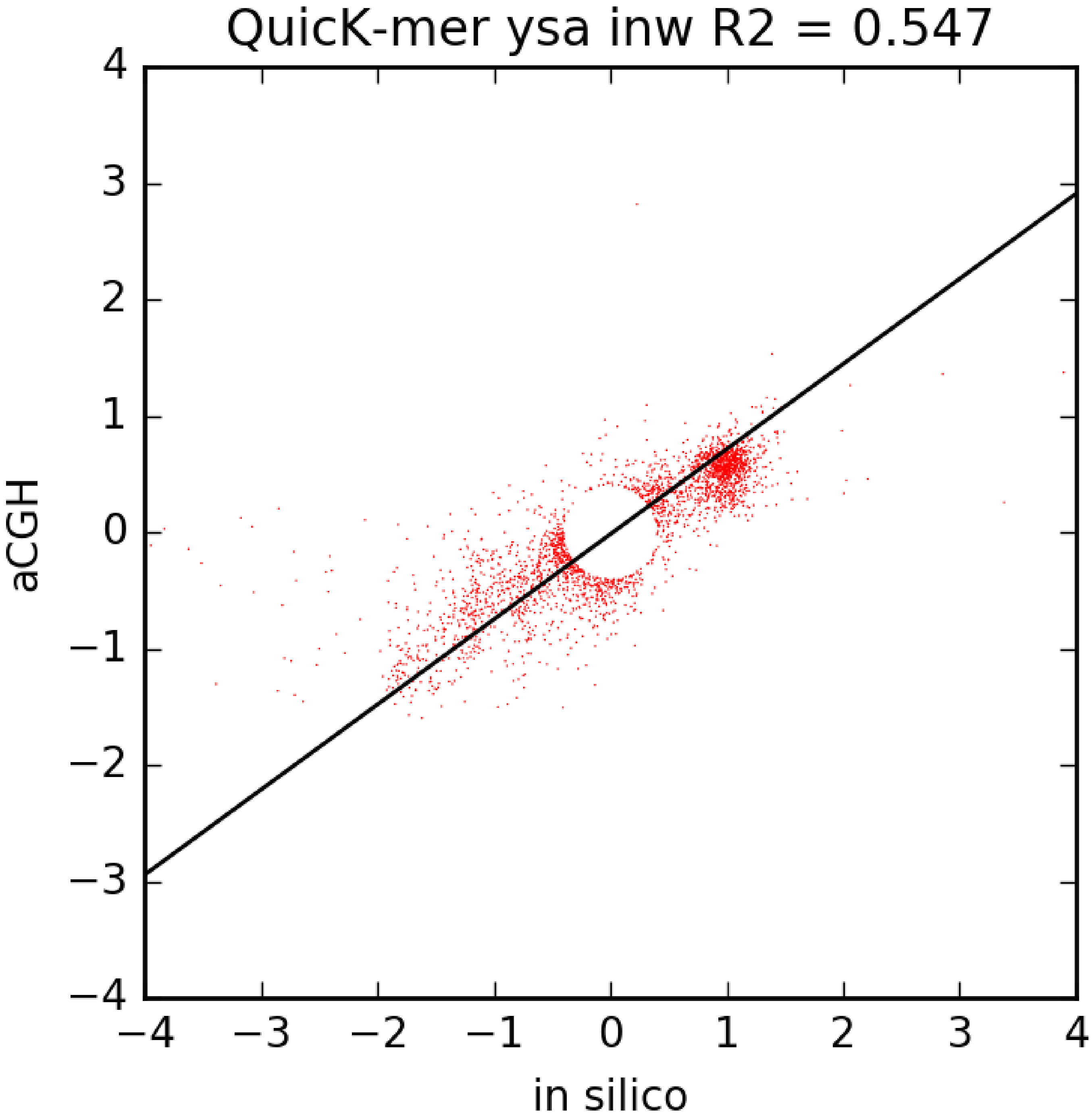

QuicK-mer ysa inw R2 = 0.547
aCGH
in silico
4
3
2
1
0
−1
−2
−3
−4
−4
−3
−2
−1
0
1
2
3
4

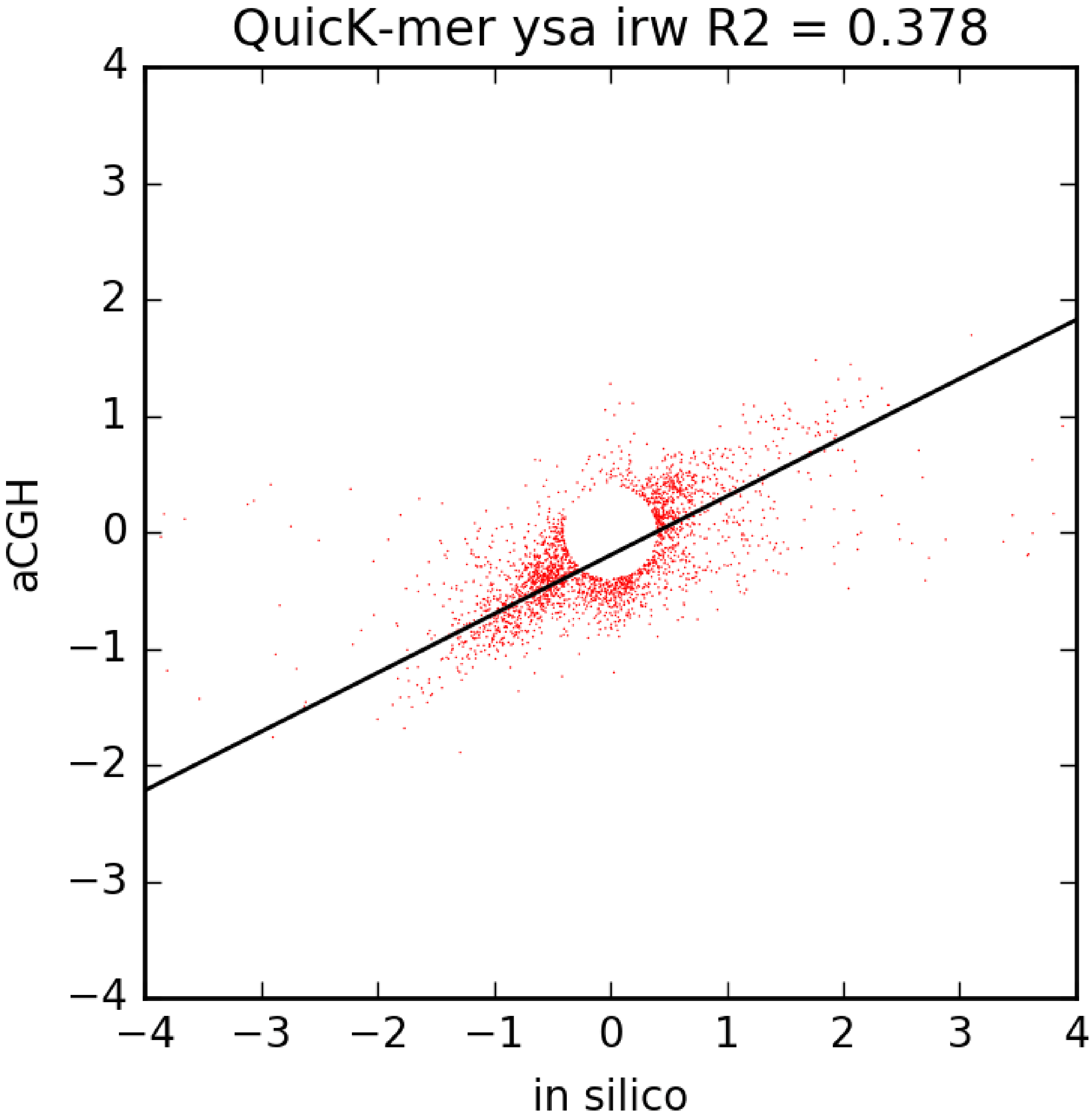

QuicK-mer ysa irw R2 = 0.378
aCGH
in silico
4
3
2
1
0
−1
−2
−3
−4
−4
−3
−2
−1
0
1
2
3
4

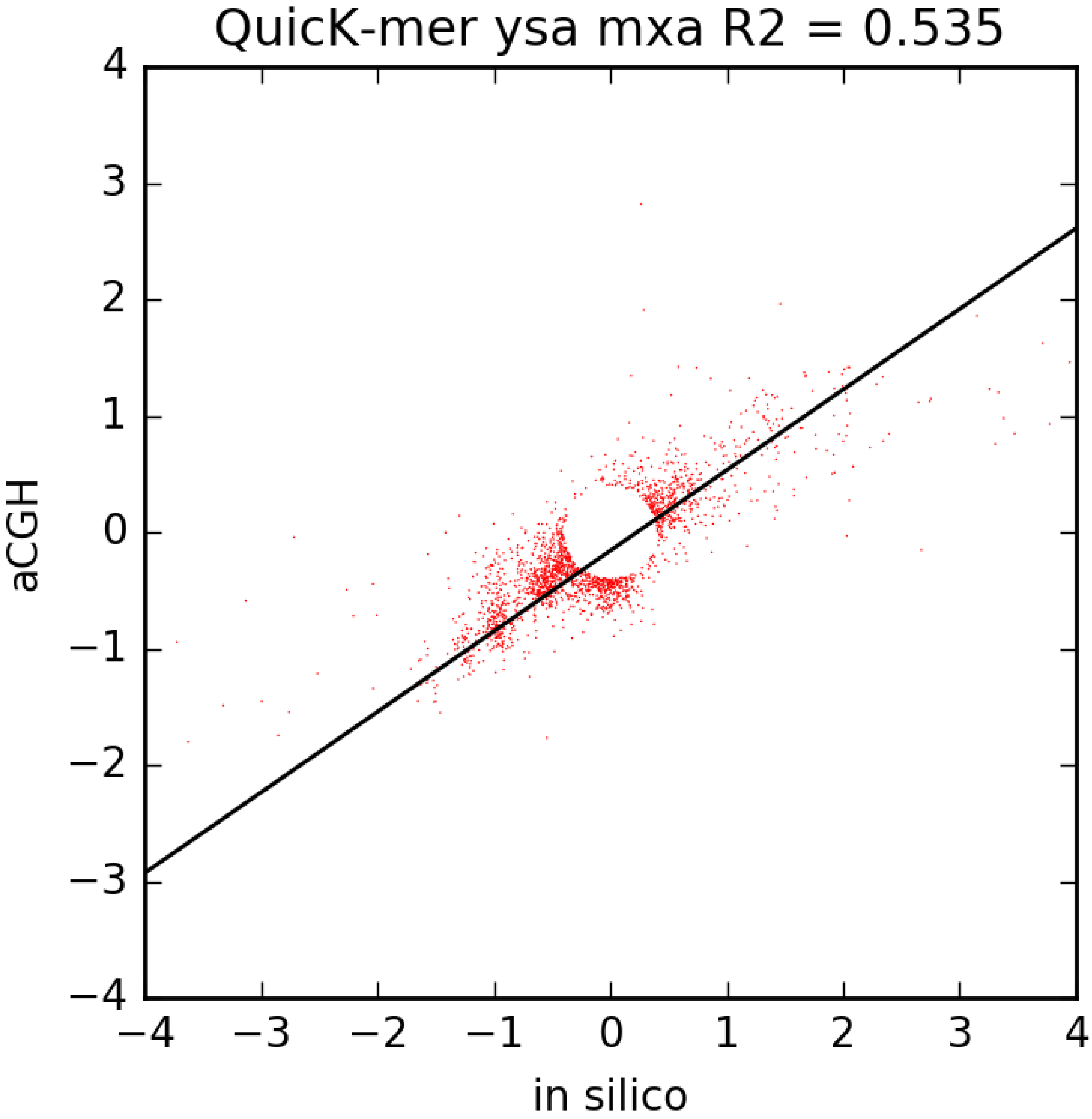

QuicK-mer ysa mxa R2 = 0.535
aCGH
in silico
4
3
2
1
0
−1
−2
−3
−4
−4
−3
−2
−1
0
1
2
3
4

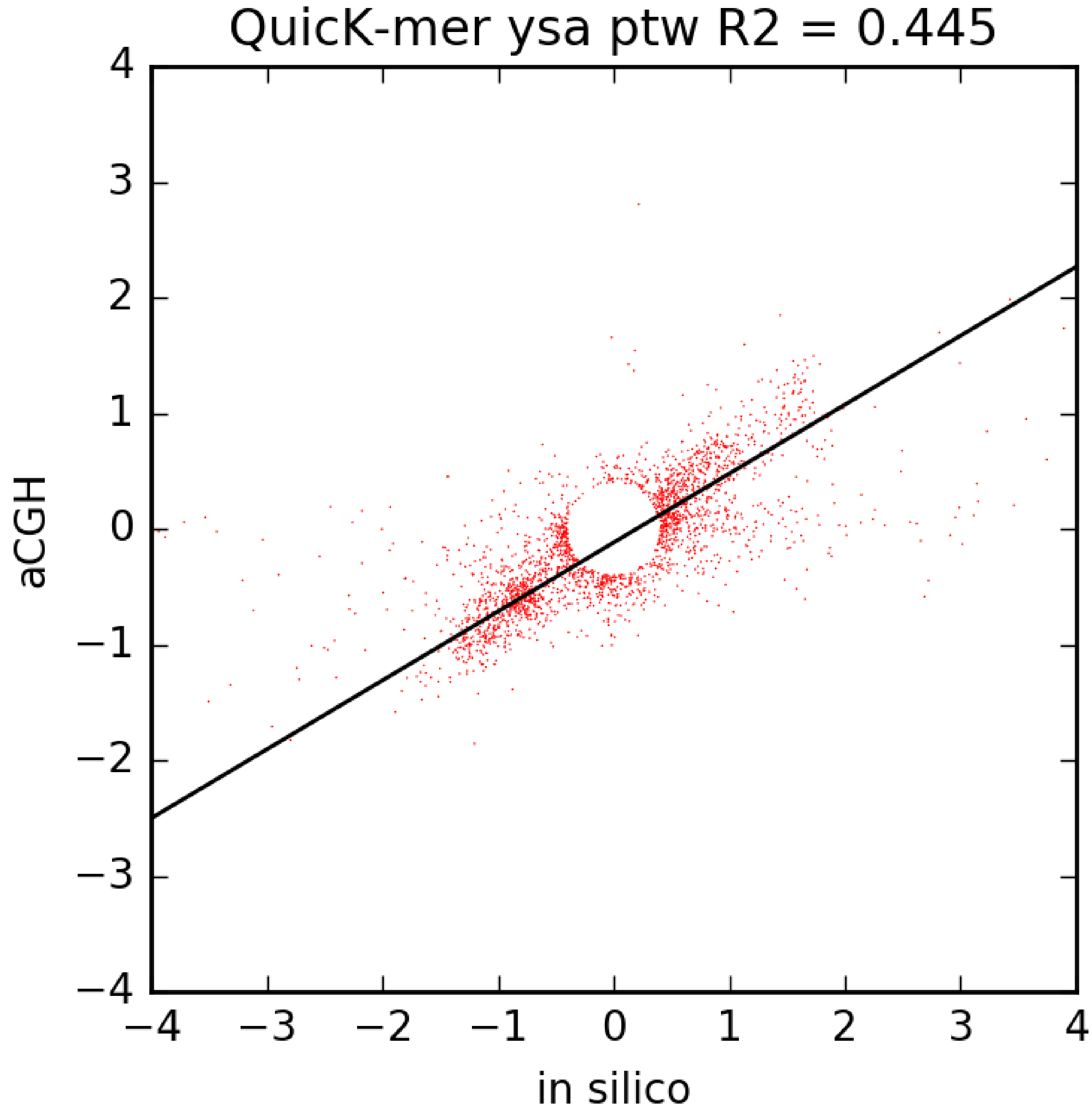
QuicK-mer ysa ptw R2 = 0.445
aCGH
in silico
4
3
2
1
0
−1
−2
−3
−4
−4
−3
−2
−1
0
1
2
3
4
